# Isoelectronic Push–Pull Fluorescent Difluoroborates: Halogen Bonding and Photophysical Properties

**DOI:** 10.1021/acs.joc.4c03077

**Published:** 2025-02-11

**Authors:** Alex Iglesias-Reguant, Izabela Barańska, Damian Plażuk, Robert Zaleśny, Josep M. Luis, Borys Ośmiałowski

**Affiliations:** † Faculty of Chemistry, Nicolaus Copernicus University, Gagarina Street 7, 87-100 Toruń, Poland; ‡ Laboratory of Molecular Spectroscopy, Department of Organic Chemistry, Faculty of Chemistry, University of Łódź, Tamka 12, 91-403 Łódź, Poland; § Faculty of Chemistry, Wroclaw University of Science and Technology, Wybrzeże Wyspiańskiego 27, 50-370 Wrocław, Poland; ∥ Institute of Computational Chemistry and Catalysis and Department of Chemistry, University of Girona, Campus de Montilivi, Girona, Catalonia 17003, Spain

## Abstract

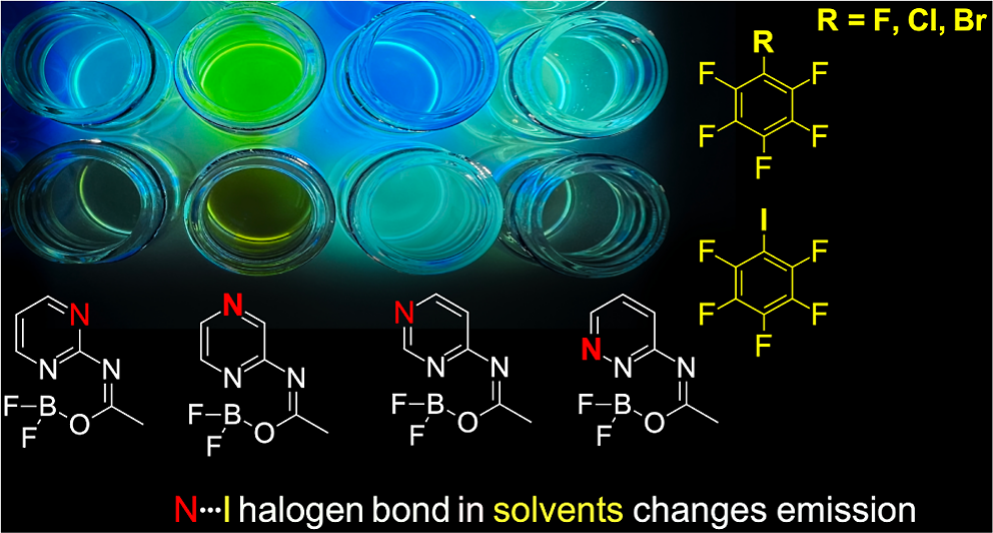

The structural and photophysical properties in the halogen bonding environment were thoroughly studied for a newly synthesized series of fluorescent dyes and their model derivatives. The analysis revealed that the ground-state interactions among both series are likewise. The fluorescent dyes have push–pull topology, and there is a low-lying charge-transfer (CT) excited state in their electronic structure. In order to study the effect of intermolecular interactions on the photophysical parameters of the CT excited state, a palette of solvents was used (C_6_F_6_, C_6_F_5_Cl, C_6_F_5_Br, and C_6_F_5_I). Our studies revealed that the weak halogen bonding between the perfluorohaloarene solvent and the heterocyclic core of the dyes enhances the CT in their excited states. The results also demonstrated that the position of the heterocyclic nitrogen atom in the acceptor core simultaneously controls the directionality of the intermolecular interaction and influences both the emission wavelength and the fluorescence quantum yield. Experimental data were further supported by the results of quantum-chemical calculations. Overall, the study establishes a direct link between the topology of a moiety prone to specific intermolecular interactions and the photophysical properties of fluorescent probes.

## 1 Introduction

Fluorescent dyes are widely used across various technological fields, requiring optimization of their fundamental properties for specific applications. Several strategies can be employed to tune the properties of the emitting molecules. These include introducing substituent effects, extending conjugation, and incorporating charge-transfer (CT) properties by placing donor and acceptor moieties within the molecular structure. Additionally, atom exchange within the fluorescent core and, to a lesser extent, intermolecular interactions can influence the behavior of fluorescent molecules.

Noncovalent interactions, such as hydrogen bonding,^1−3^ halogen bonding,^4−6^ and weaker forces, originating from the molecular environment near the fluorophore, often influence the magnitude of observed solvatochromic shifts. These interactions also play a crucial role in sensing applications,^7−10^ where reversible intermolecular interactions lead to the formation and dissociation of complexes resulting in *on*/*off* mechanisms based on the two states of a fluorophore.^11−14^ Key characteristics related to sensing mechanisms include (a) protonation and deprotonation of sensing groups attached to the fluorophore,^15^ (b) hydrogen bonding,^16,17^ and (c) halogen bonding interactions.^16,18^ Intermolecular interactions are also considered in the design of light-emitting devices.^19−21^ However, intermolecular interactions, such as π–π stacking, often lead to emission quenching.^22,23^ Nevertheless, there exist established protocols to mitigate these effects and preserve emission efficiency.^24−26^

CT properties in dyes, typically introduced through electron-donating/accepting groups and the system of π-electrons, provide a highly sensitive tool for sensing applications.^27,28^ The knowledge of self-organization of push–pull molecules^29^ is important to material science as there is a strong dependence of CT properties on structural parameters.^30^ These dyes often exhibit solvatochromic behavior, where significantly larger shifts are found for the emission band compared to the absorption band. This shift, typically toward the red region of the spectrum,^31^ is observed when a π–π* transition is involved and the polarity of the surrounding environment increases. However, substantial red-shifts can lead to a reduction in the emission efficiency, following the energy-gap law. Consequently, extending the π-conjugation is generally not beneficial for maximizing emission efficiency or preventing molecular stacking.

A decrease in emission quantum yield can result not only from intermolecular interactions but also from the intrinsic structural properties of the dye, such as conformational flexibility.^32,33^ The conformational flexibility arises from the rotation around specific bonds within the emitter,^34,35^ ring puckering,^36^ molecular skeleton bending in solids,^37^ amine group inversion or rotation^38^ (which can sometimes lead to twisted intramolecular CT, or TICT^39^), and even trans–cis photoisomerization^40^ due to the presence of carbon–carbon double bond. Molecular size is another important consideration. Smaller molecules are often more desirable for applications due to their enhanced solubility, ease of material penetration (e.g., polymers and tissues), and reduced steric effects. In this context, relatively small dyes with red-shifted emission and sensing capabilities represent attractive targets for further exploration.

**Figure 1:**
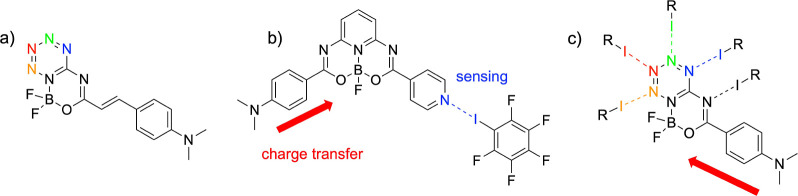
Comparison of the topology of previously studied,^41^ isoelectronic dyes (a), dye sensitive to XB,^42^ (b) and the current **xPN** series (c).

Recent work by some of the authors of the present study demonstrated that altering the position of nitrogen atoms in the heterocyclic ring of a series of isomeric CT-exhibiting difluoroborates affects the fluorescence quantum yield (Φ_*f*_) in systems shown in Figure 1a.^41^ It has also been shown that a specific halogen bond (XB) in a CT pyridine-functionalized fluoroborate dye has a similar impact on Φ_*f*_ (Figure 1b).^42^ The current study aims to explore changes in the photophysical properties of new series of isoelectronic CT-exhibiting difluoroborates (Figure 1c) due to halogen bonding at different positions. Unlike previously studied pyridine-functionalized fluoroborate (Figure 1b), the difluoroborates of this study were designed to include the halogen bond acceptor positioned near the BF_2_ acceptor within the CT path.

**Figure 2:**
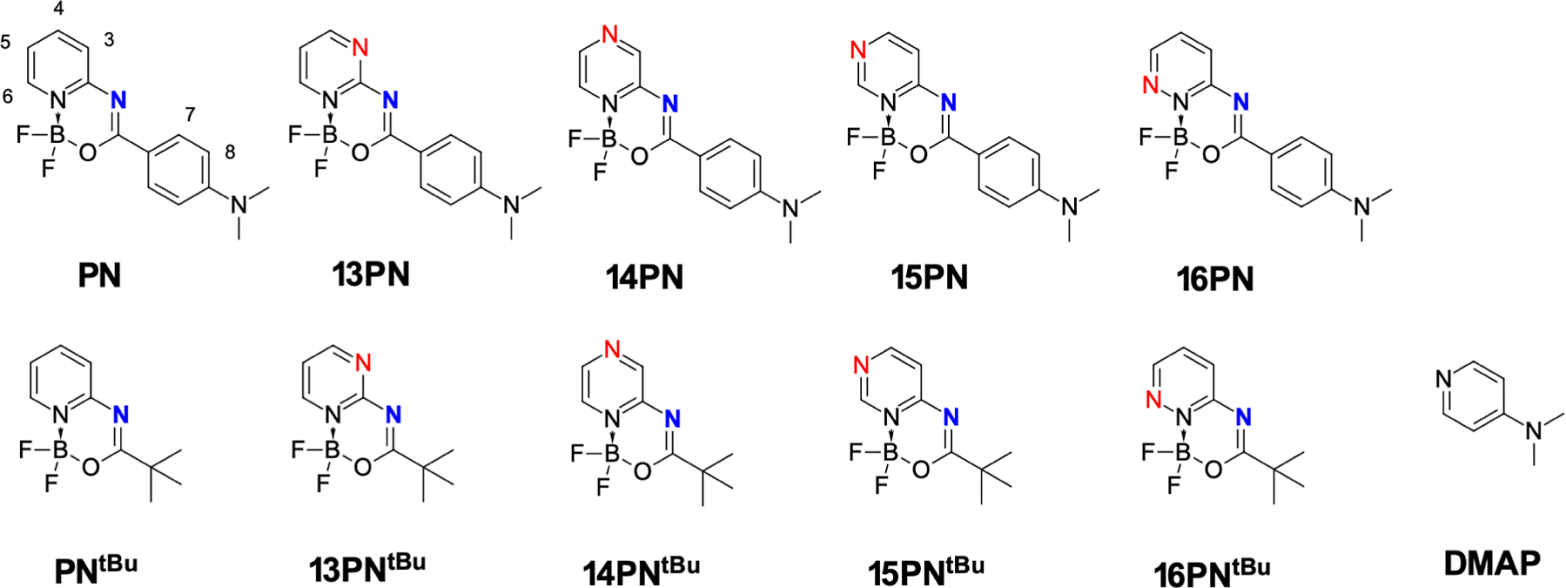
Structure of the dyes (first row) and the model compounds (second row) studied.

The newly synthesized series of heterocyclic dyes (**PN**, **13–16PN**) are depicted in Figure 2. The dyes possess a dipolar structure with an N–BF_2_–O electron-accepting group and a *N*,*N*-dimethylamino electron-donating group connected by a single bond. For comparison purposes, additional measurements were performed by using model molecules. In these models, the donor part was represented by 4-(*N*,*N*-dimethylamino)pyridine (**DMAP**), while the acceptor/interacting part was represented by the *t*-Bu-substituted molecules (**PN**^***tBu***^, **13–16PN**^***tBu***^). The study was conducted across a range of solvents, each differing by a single halogen atom (C_6_F_6_, C_6_F_5_Cl, C_6_F_5_Br, and C_6_F_5_I). The investigation of the photophysical properties was preceded by a study of intermolecular interactions in the ground state using nuclear magnetic resonance (NMR) spectroscopy, while density functional theory (DFT) calculations provided further insight into the behavior of these dyes in both ground (GS) and excited states (ES).

It is important to note that the intermolecular interactions considered involved N···I halogen bonds at the heterocyclic nitrogen atom (indicated in red in Figure 2) and the imine nitrogen in the boron-containing ring (in blue in Figure 2). Interactions at the *N*,*N*-dimethylamino group were not considered, as previous studies^42^ have proved that this group is inert to halogen bonding due to the bulkiness of the methyl groups, the rotational flexibility of the amine group, and its tendency to inversion. Similarly, interactions involving the oxygen atom were excluded as electronic repulsion of the close fluorine atoms and their high electronegativity make the formation of an F_2_B–O···I bond unlikely.

## 2 Results and Discussion

### 2.1 Halogen-Bonded Complexes in the Ground State

All titrations revealed low association constants (below 10 M^–1^) indicating weak intermolecular interactions between the dye and the halogen bond donor. This finding is consistent with our previous studies^42^ and further supported by the earlier work of Taylor et al.^43,44^ The low association constants can be attributed to the strong electron-accepting properties of the BF_2_ group, which significantly reduces the electron density (Figure S54) at the nitrogen atom suitable for halogen bonding. The titration curves (Figures S41–S44) and complexation-induced shifts (CIS, Figure S45)—calculated as the difference between the chemical shift at the maximum [guest]/[host] ratio and that of the neat host—offer insights into the nature of the molecular complexes formed.

The ^1^H NMR titration using C_6_F_5_I as the guest molecule exhibited a typical behavior of chemical shifts for all protons, although the CISs were minimal for some of them. The insignificant values of CIS can be attributed to either the low sensitivity of certain protons to the intermolecular halogen bond or their distance from the interaction site. However, to further confirm the interaction, an additional guest was used, namely, C_4_F_9_I. In this case, a nonuniform variation in chemical shift changes was observed in the ^1^H NMR titration. While some titration curves followed the typical behavior reported for supramolecular complexes—showing an increase in the chemical shift for protons located near the interacting atom—others exhibited a different trend. Specifically, these trends included a linear decrease or even a sigmoidal pattern (Figure S41, **PN** titrated by C_4_F_9_I) in the chemical shift changes, suggesting a more complex equilibrium or a significant bulk solvent effect influencing the chemical shifts. For certain protons, such as H8 in **13PN** or **14PN** (Figures S42 and S43), the observed changes were minimal and could be considered negligible. In the case of C_4_F_9_I, the largest CIS values for protons in **PN** were observed for H3 and H7 (see Figure 2 for the labeling), suggesting interaction at the imine-nitrogen site (Figure S45). A deshielding effect was observed for protons H4 and H7 in **13PN**, H3, H5, and H7 in **14PN**, and H6 and H7 in **15PN**, confirming the involvement of the imine-nitrogen site and the heterocyclic nitrogen atom of the diazine ring in halogen bonding. A comparison of the CIS values for C_6_F_5_I and C_4_F_9_I further demonstrated the distinct nature of interactions between these guests. The presence of π–π stacking and halogen bonding in complex with C_6_F_5_I aligns with the chemical intuition. However, to keep the consistency in measurements (single halogen substitution in aryl-based solvents), the alkyl-based solvent was not used in photophysical studies of the dyes.

The previous study^45^ suggested that ^15^N NMR data are not ideal for characterizing halogen bonding in solution. However, other studies report changes in ^15^N chemical shifts due to halogen bonding in the intramolecular domain, specifically for molecules designed to promote such interactions (Figure S53).^46^ Thus, to further elucidate the molecular complexes formed by the **xPN** series of dyes in various solvents, we recorded ^1^H–^15^N HMBC spectra for all dyes. Table 1 summarizes the changes in ^15^N chemical shifts for the heterocyclic nitrogen and the NMe_2_ moiety as the solvent is varied from C_6_F_6_ to C_6_F_5_I. The chemical shifts of the imine nitrogen are not reported as its distance from any hydrogen prevents the generation of a sufficiently strong cross-peak signal. The values for Δδ for *t*-Bu derivatives in C_4_F_9_I (dyes were not soluble enough in this solvent) are very similar, specifically: −2.5, −3.3, −1.2, −0.7, and −12.7/7.3 for **DMAP** (sequence as shown in Table 1).

**Table 1:** Change in ^15^N NMR Chemical Shift upon Change of the Solvent from C_6_F_6_ (Reference) to C_6_F_5_I (Experimental Data)

compound	Δδ [ppm] for heterocyclic nitrogen	Δδ [ppm] for NMe_2_ nitrogen
**13PN**	–4.8	2.9
**14PN**	–4.7	2.3
**15PN**	–3.7	3.6
**13PN**^***tBu***^	–2.5	
**14PN**^***tBu***^	–4.6	
**15PN**^***tBu***^	–2.4	
**16PN**^***tBu***^	–1.2	
**DMAP**	–15.4	5.9

The observed shielding of the heterocyclic nitrogen in a halogen-bonding environment indicates the formation of the interaction, further corroborated by the deshielding of nitrogen in the NMe_2_ group, which suggests a change in the electron density at the electron-rich nitrogen atom. The results for **16PN**^***tBu***^ show the smallest change in shift, and it is assumed that the same applies for **16PN**, a compound with too low solubility to record ^1^H–^15^N HMBC spectra. Furthermore, ^1^H–^15^N HMBC spectra were recorded for **DMAP** as it mimics the electron-donor part of the studied dyes. For **DMAP**, a ^15^N chemical shift change of −15.4 ppm (heterocyclic nitrogen, shielding) was observed, confirming the presence of halogen bonding. In contrast, the chemical shift of the NMe_2_ nitrogen atom in **DMAP** shifted in the opposite direction by 5.9 ppm. This confirms a similar effect in both the series of dyes and model **DMAP**. The larger chemical shift change in **DMAP**, compared to that in the dyes, is attributed to the absence of the strong electron-accepting BF_2_ moiety, allowing for stronger halogen bonding with the heterocyclic nitrogen. This is consistent with the low association constants observed for the dyes. In parallel, the opposite direction of the change of ^15^N chemical shifts for heterocyclic and *N*,*N*-dimethylamino nitrogens suggests similar electron density reorganization upon halogen bonding, namely, shift from the NMe_2_ donor toward the acceptor in the ground state.

**Figure 3:**
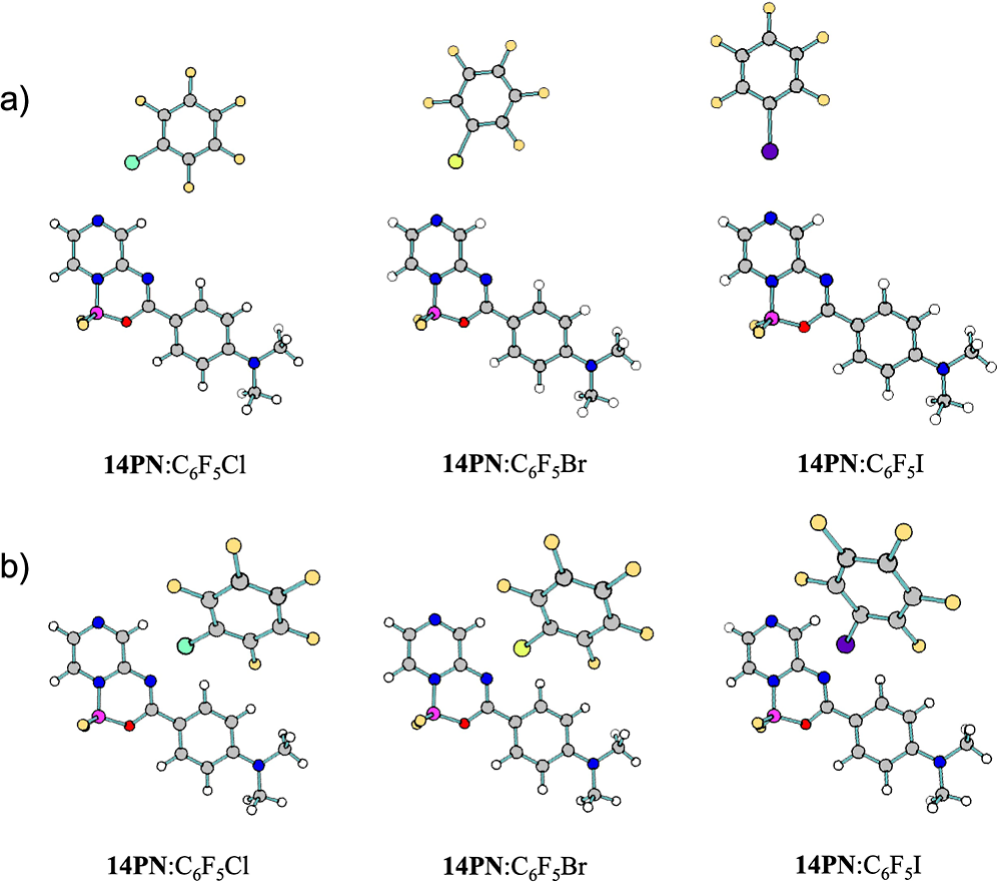
Equilibrium geometries of complexes formed by **14PN** and the halogen bond donors interacting at the (a) heterocyclic and (b) imine nitrogen atoms, obtained at the MN15/aug-cc-pVDZ(PP) level of theory.

To gain additional insight into the structures of the complexes, DFT geometry optimizations were performed. Two possible halogen bonding acceptor sites were considered: the heterocyclic nitrogen and imine nitrogen atoms. The equilibrium geometries of the complexes formed between the **14PN** dye and C_6_F_5_X (for X = Cl, Br, and I), interacting through both nitrogen positions, are shown in Figure 3. The geometries of the remaining complexes studied are depicted in Figures S48 and S49.

The ground-state optimizations revealed distinct structural properties depending on which nitrogen site was involved in the halogen bonding. The coplanarity of the donor and acceptor molecules is preserved when the interaction occurs at the heterocyclic nitrogen atom. However, the molecules are not coplanar when the interaction occurs at the imine nitrogen. Differences were also observed between complexes involving the same dye but different halogen bond donors. Specifically, as the halogen atom changes from chlorine (Cl) to bromine (Br) to iodine(I), the C–X···N angle becomes increasingly linear, approaching 180°, indicating a stronger and more directional halogen bond. An exception occurs in complexes involving **13PN**, where the proximity of two halogen bond acceptors seems to strengthen the interaction, resulting in larger angles (i.e., closer to 180°), even with C_6_F_5_Cl. These differences in the geometry may lead to distinct photophysical properties due to changes in the electronic distribution.

The formation Gibbs energy (Δ*G*) calculations were performed accounting for experimental concentrations (see below). As shown in Table 2, only complexes formed with C_6_F_5_I show negative Δ*G* values, indicating thermodynamically favorable halogen bonding.

**Table 2:** Formation Gibbs Energy (Δ*G*, in kcal/mol) Calculated for Complexes of **xPN** Series with C_6_F_5_X^a^

	heterocyclic N				imine N				
	**13PN**	**14PN**	**15PN**	**16PN**	**PN**	**13PN**	**14PN**	**15PN**	**16PN**
C_6_F_5_Cl	2.69	4.85	3.16	2.60	1.68		2.30	1.56	2.16
C_6_F_5_Br	0.30	1.77	1.86	1.36	1.88		0.40	0.13	–0.05
C_6_F_5_I	–1.37	–0.22	–0.13	–0.54	–1.06	–1.48	–0.81	–0.91	–1.08

a Results are presented for interactions at heterocyclic (columns 2–5) and imine (columns 6–10) nitrogen atoms.

The results obtained using DFT calculations suggest that while the halogen bonding with C_6_F_5_I stabilizes the complexes and leads to distinct changes in photophysics (vide infra), the interactions with C_6_F_5_Cl and C_6_F_5_Br are weak or thermodynamically unfavorable under the experimental conditions.

### 2.2 Photophysical Studies

**Figure 4:**
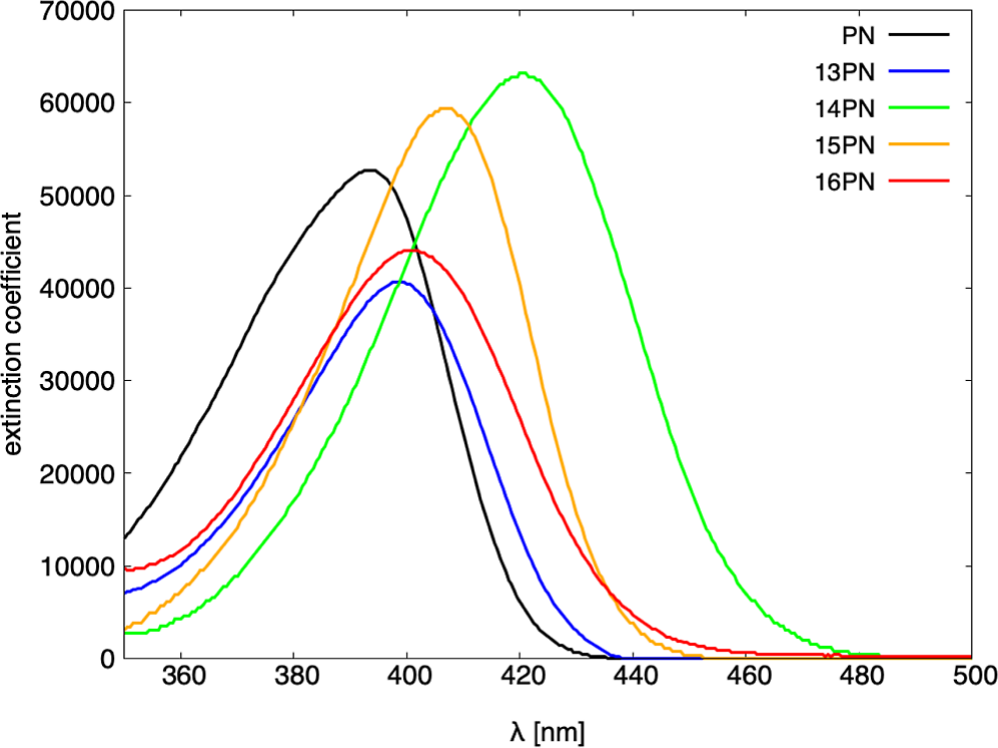
Absorption spectra for **PN**-**16PN** in C_6_F_6_.

The photophysical properties recorded for the **xPN** series in different solvents are summarized in Table 3 and Figures S50 and S51. Among the solvents used, C_6_F_6_ (Figure 4) was selected as the reference due to its neutral behavior regarding specific halogen bond intermolecular interactions. The data reveal that the position of the nitrogen atom within the heterocyclic ring significantly affects the absorption maximum wavelength (λ_abs_). For example, in C_6_F_6_, the absorption maximum varies across the violet-blue spectrum, ranging from 393 nm for **PN** to 420 nm for **14PN**. The trend in λ_abs_ remains consistent across the different solvents for each dye. The λ_abs_ value is the highest for C_6_F_5_I and the lowest for C_6_F_6_. Notably, all dyes exhibit a gradual red-shift in their absorption maxima when compared to the band in the spectra measured in C_6_F_6_ solvent, following the order C_6_F_5_Cl < C_6_F_5_Br < C_6_F_5_I. The largest red-shift, up to 15 nm, is observed for **14PN** in C_6_F_5_I. These findings demonstrate that altering the nitrogen position within the heterocyclic ring, combined with changing the solvent environment, allows for effective tuning of the absorption wavelength. This variability in absorption maxima can be considered significant, given the size of the isoelectronic dyes and the fact that it results from the change of only one atom in the solvents used. Together with the NMR results, these data strongly suggest that halogen bonding at the heterocyclic nitrogen significantly influences the CT properties of the **xPN** series.

**Table 3:** Photophysical Properties of the **xPN** Dye Series in Various Solvents at Room Temperature^a^

compound	solvent	λ_abs_ [nm]	λ_flu_ [nm]	FWHM_flu_ [cm^–1^]	Φ_f_	Stokes shift [cm^–1^]	τ [ns]
**PN**	C_6_F_6_	393	437	2304	0.88	2562	2.21
	C_6_F_5_Cl	395	435	2221	0.98	2328	2.06
	C_6_F_5_Br	397	438	2248	0.86	2358	1.93
	C_6_F_5_I	401	452	2637	0.08	2863	
**13PN**	C_6_F_6_	398	472	3847	0.36	3939	2.57
	C_6_F_5_Cl	400	465	3376	0.47	3495	3.77
	C_6_F_5_Br	402	472	3628	0.36	3689	3.18
	C_6_F_5_I	411	525	5464	0.02	5283	0.31
**14PN**	C_6_F_6_	420	517	3386	0.38	4467	2.77
	C_6_F_5_Cl	423	505	3227	0.64	3839	3.75
	C_6_F_5_Br	425	517	3373	0.59	4187	3.32
	C_6_F_5_I	435	578	4209	0.04	5740	0.30
**15PN**	C_6_F_6_	407	455	2127	0.92	2592	2.54
	C_6_F_5_Cl	409	452	2024	1.00	2326	2.35
	C_6_F_5_Br	412	456	2075	0.93	2342	2.34
	C_6_F_5_I	420	488	2623	0.67	3318	1.90
**16PN**	C_6_F_6_	400	513	4049	0.21	5507	2.45
	C_6_F_5_Cl	402	500	3820	0.40	4876	4.05
	C_6_F_5_Br	404	502	3634	0.41	4832	3.57
	C_6_F_5_I	411	555	4712	0.05	6313	0.29

a FWHM: full-width at half-maximum of the band; Φ_f_: fluorescence quantum yield; τ: fluorescence lifetime.

The calculated vertical absorption wavelengths (Table S1) align well with the experimental data, showing a red-shift as the halogen atom bonded to the heterocyclic nitrogen changes from Cl to Br to I in C_6_F_5_X—a trend consistent across all dyes. The time-dependent density functional theory (TD-DFT) predicted variations in absorption maxima for dyes interacting with C_6_F_5_I fall within a range of 21 nm (compared with 24 nm in the experiment), confirming the predictability and reliability of the computational approach. However, it is notable that the position of the simulated absorption band is blue-shifted by approximately 50 nm for C_6_F_5_I complexes interacting at the imine nitrogen atom (Table S2) relative to the experimental data. Therefore, the comparison between the computational and experimental absorption shifts suggests that the predominant halogen bond interaction occurs at heterocyclic nitrogen.

**Figure 5:**
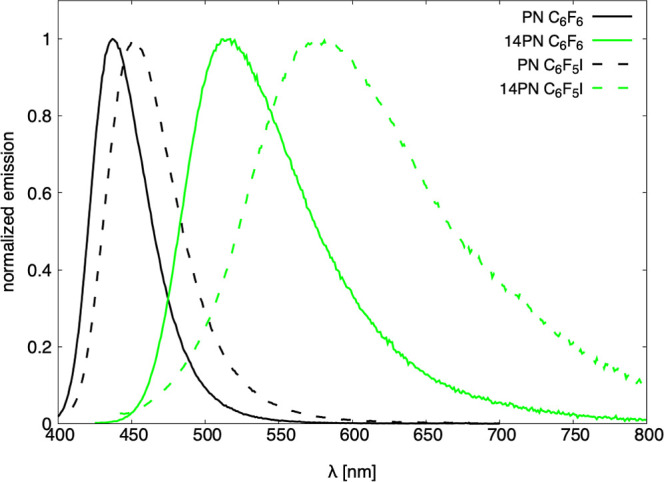
Fluorescence spectra for **PN** and **14PN** in C_6_F_6_ and C_6_F_5_I solvents.

The wavelength corresponding to the fluorescence band maximum (λ_flu_) is more sensitive to environmental changes than the absorption maximum wavelength. Across different solvents, the λ_flu_ values for the dyes cover a broad violet-to-blue range, from 437 nm for **PN** in C_6_F_6_ to 578 nm for **14PN** in C_6_F_5_I (Table 3 and Figure 5).

This difference is much larger than the 14 nm variation observed in the absorption band maxima. Additionally, the shift in λ_flu_ upon changing solvents from C_6_F_6_ to C_6_F_5_I is much more pronounced than for λ_abs_ and varies significantly between dyes. For instance, **PN** exhibits a modest 15 nm red-shift, while dyes with nitrogen in positions allowing halogen bonding without any steric hindrance show more significant shifts, ranging from 33 nm for **15PN** to 61 nm for **14PN**.

The computational approach was tested before time-consuming calculations were conducted for complexes in their ES. A comparative analysis was performed between experimental and computational results, focusing on the influence of halogen bonding and solvation (using the polarizable continuous model) on the emission spectra. This analysis concluded that calculations accounting for the effect of halogen bonding under vacuum are the most suitable for reproducing the experimental trends (Figure S52). Subsequently, calculations were carried out for complexes interacting at both the heterocyclic nitrogen and the imine nitrogen. Finally, to rationalize the relation of the absorption and emission processes with electron redistribution, we analyzed the electron density difference between the excited and ground states at both ground- and excited-state geometries for complexes interacting at the heterocyclic nitrogen.

**Figure 6:**
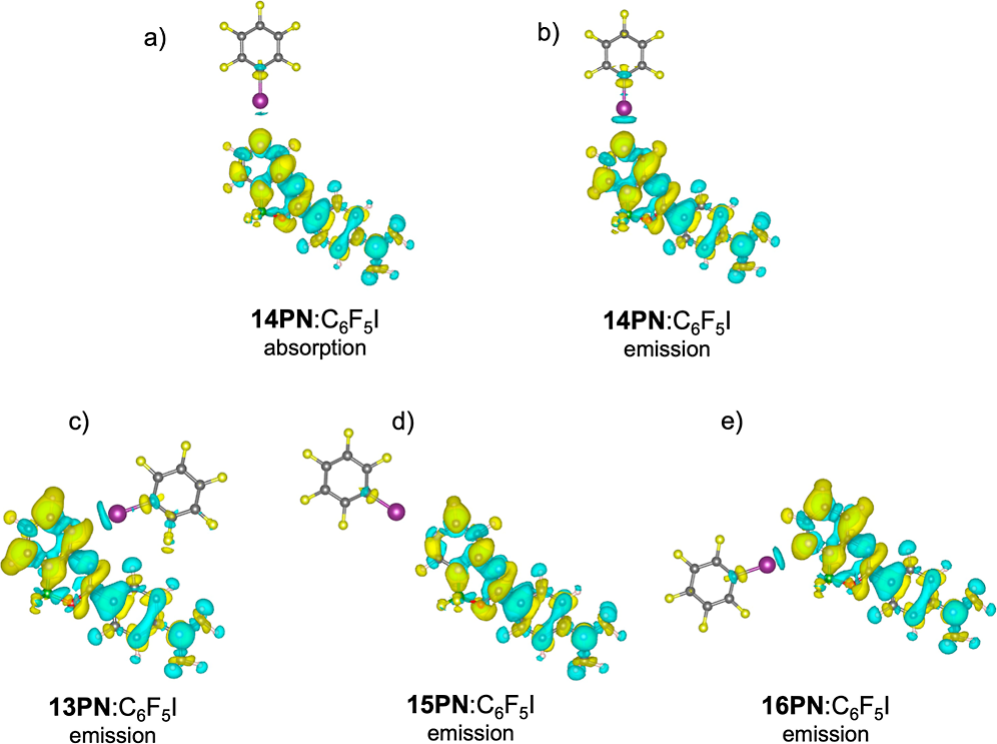
TD-DFT electron density difference between the excited and ground states of the (a) absorption process for the **14PN**:C_6_F_5_I complex, the (b) emission process for the **14PN**:C_6_F_5_I complex, the (c) emission process for the **13PN**:C_6_F_5_I complex, the (d) emission process for the **15PN**:C_6_F_5_I complex, and the (e) emission process for the **16PN**:C_6_F_5_I complex with interactions in the heterocyclic nitrogen atom.

For the absorption process in the **14PN**:C_6_F_5_I complex, the electron density difference plot (Figure 6a) reveals smaller changes in electron density in the halogen bond region in comparison with the emission process (Figure 6b), i.e., the plot indicates a significantly larger redistribution of electron density near the N···I bond for the emission process. This suggests that the N···I interaction has a weaker influence on absorption than that on emission. This observation aligns well with the relatively small shift in the absorption maximum wavelength (14 nm in TD-DFT and 15 nm in the experiment) compared with the larger shift in the fluorescence maximum wavelength (54 nm in TD-DFT and 61 nm in the experiment). These results indicate that the dyes exhibit weaker polarization in their ground state compared to that in their excited state.

Further analysis of electron density changes during the fluorescence process for other dyes in the series reveals that in the **15PN**:C_6_F_5_I complex, the electron density change near the N···I bond is significantly smaller than that in the other complexes (Figure 6d). This observation explains why the fluorescence wavelength shift induced by halogen bonding is much smaller for this dye upon solvent change compared to the others and why its fluorescence quantum yield remains relatively high. On the other hand, the larger electron density changes near the N···I bond in the **13PN**:C_6_F_5_I and **14PN**:C_6_F_5_I complexes correlate with the more substantial emission shifts upon solvent change observed experimentally (+53 nm and +61 nm, respectively). For the **16PN**:C_6_F_5_I complex, the electron density change near the N···I bond is significant but less pronounced compared to that in **13PN** and **14PN**. Consistently, the experimental XB-induced emission shift for **16PN** is smaller (+42 nm), lying between the larger shifts of **13PN** and **14PN** and the even lower shift of **15PN** (+33 nm).

The photophysical behavior of the **xPN** series interacting through a halogen bond at the imine nitrogen was also studied at the TD-DFT level (Table S2). In these complexes, the excited-state geometries exhibit a twist between the donor and acceptor sites, resulting in a perpendicular orientation between the −C_6_H_4_–NMe_2_ group and the electron-accepting site (Figure S50). This twisting occurs in three of the five complexes and results in a predicted zero oscillator strength for their emission processes. Consequently, these complexes are expected to show negligible emission intensity, potentially making their emission undetectable in experimental measurements.

The fluorescence quantum yields of the studied dyes span a broad range, reflecting the strong influence of the molecular structure and solvent interactions. These values range from a near-perfect yield of 1.00 for **15PN** in C_6_F_5_Cl to a low value of 0.02 (practically nonfluorescent) for **13PN** in C_6_F_5_I. Among the dyes, **PN** and **15PN** consistently exhibit higher Φ_f_, suggesting that interactions with the solvent environment do not significantly disrupt their radiative decay pathways. This confirms that **15PN** forms weaker halogen bonds compared with the other dyes in the series, thereby maintaining more efficient fluorescence. The emission quenching upon halogen bond interaction in the heterocyclic ring for **13PN**, **14PN**, and **16PN** is consistent with our previous observations.^42^

Lastly, the Stokes shift, which reflects the difference between absorption and emission wavelengths, further highlights the effect of both the solvent and the position of the nitrogen atom involved in the halogen bond interaction. The values, summarized in Table 3, reveal smaller values for **PN** and **15PN**, which may indicate weaker intramolecular CT in these dyes compared to others in the series. The largest Stokes shifts for each dye are consistently observed in C_6_F_5_I, again suggesting stronger halogen bonding interactions with the heterocyclic nitrogen atoms from the dyes.

## 3 Conclusions

This study thoroughly explored the influence of halogen bonding on the photophysical properties of a series of heterocyclic dyes, focusing on two nitrogen acceptor sites and their interactions with halogenated solvents. NMR titrations revealed weak halogen bonding interactions for all dyes. However, specific nitrogen sites, such as heterocyclic nitrogen, exhibited notable deshielding effects, confirming halogen bonding in the ground state, further supported by quantum chemical calculations. The position of nitrogen within the heterocyclic ring and the halogen bond interactions with the solvent significantly influenced the absorption and emission wavelengths. The absorption maxima showed a predictable red-shift when switching from C_6_F_6_ to halogen-bonding C_6_F_5_I.

Emission spectra displayed larger shifts in C_6_F_5_I compared to C_6_F_6_, a behavior not observed in C_6_F_5_Cl and C_6_F_5_Br. Gibbs energy calculations confirmed that only complexes formed with C_6_F_5_I were thermodynamically favorable under experimental conditions, explaining the more pronounced red-shifts caused by enhancing electron-accepting properties of the heterocycle. In parallel, lower fluorescence quantum yields are observed in a halogen bonding environment.

Electron density redistribution near the halogen bond during emission was used to explain the larger shifts observed in the emission spectra relative to the absorption spectra. Additionally, electron density redistribution plots were used to explain the varying sensitivities to halogen bonding exhibited by different dyes. Dyes like **13PN** and **14PN**, with stronger halogen bonding interactions, showed significant red-shifts and lower fluorescence quantum yields, while dyes like **15PN**, with weaker interactions, maintained higher fluorescence yields and smaller shifts, suggesting that the nitrogen at the 5-position is not suitable for halogen bond sensing in solution.

## 4 Experimental Section

### 4.1 Synthesis

The synthesis of **PN** and **13–16PN** dyes was performed in the same way as the one described in one of our previous publications.^41,47,48^ The model molecules carrying the *t*-Bu group were obtained as described before^49^ with the use of pivaloyl chloride instead of acetyl chloride. **DMAP** was a commercial compound used after recrystallization from ethanol. The purification of dyes **PN**-**16PN** and model compounds **PN**^***tBu***^-**16PN**^***tBu***^ was performed by flash chromatography using a Buchi C-815 flash chromatography system equipped with absorption and ELSD detectors.

### 4.2 Characterization Data

All spectra are collected in the Supporting Information.

4-(1,1-Difluoro-1*H*-1λ^4^,9λ^4^-pyrido[1,2-*c*][1,3,5,2]oxadiazaborinin-3-yl)-*N*,*N*-dimethylaniline (**PN**). Purification by flash chromatography (hexane/ethyl acetate in the gradient mode, SiO_2_). Yellow amorphous solid. Yield 0.45 g, 62.6%, mp 172–173 °C. ^1^H (400 MHz, CDCl_3_, TMS) 8.24 (m, 3H), 7.96 (ddd, *J* = 1.8, 7.4, 9.0 Hz, 1H), 7.45 (d, *J* = 8.6 Hz, 1H), 7.22 (ddd, *J* = 1.1, 6.0, 6.6 Hz, 1H), 6.69 (d, *J* = 9.0 Hz, 2H), 3.08 (s, 6H), ^13^C{^1^H} (100 MHz, CDCl_3_, TMS) 166.1, 154.9, 153.9, 143.0, 138.2, 131.9, 122.9, 118.9, 118.4, 111.0, 40.1, ^11^B (128 Hz, CDCl_3_) 0.40 (t, *J* = 14.3 Hz), ^19^F (376 Hz, CDCl_3_) −140.6 (m). HRMS (ES+, TOF) *m*/*z*: [M + H]^+^ calcd for C_14_H_15_BF_2_N_3_O, 290.1276; found, 290.1277.

4-(1,1-Difluoro-1*H*-1λ^4^,9λ^4^-pyrimido[1,2-*c*][1,3,5,2]oxadiazaborinin-3-yl)-*N*,*N*-dimethylaniline (**13PN**). Purification by flash chromatography (hexane/ethyl acetate in the gradient mode, SiO_2_). Yellow-orange crystalline solid. Yield 0.52 g, 66.8%, mp 201–203 °C. ^1^H (400 MHz, CDCl_3_, TMS) 9.06 (dd, *J* = 2.6, 4.5 Hz, 1H), 8.52 (d broad, 1H), 8.35 (d, *J* = 9.2 Hz, 2H), 7.25 (dd overlapped with solvents, 1H), 6.83 (d, *J* = 9.6 Hz, 2H), 3.13 (s, 6H), ^13^C{^1^H} (100 MHz, CDCl_3_, TMS) 169.7, 165.7, 158.2, 153.7, 147.5, 132.9, 115.1, 112.2, 40.9. ^11^B (128 Hz, CDCl_3_) 0.66 (t, *J* = 13.4 Hz), ^19^F (376 Hz, CDCl_3_) −139.6 (m). HRMS (ES+, TOF) *m*/*z*: [M + Na]^+^ calcd for C_13_H_13_BF_2_N_4_ONa, 313.1048; found, 313.1052.

4-(1,1-Difluoro-1*H*-1λ^4^,9λ^4^-pyrazino[1,2-*c*][1,3,5,2]oxadiazaborinin-3-yl)-*N*,*N*-dimethylaniline (**14PN**). Purification by flash chromatography (hexane/ethyl acetate in the gradient mode, SiO_2_). Orange crystalline solid. Yield 0.0.45 g, 83.5%, mp 208–209 °C. ^1^H (400 MHz, CDCl_3_, TMS) 8.83 (d, *J* = 1.3 Hz, 1H), 8.41 (d *J* = 3.7 Hz, 1H), 8.23 (d, *J* = 9.1 Hz, 2H), 8.00 (d, *J* = 3.7 Hz, 1H), 6.69 (d, *J* = 9.1 Hz 2H), 3.10 (s, 6H), ^13^C{^1^H} (100 MHz, CDCl_3_, TMS) 168.9, 153.9, 148.8, 148.4, 138.2, 132.5, 128.7, 111.8, 40.6. ^11^B (128 Hz, CDCl_3_) 0.11 (t, *J* = 12.8 Hz), ^19^F (376 Hz, CDCl_3_) −139.5 (m). HRMS (ES+, TOF) *m*/*z*: [M + Na]^+^ calcd for C_13_H_13_BF_2_N_4_ONa, 313.1048; found, 313.1050.

4-(1,1-Difluoro-1*H*-1λ^4^,9λ^4^-pyrimido[1,6-*c*][1,3,5,2]oxadiazaborinin-3-yl)-*N*,*N*-dimethylaniline (**15PN**). Purification by flash chromatography (hexane/ethyl acetate in the gradient mode, SiO_2_). Yellow amorphous solid. Yield 0.48 g, 57.3%, mp 234–236 °C. ^1^H (400 MHz, CDCl_3_, TMS) 8.91 (s, 1H), 8.67 (d, *J* = 5.9 Hz, 1H), 8.33 (d, *J* = 9.1 Hz, 2H), 7.42 (d, *J* = 5.9 Hz, 1H), 6.78 (d, *J* = 9.1 Hz, 2H), 3.14 (s, 6H), ^13^C{^1^H} (100 MHz, CDCl_3_, TMS)169.2, 159.5, 157.7, 154.6, 150.2, 133.3, 117.5, 117.0, 111.8, 40.5. ^11^B (128 Hz, CDCl_3_) 0.03 (t, *J* = 13.2 Hz), ^19^F (376 Hz, CDCl_3_) −139.9 (m) HRMS (ES+, TOF) *m*/*z*: [M + Na]^+^ calcd for C_13_H_13_BF_2_N_4_ONa, 313.1048; found, 313.1052.

4-(1,1-Difluoro-1*H*-1λ^4^,9λ^4^-pyridazino[1,6-*c*][1,3,5,2]oxadiazaborinin-3-yl)-*N*,*N*-dimethylaniline (**16PN**). Purification by flash chromatography (ethyl acetate, SiO_2_). Orange crystalline solid. Yield 0.34 g, 35.5%, mp 255–256 °C. ^1^H (400 MHz, CDCl_3_, TMS) 8.77 (dd, *J* = 1.7, 4.3 Hz, 1H), 8.26 (d, *J* = 9.2 Hz, 2H), 7.70 (dd, *J* = 4.3, 9.0 Hz, 1H), 7.64 (dd, *J* = 1.7, 9.0 Hz, 1H), 6.70 (d, *J* = 9.2 Hz, 2H), 3.14 (s, 6H), ^13^C{^1^H} (100 MHz, CDCl_3_, TMS) 167.7, 156.2, 154.2, 147.1, 133.3, 133.1, 129.9, 117.9, 112.1, 40.7. ^11^B (128 Hz, CDCl_3_) 0.23 (t, *J* = 9.8 Hz), ^19^F (376 Hz, CDCl_3_) −140.0 (m) HRMS (ES+, TOF) *m*/*z*: [M + Na]^+^ calcd for C_13_H_13_BF_2_N_4_ONa, 313.1048; found, 313.1052.

3-(*tert*-Butyl)-1,1-difluoro-1*H*-1λ^4^,9λ^4^-pyrido[1,2-*c*][1,3,5,2]oxadiazaborinine (**PN**^***tBu***^). Purification was by flash chromatography (hexane/dichloromethane in the gradient mode, SiO_2_). White crystalline solid. Yield 0.90 g, 74.5%, mp 97.9–98.5 °C. ^1^H (400 MHz, CDCl_3_, TMS) 8.28 (d broad, *J* = 5.2 Hz, 1H), 8.04 (ddd, *J* = 1.8, 7.3, 8.4 Hz, 1H), 7.40 (d, *J* = 8.4 Hz, 1H), 7.36 (ddd, *J* = 1.2, 6.0, 7.3 Hz, 1H), 1.32 (s, 9H), ^13^C{^1^H} (100 MHz, CDCl_3_, TMS) 179.5, 154.4, 143.7, 138.3, 123.2, 120.6, 38.9, 27.6. ^11^B (128 Hz, CDCl_3_) 0.24 (t, *J* = 13.9 Hz), ^19^F (376 Hz, CDCl_3_) −140.1 (m). HRMS (ES+, TOF) *m*/*z*: [M + H]^+^ calcd for C_10_H_14_BF_2_N_2_O, 227.1167; found, 227.1169.

3-(*tert*-Butyl)-1,1-difluoro-1*H*-1λ^4^,9λ^4^-pyrimido[1,2-*c*][1,3,5,2]oxadiazaborinine (**13PN**^***tBu***^). Purification by flash chromatography (hexane/dichloromethane in the gradient mode, SiO_2_). White crystalline solid. Yield 0.98 g, 64.5%, mp 175–177 °C. ^1^H (400 MHz, CDCl_3_, TMS) 9.09 (m, 1H), 8.55 (d, *J* = 3.8 Hz, 1H), 7.42 (dd, *J* = 4.6, 6.0 Hz, 1H), 1.29 (s, 9H), ^13^C{^1^H} (176 MHz, CDCl_3_, TMS) 184.8, 166.1, 157.9, 147.8, 117.2, 39.5, 27.5. ^11^B (224 Hz, CDCl_3_) 0.87 (t, *J* = 13.1 Hz), ^19^F (376 Hz, CDCl_3_) −138.5. HRMS (ES+, TOF) *m*/*z*: [M + H]^+^ calcd for C_9_H_13_BF_2_N_3_O, 228.1120; found, 228.1123.

3-(*tert*-Butyl)-1,1-difluoro-1*H*-1λ^4^,9λ^4^-pyrazino[1,2-*c*][1,3,5,2]oxadiazaborinine (**14PN**^***tBu***^). Purification by flash chromatography (hexane/dichloromethane in the gradient mode, SiO_2_). Light-brown/orange amorphous solid. Yield 0.94 g, 67.5%, mp 99–100 °C. ^1^H (400 MHz, CDCl_3_, TMS) 8.87 (s, 1H), 8.63 (d, *J* = 3.5 Hz, 1H), 8.09 (d, *J* = 3.5 Hz, 1H), 1.33 (s, 9H), ^13^C{^1^H} (100 MHz, CDCl_3_, TMS) 183.7, 148.3, 148.1, 140.6, 128.9, 39.5, 27.5. ^11^B (128 Hz, CDCl_3_) −0.03 (t, *J* = 12.3 Hz), ^19^F (376 Hz, CDCl_3_) 138.4 (m). HRMS (ES+, TOF) *m*/*z*: [M + H]^+^ calcd for C_9_H_13_BF_2_N_3_O, 228.1120; found, 228.1122.

3-(*tert*-Butyl)-1,1-difluoro-1*H*-1λ^4^,9λ^4^-pyrimido[1,6-*c*][1,3,5,2]oxadiazaborinine (**15PN**^***tBu***^). Purification by flash chromatography (hexane/dichloromethane in the gradient mode, SiO_2_). White crystalline solid. Yield 1.10 g, 62.0%, mp 55–56.5 °C. ^1^H (400 MHz, CDCl_3_, TMS) 8.96 (s, 1H), 8.79 (d, *J* = 5.8 Hz, 1H), 7.27 (d, *J* = 5.8 Hz, 1H), 1.31 (s, 9H), ^13^C{^1^H} (100 MHz, CDCl_3_, TMS) 185.1, 160.8, 158.6, 150.2, 117.9, 39.7, 27.5. ^11^B (128 Hz, CDCl_3_) −0.06 (t, *J* = 12.9 Hz), ^19^F (376 Hz, CDCl_3_) −137.3 (m). HRMS (ES+, TOF) *m*/*z*: [M + H]^+^ calcd for C_9_H_13_BF_2_N_3_O, 228.1120; found, 228.1122.

3-(*tert*-Butyl)-1,1-difluoro-1*H*-1λ^4^,9λ^4^-pyridazino[1,6-*c*][1,3,5,2]oxadiazaborinine (**16PN**^***tBu***^). Purification by flash chromatography (hexane/dichloromethane in the gradient mode, SiO_2_). White amorphous solid. Yield 0.89 g, 66.9%, mp 117–119 °C. ^1^H (400 MHz, CDCl_3_, TMS) 8.92 (dd, *J* = 1.6, 4.4 Hz, 1H), 7.92 (ddd, *J* = 0.7, 4.4, 8.9 Hz, 1H), 7.68 (dd, *J* = 1.6, 8.9 Hz, 1H), 1.31 (s, 9H), ^13^C{^1^H} (176 MHz, CDCl_3_, TMS) 182.9, 156.9, 148.5, 134.4, 130.8, 39.3, 27.6. ^11^B (128 Hz, CDCl_3_) 0.10 (t, *J* = 9.6 Hz), ^19^F (376 Hz, CDCl_3_) −138.4 (m). HRMS (ES+, TOF) *m*/*z*: [M + Na]^+^ calcd for C_9_H_12_BF_2_N_3_ONa, 250.0939; found 250.0941.

### 4.3 Measurements

All compounds were dried in a desiccator for at least 5 days, and solvents were distilled before their use. The same distilled solvents were used for the absorption and luminescence experiments. NMR spectroscopy, including titrations (starting concentration of the host molecules varied between 5.8×10^–7^ and 6.5×10^–7^ mol/L) and two-component solutions (fluorinated solvent and the dye), was employed to elucidate halogen bonding in the studied dyes in their ground state. Figures S41–S44 illustrate the changes in ^1^H NMR chemical shifts for each proton and each dye in titration experiments. The data from titrations were analyzed using Bindfit available at http://supramolecular.org/.^50,51^ Due to its low solubility, **16PN** was excluded from the dye titration experiments. The ^15^N NMR data (^1^H–^15^N HMBC) used as a reference were recorded in a mixture of solvents C_6_F_6_/C_6_D_6_ (9/1 v/v, *id est.* 0.54 mL of C_6_F_6_ with 0.06 mL of C_6_D_6_ added to lock the spectrometer). Similar ^15^N NMR data were obtained in C_6_F_5_I to evaluate the difference in the ^15^N chemical shifts and measure the effects of XB bonding (vide infra). The *two-component* approach maximized specific interactions, enabling the collection of reliable ^15^N NMR data. The absorption spectra (Figure S46) were recorded within 1 h after the preparation of the solution with the use of a Shimadzu UV-1900 spectrophotometer in quartz cuvette (path 1 cm), while for emission studies (Figure S47), the FS5 (Edinburgh Instruments) spectrofluorimeter was used equipped with the thermostated cuvette holder, integrating sphere and TCSPC setup for fluorescence lifetimes (excitation with a laser at 374 or 447 nm, depending on the overlap of the excitation light with the absorption spectrum of the dye and solvent). The absolute Φ_f_ was determined by integration of the emission band (resolution: 0.1 nm, dwell time 0.2 s, 3 repeats), while the pure solvent (scattered light peak) of the same type was used as a reference in the same, previously clean and dried cuvette.

### 4.4 Computational Details

Quantum chemical computations were employed as a valuable tool to elucidate the mechanisms underlying the experimental observations. Geometry optimizations and Hessian evaluations in both the ground and ES were carried out using the MN15 hybrid density functional^52^ in combination with the aug-cc-pVDZ basis set^53,54^ using the Gaussian 16 software package.^55^ For bromine and iodine atoms, the aug-cc-pVDZ-PP basis set^56^ was used alongside the corresponding pseudopotential to account for scalar relativistic effects. TD-DFT at the MN15/aug-ccpVDZ(PP) level was utilized to simulate the absorption and emission spectra. Additionally, interaction energies were calculated using the SCS-MP2 method and the aug-cc-pVDZ(PP) basis set, with calculations performed using the MOLPRO program.^57^ The supermolecular approach was applied to calculate the Gibbs energy change (Δ*G*) taking into account the correction when moving from a standard gas state that uses a pressure of 1 atm to a standard solvent state that uses a concentration of *X* M (where *X* is the molarity concentration of reagents in agreement with the experimental data).^58^ The density difference plots for vertical absorption and emission processes were prepared based on the data generated using the *cubegen* and *cubman* utilities from the Gaussian 16 suite.^55^

## Data Availability

The data underlying this study are available in the published article and its online Supporting Information.

## Supporting Information Available

Characterization data for all compounds (NMR, titration charts, CIS values, and spectra) and additional computational data (geometries, calculated photophysical parameters, and energies of interaction) (PDF)
