# Supplementary material for: Isoelectronic Push–Pull Fluorescent Difluoroborates: Halogen Bonding and Photophysical Properties
Source: J Org Chem. 2025 Feb 11;90(7):2790–9. doi: 10.1021/acs.joc.4c03077 (PMC11852195; doi:10.1021/acs.joc.4c03077)
Supplement: Supplementary file 1 — jo4c03077_si_001.pdf [file jo4c03077_si_001.pdf]

Supporting Information: Isoelectronic Push–Pull Fluorescent Difluoroborates: Halogen Bonding and Photophysical Properties

Alex Iglesias-Reguant,[†] Izabela Barańska,[†] Damian Plażuk,[‡] Robert Zaleśny,[¶] Josep Maria Luis,[*,§] and Borys Ośmiałowski[*,†]

†*Faculty of Chemistry, Nicolaus Copernicus University, Gagarina Street 7, 87-100 Toruń, Poland*

‡*Laboratory of Molecular Spectroscopy, Department of Organic Chemistry, Faculty of Chemistry, University of Lodz, Tamka 12, 91-403 Lodz, Poland*

¶*Faculty of Chemistry, Wroclaw University of Science and Technology, Wybrzeże Wyspiańskiego 27, 50-370 Wrocław, Poland*

§*Institute of Computational Chemistry and Catalysis and Department of Chemistry, University of Girona, Campus de Montilivi, Girona, Catalonia, Spain*

E-mail: josepm.luis@udg.edu; borys.osmialowski@umk.pl

Contents

1 General procedures and techniques **S3**

1.1 Synthesis and structure confirmation S3

1.2 Photophysical measurements S3

1.3 Quantum-chemical calculations S4

2 NMR spectra **S5**

3 Titration charts and CIS values **S25**

4 Normalized absorption and fluorescence spectra **S28**

5 Geometries **S29**

6 Calculated photophysical data **S32**

7 Crucial interatomic distances and angled, and interaction energies **S35**

8 Additional figures **S37**

9 Cartesian coordinates **S37**

1 General procedures and techniques

1.1 Synthesis and structure confirmation

All reactions were guided in anhydrous solvents using aminoheterocycles and ethyl *N*,*N*-dimethylaminobenzoate as substrates, and NaH as a base. The obtained amides were purified by recrystallization from ethanol, and dried before reaction with BF_3 etherate. Since the BF_3 etherate creates acidic fumes when exposed to air and moisture, it is advised to work under well-ventilated fume-hood. The last reaction was guided at r.t. and monitored by TLC. The final products were purified by flash chromatography (Buchi, C-815), and the structure was confirmed by multi-nuclei NMR, HRMS, and melting point measurements. NMR spectra (structure conformation and titrations) were recorded at 400 MHz (or 700 MHz) spectrometers (Bruker, noted in the main text), while HRMS were performed using a mass spectrometer (Waters, Synapt G2-S) equipped with the electrospray ion source and quadrupole-time-of-flight mass analyzer. The results of NMR titrations were analyzed with the use of the Bindfit program available at http://supramolecular.org/.

1.2 Photophysical measurements

Photophysical measurements were guided in freshly distilled solvents in a quartz cuvette (1 cm path). In the absorption regime, the spectra of the solvent were recorded (Shimadzu, UV-1900) separately to check if there is an overlap in the absorption of the spectrum of solvent with studied dyes. That was the case for C_6F_5I, thus the spectra are cut at *ca.* 390 nm. The emission spectra (Edinburgh Instruments, FS5) were recorded at 20°C (thermostated holder) in specified solvents. The excitation spectra were overlapped with the absorption. The fluorescence quantum yield (Edinburgh Instruments, FS5) was determined by absolute methods with the use of an integrating sphere, while the fluorescence decay (Edinburgh Instruments, FS5) in time was recorded using a time-correlated single photon counting technique.

1.3 Quantum-chemical calculations

Quantum chemical computations were performed to provide insight into the experimental findings and the underlying molecular mechanisms. Geometry optimizations and frequency calculations in the ground and excited states were carried out using the MN15 hybrid density functional combined with the aug-cc-pVDZ basis set, as implemented in the Gaussian 16 software package. For bromine and iodine atoms, the aug-cc-pVDZ-PP basis set with the corresponding pseudopotentials was employed to account for scalar relativistic effects. Vertical excitation and emission energies were calculated using time-dependent density functional theory (TD-DFT) at the MN15/aug-cc-pVDZ(PP) level of theory. Interaction energies were evaluated using the SCS-MP2 method the SCS-MP2 method and the aug-cc-pVDZ(PP) basis set, with calculations performed using the MOLPRO program. Thermodynamic parameters, including the Gibbs free energy change (ΔG), were computed using the supermolecular approach. Corrections were applied to transition from the standard gas state at 1 atm to the experimental standard state in solution, based on the molarity of reagents. Density difference plots for vertical absorption and emission transitions were generated with the *cubegen* and *cubman* utilities in Gaussian 16.

2 NMR spectra

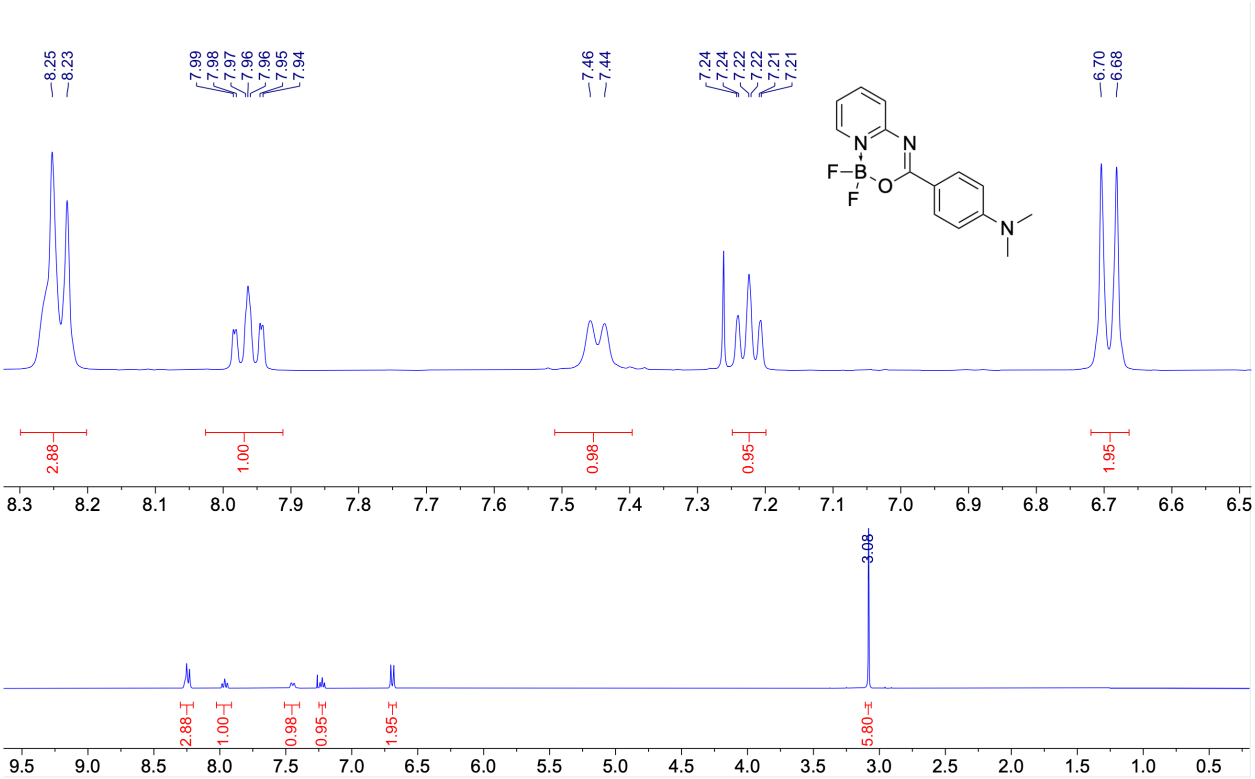

Figure S1: ^{1}H NMR spectrum (in $CDCl_3$, 400 MHz) of **PN**

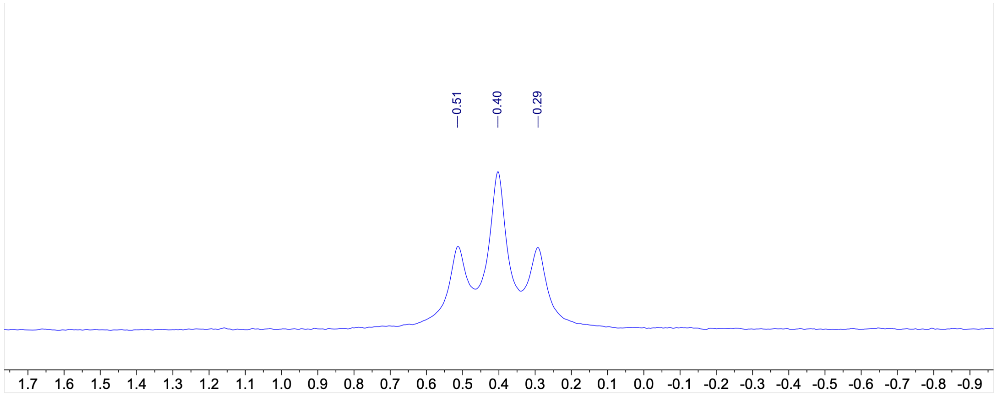

Figure S2: ^{11}B NMR spectrum (in $CDCl_3$, 128 MHz) of **PN**

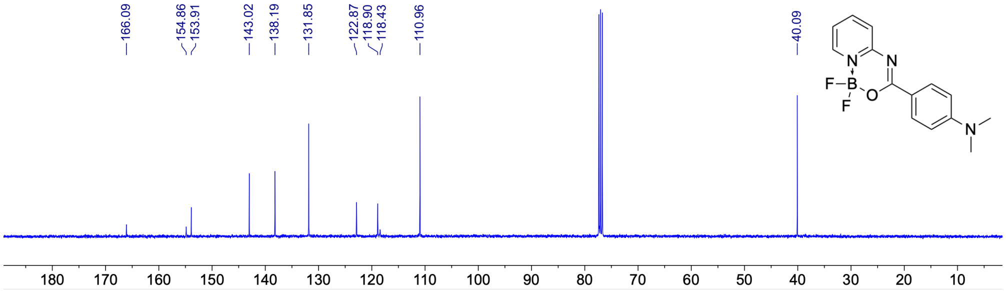

Figure S3: ^{13}C{^{1}H} NMR spectrum (in $CDCl_3$, 100 MHz) of **PN**

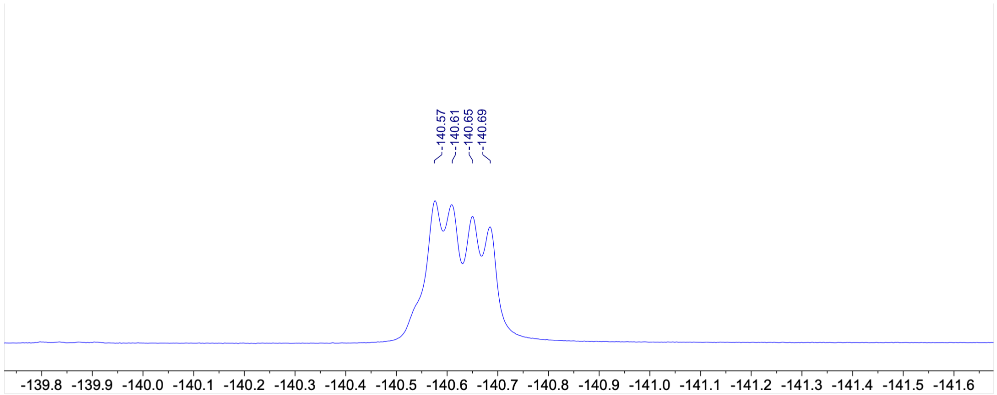

Figure S4: ^{19}F NMR spectrum (in $CDCl_3$, 376 MHz) of **PN**

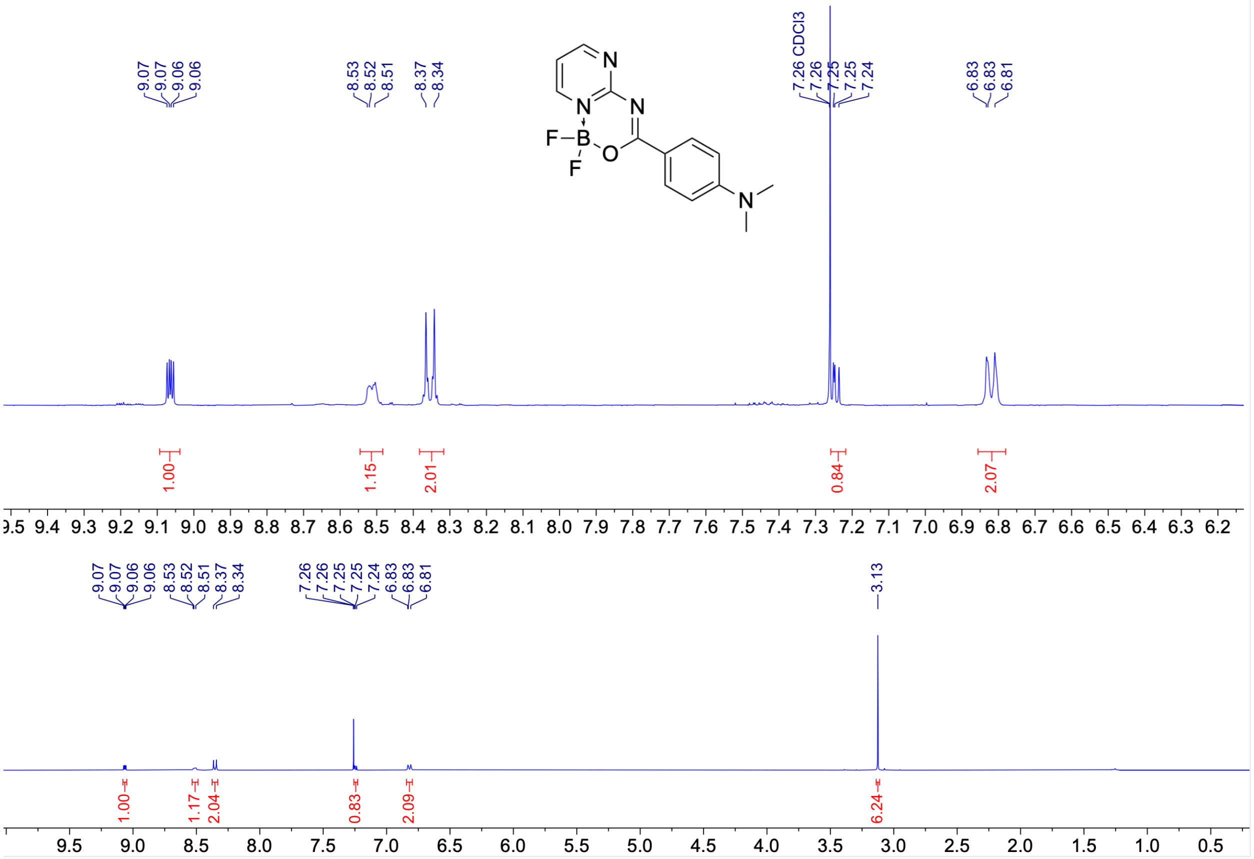

Figure S5: ^{1}H NMR spectrum (in $CDCl_3$, 400 MHz) of **13PN**

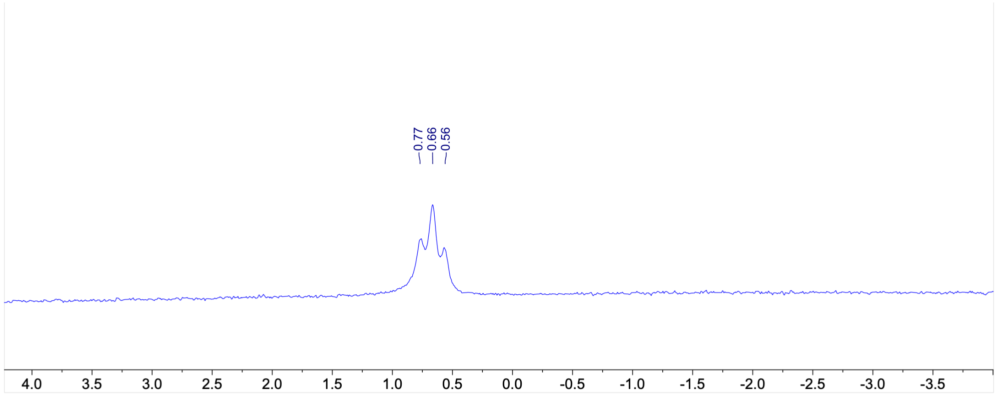

Figure S6: ^{11}B NMR spectrum (in $CDCl_3$, 128 MHz) of **13PN**

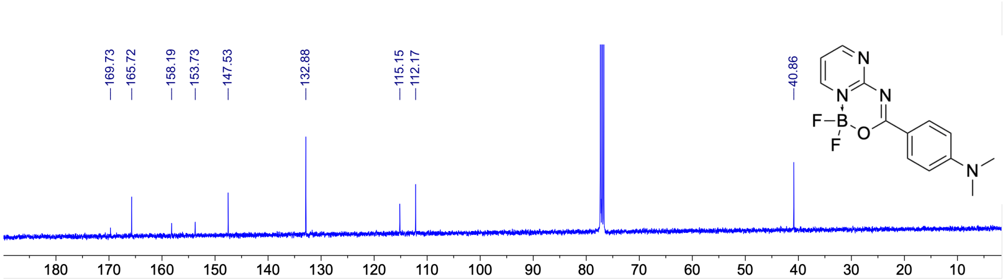

Figure S7: $^{13}C\{^{1}H\}$ NMR spectrum (in $CDCl_3$, 100 MHz) of **13PN**

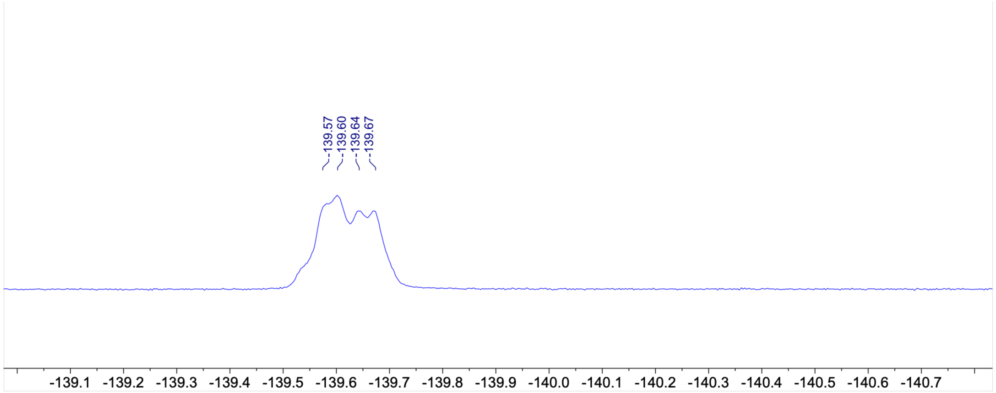

Figure S8: ^{19}F NMR spectrum (in $CDCl_3$, 376 MHz) of **13PN**

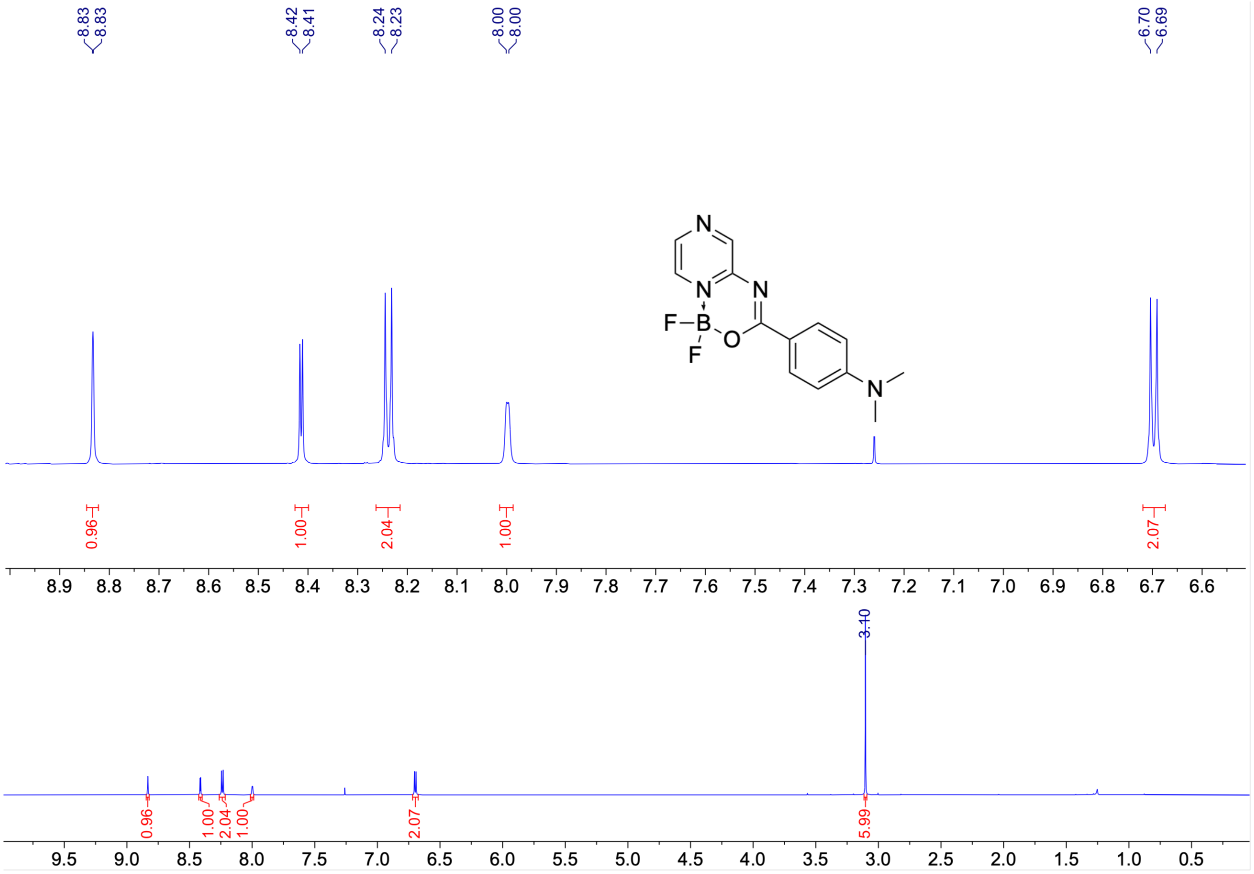

Figure S9: ^{1}H NMR spectrum (in $CDCl_3$, 700 MHz) of **14PN**

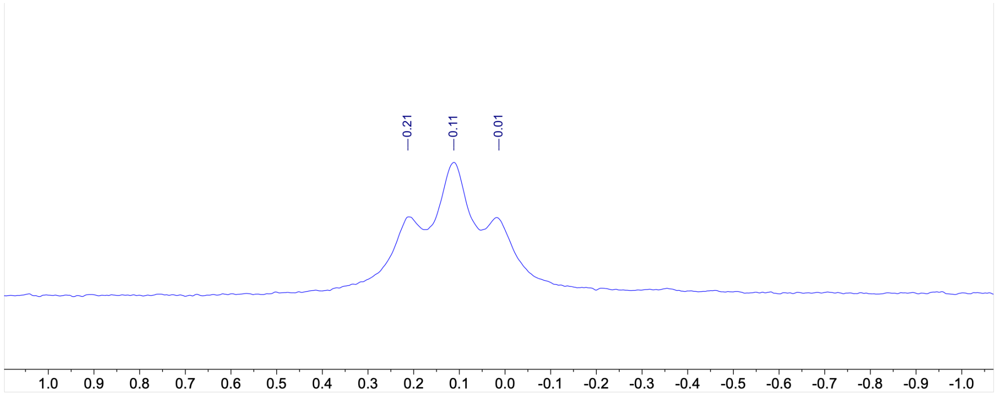

Figure S10: ^{11}B NMR spectrum (in $CDCl_3$, 128 MHz) of **14PN**

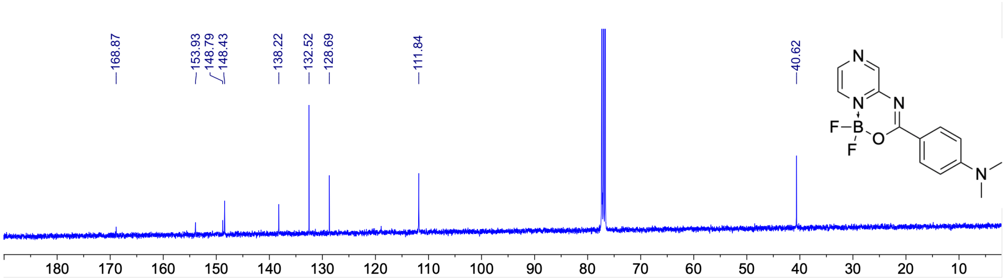

Figure S11: $^{13}C\{^{1}H\}$ NMR spectrum (in $CDCl_3$, 100 MHz) of **14PN**

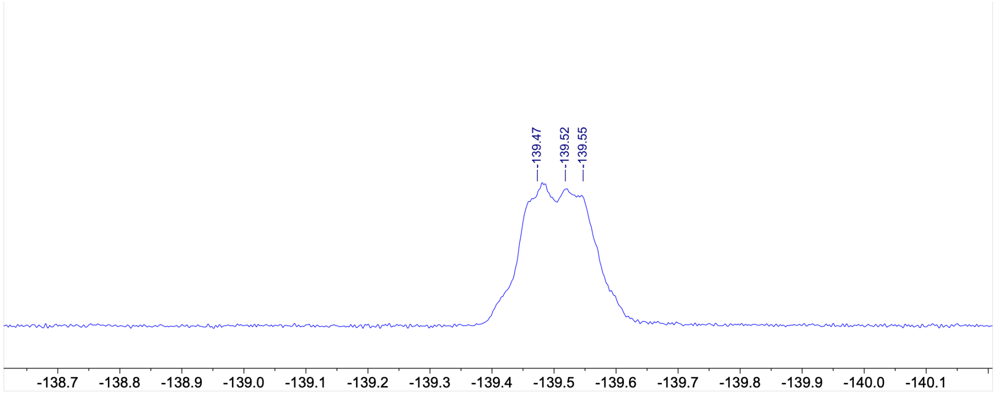

Figure S12: ^{19}F NMR spectrum (in $CDCl_3$, 376 MHz) of **14PN**

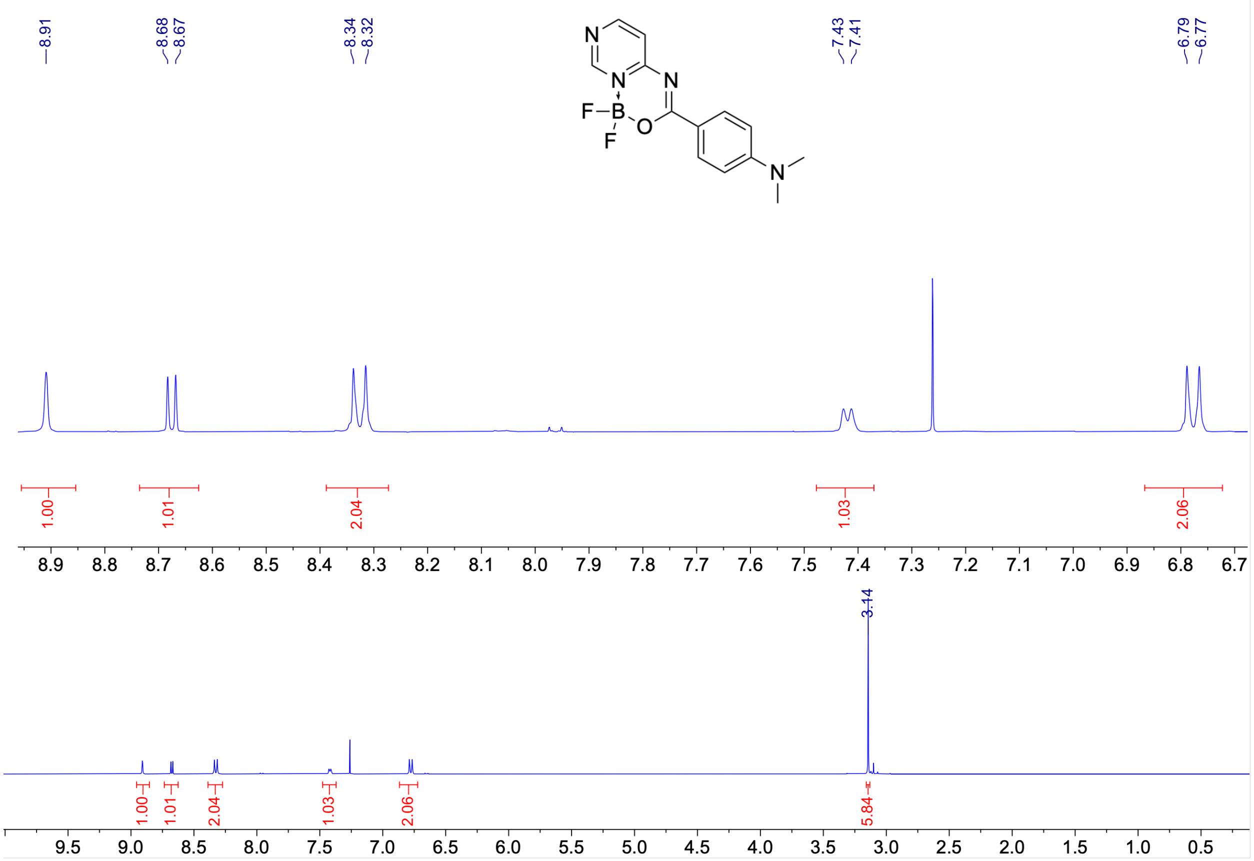

Figure S13: ^{1}H NMR spectrum (in $CDCl_3$, 400 MHz) of **15PN**

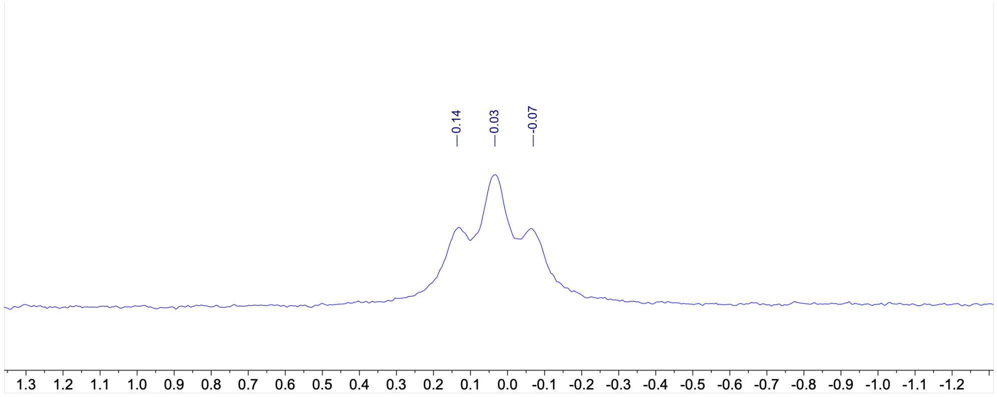

Figure S14: ^{11}B NMR spectrum (in $CDCl_3$, 128 MHz) of **15PN**

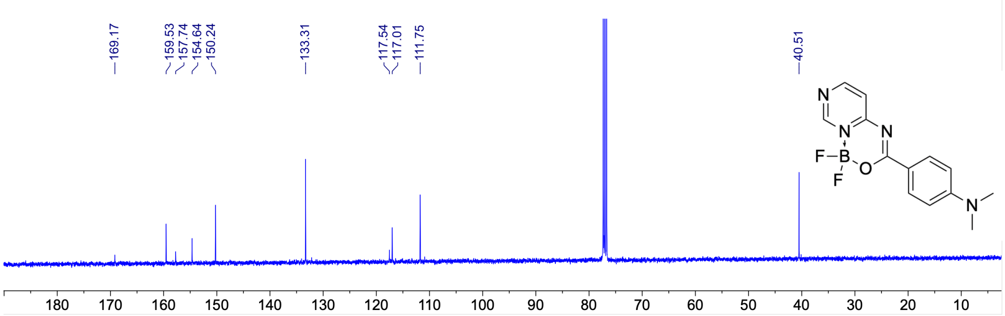

Figure S15: $^{13}C\{^{1}H\}$ NMR spectrum (in $CDCl_3$, 100 MHz) of **15PN**

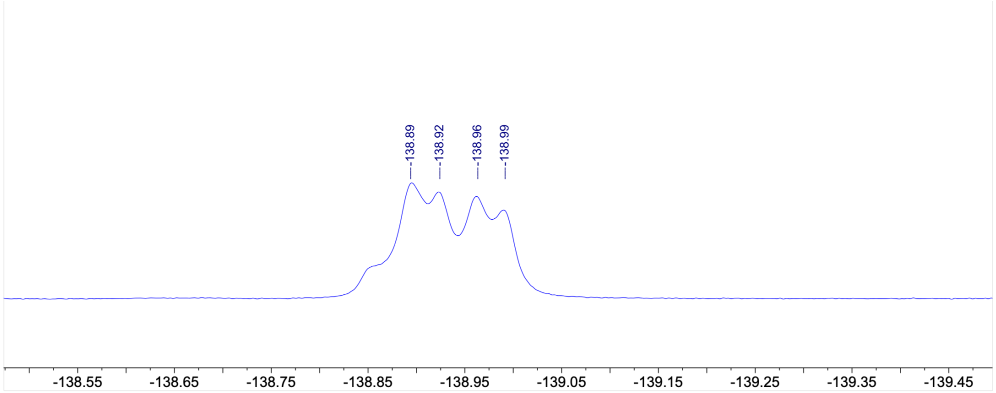

Figure S16: ^{19}F NMR spectrum (in $CDCl_3$, 376 MHz) of **15PN**

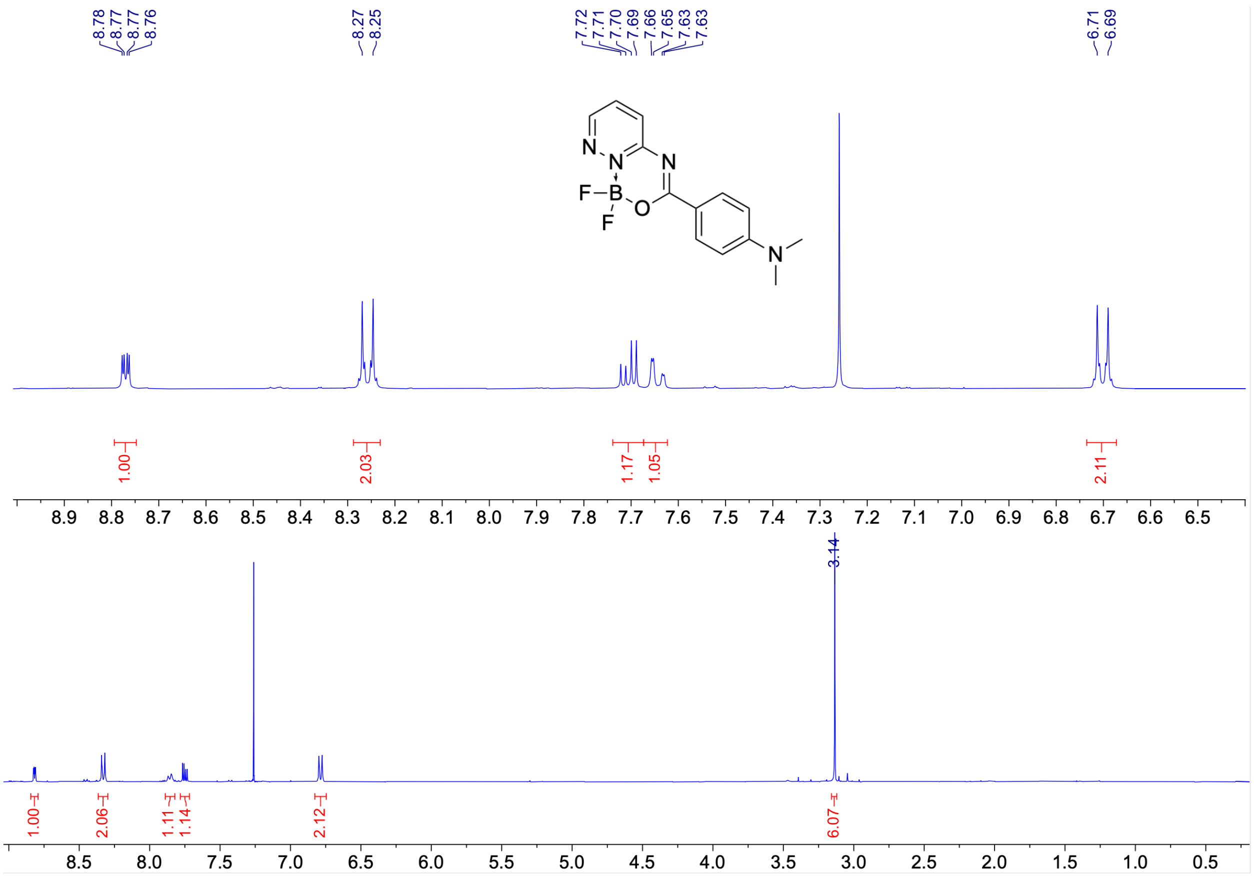

Figure S17: ^{1}H NMR spectrum (in $CDCl_3$, 400 MHz) of **16PN**

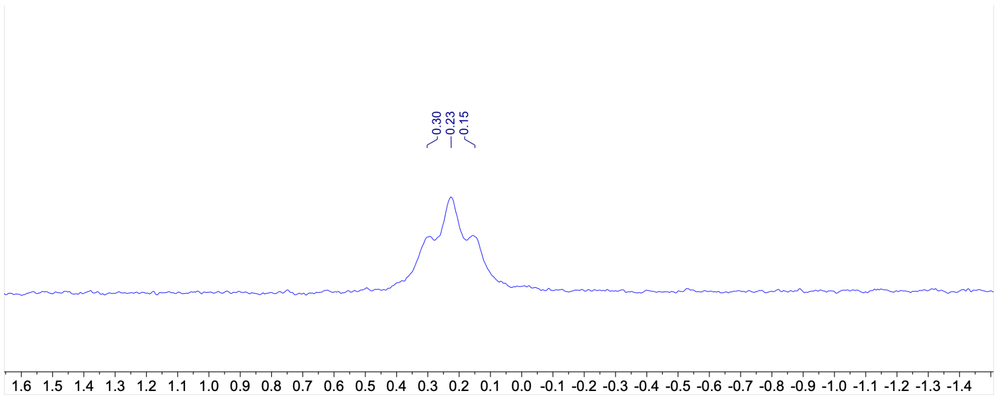

Figure S18: ^{11}B NMR spectrum (in $CDCl_3$, 128 MHz) of **16PN**

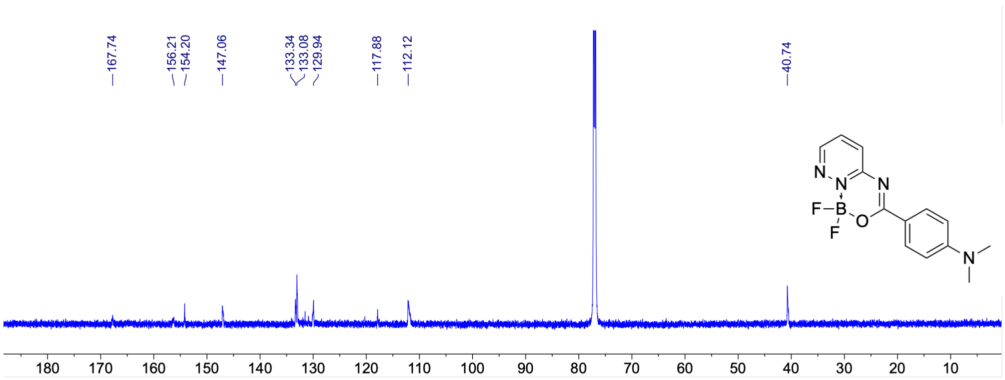

Figure S19: $^{13}C\{^{1}H\}$ NMR spectrum (in $CDCl_3$, 176 MHz) of **16PN**

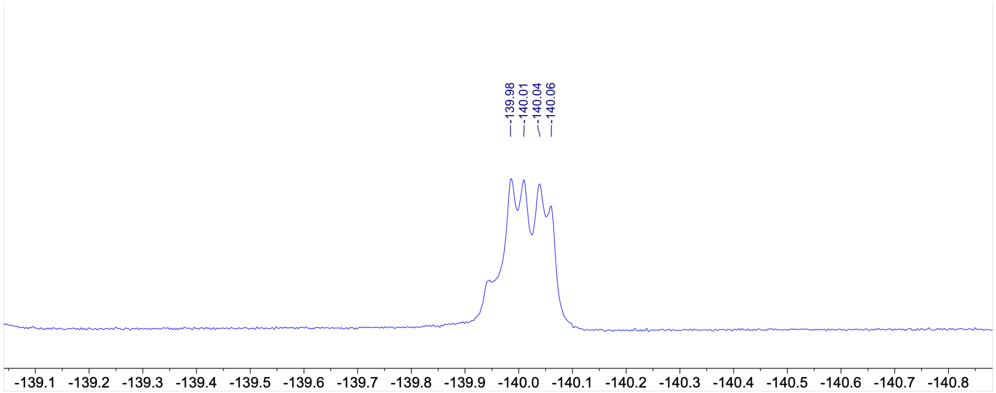

Figure S20: ^{19}F NMR spectrum (in $CDCl_3$, 376 MHz) of **16PN**

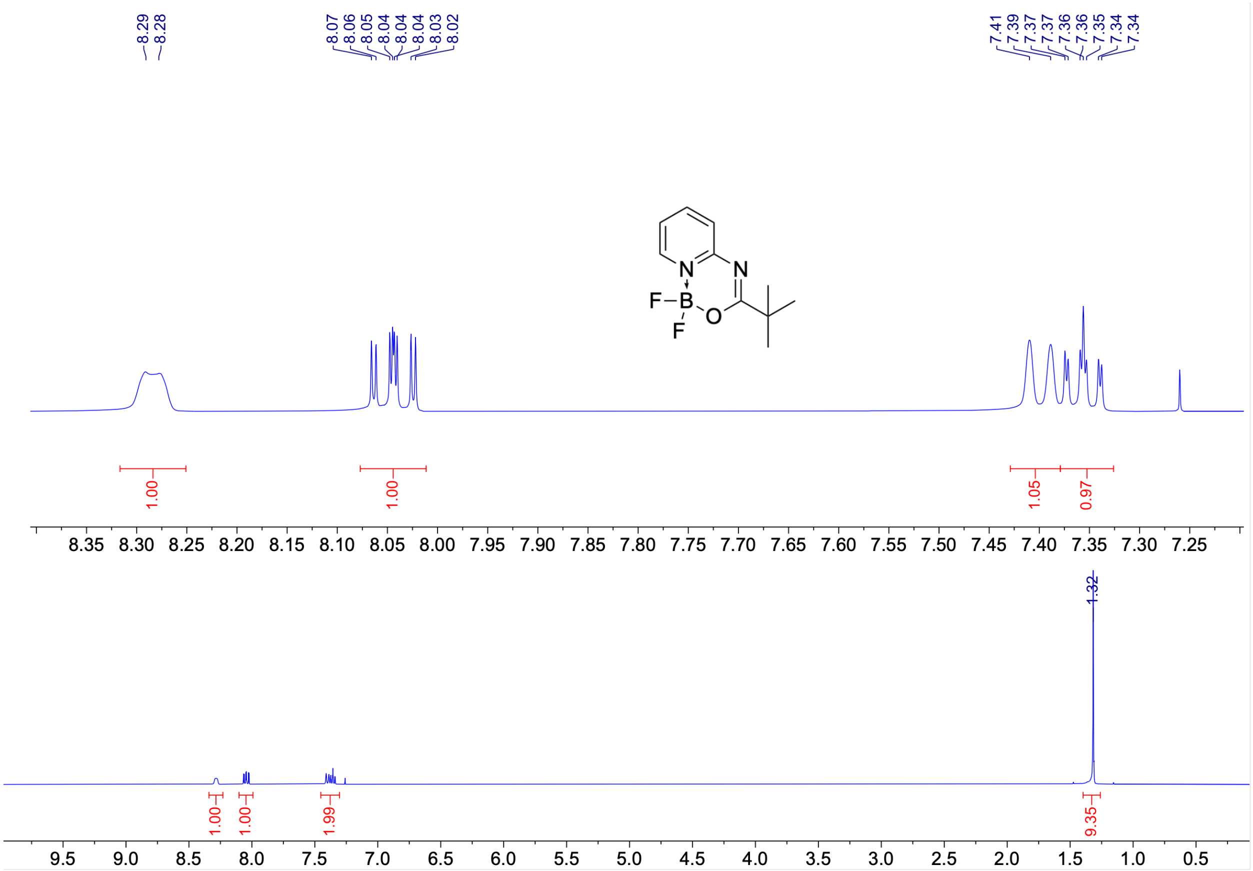

Figure S21: ^{1}H NMR spectrum (in $CDCl_3$, 400 MHz) of $\mathbf{PN}^{tBu}$

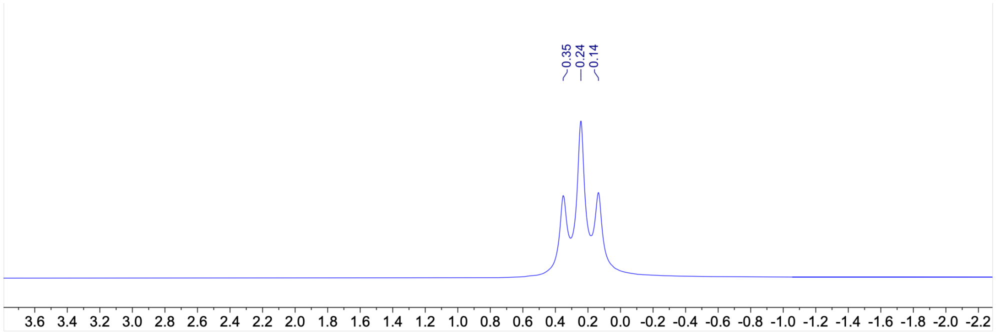

Figure S22: ^{11}B NMR spectrum (in $CDCl_3$, 128 MHz) of $\mathbf{PN}^{tBu}$

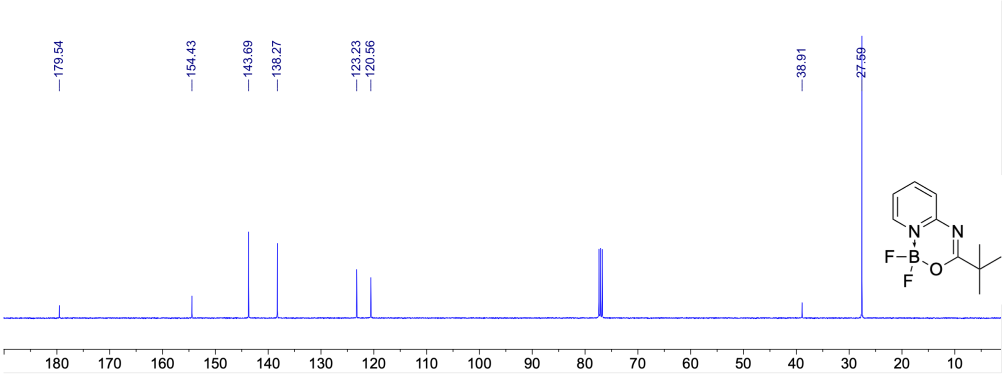

Figure S23: $^{13}C\{^{1}H\}$ NMR spectrum (in $CDCl_3$, 100 MHz) of $\mathbf{PN}^{tBu}$

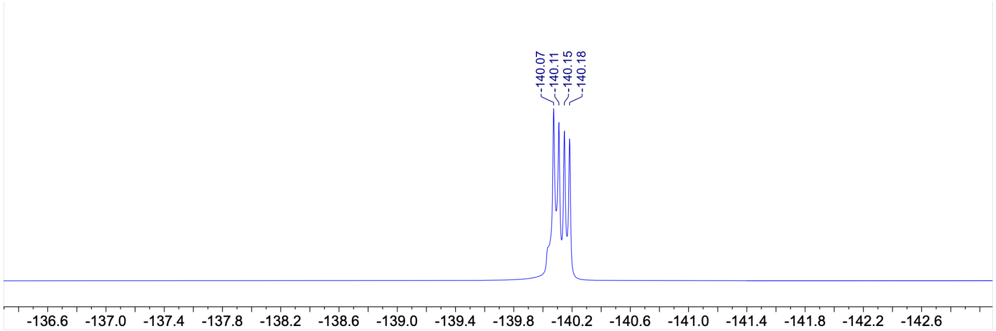

Figure S24: ^{19}F NMR spectrum (in $CDCl_3$, 376 MHz) of $\mathbf{PN}^{tBu}$

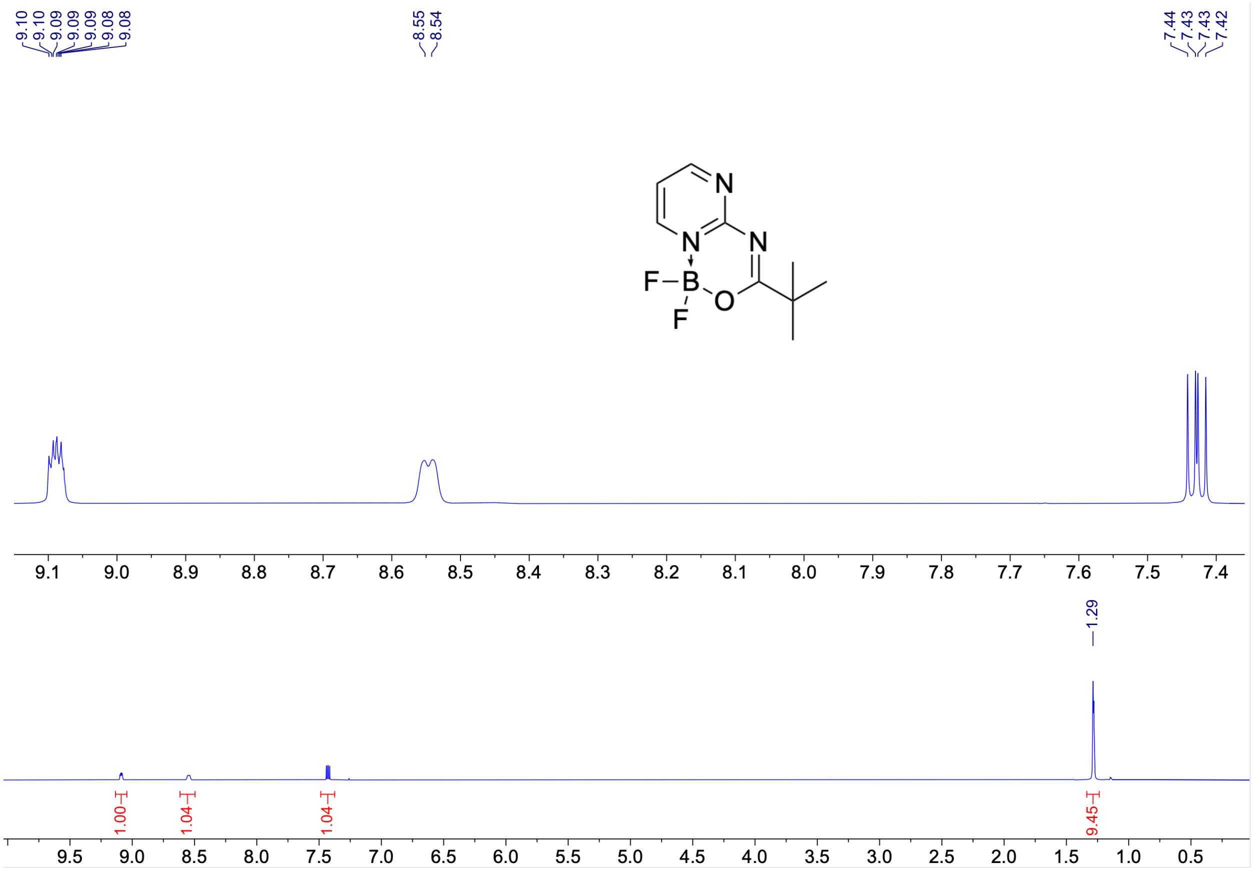

Figure S25: ^{1}H NMR spectrum (in $CDCl_3$, 400 MHz) of **13PN**^{tBu}

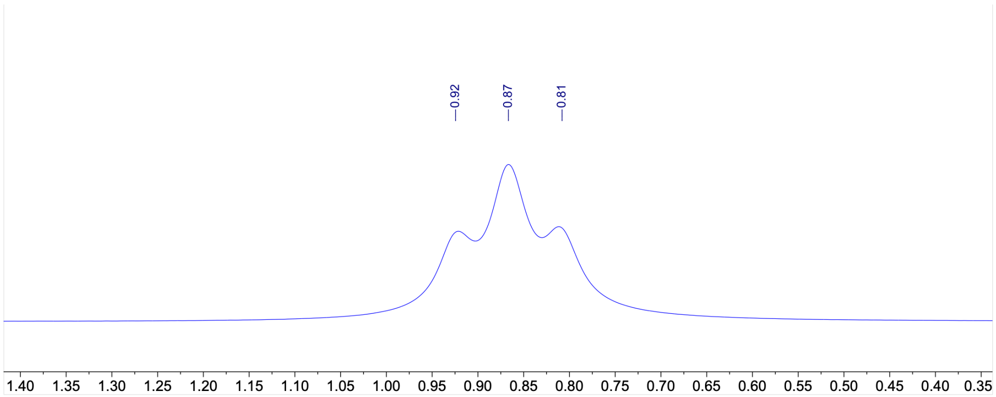

Figure S26: ^{11}B NMR spectrum (in $CDCl_3$, 224 MHz) of **13PN**^{tBu}

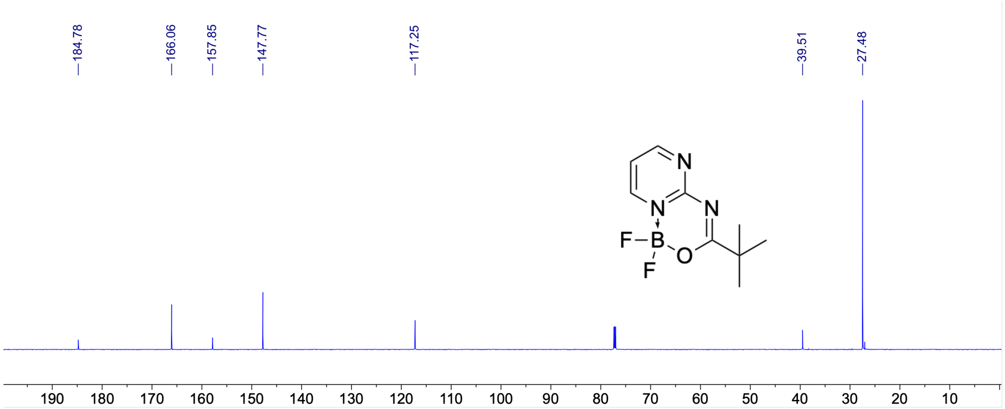

Figure S27: $^{13}C\{^{1}H\}$ NMR spectrum (in $CDCl_3$, 176 MHz) of $\mathbf{13PN}^{tBu}$

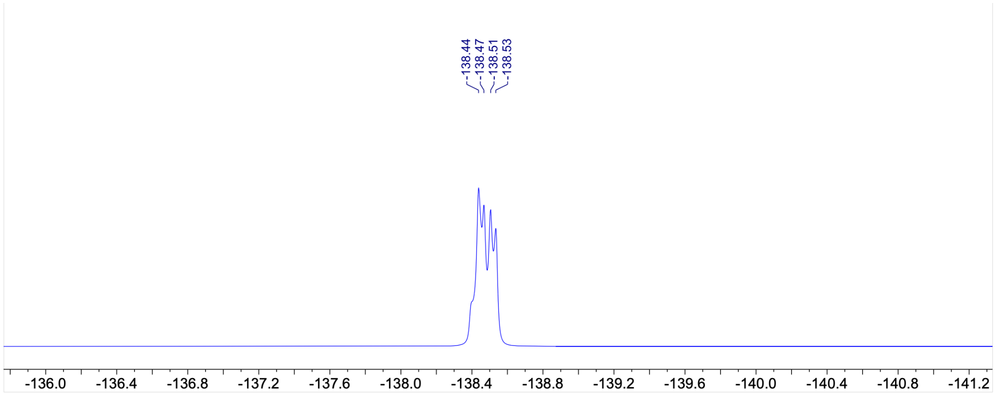

Figure S28: ^{19}F NMR spectrum (in $CDCl_3$, 376 MHz) of $\mathbf{13PN}^{tBu}$

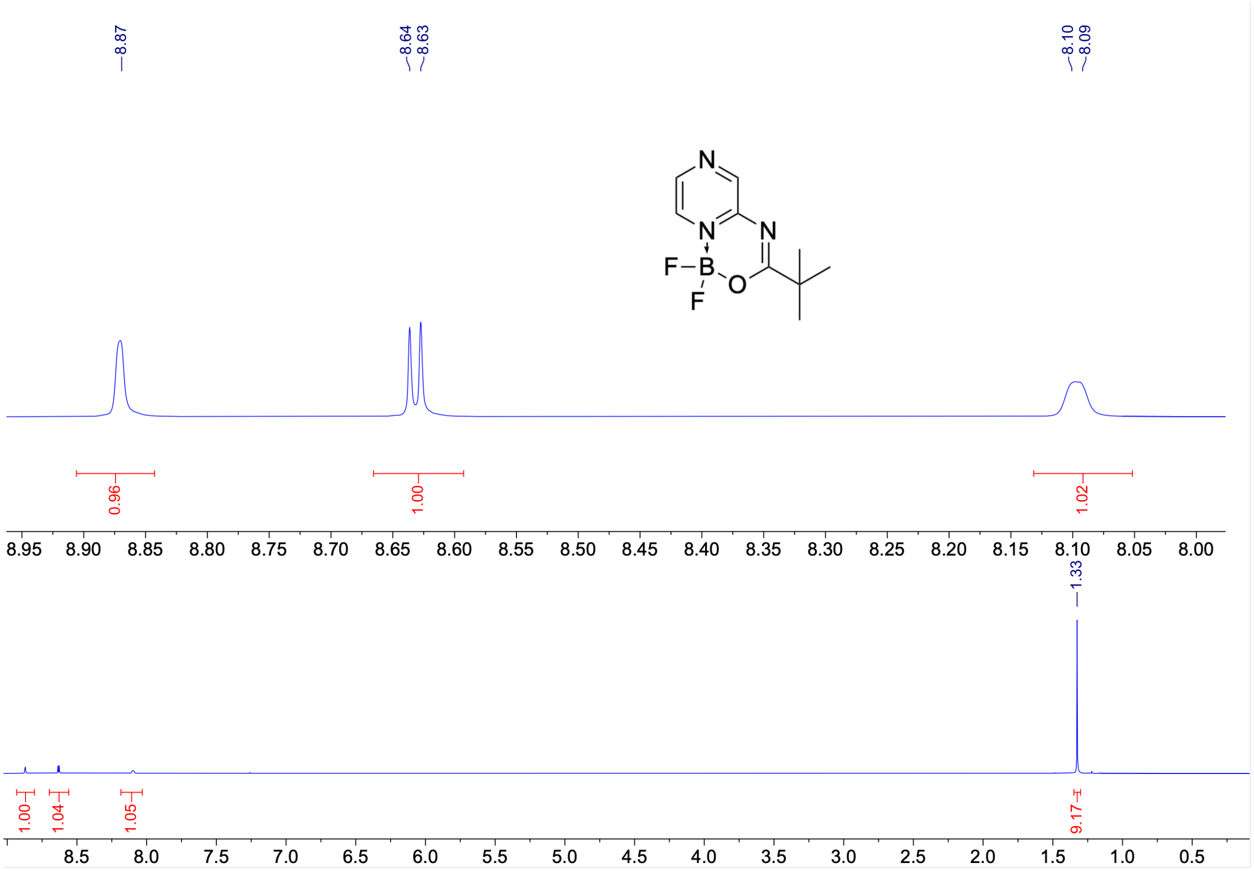

Figure S29: ^{1}H NMR spectrum (in $CDCl_3$, 400 MHz) of **14PN**^{tBu}

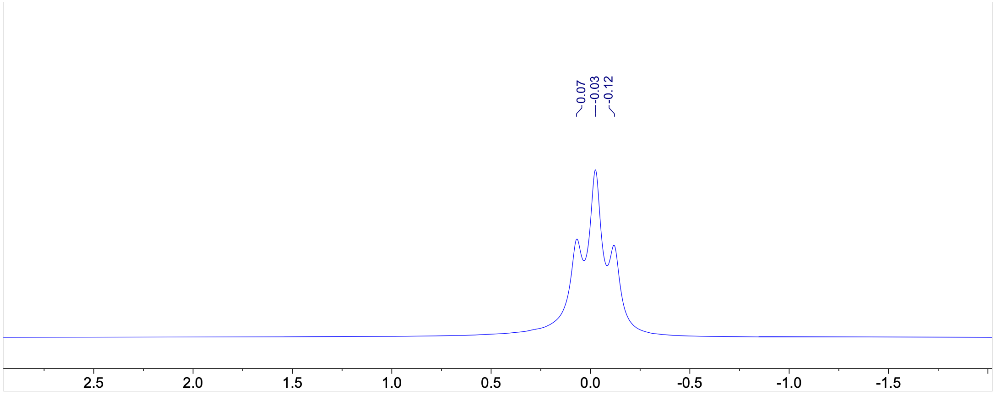

Figure S30: ^{11}B NMR spectrum (in $CDCl_3$, 128 MHz) of **14PN**^{tBu}

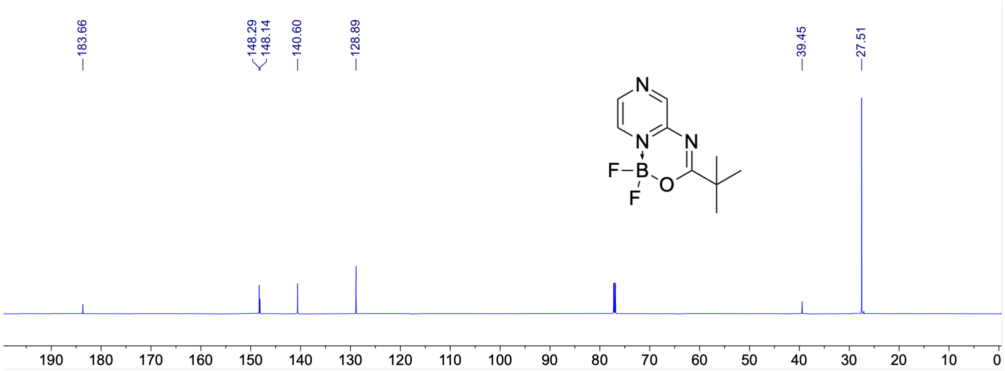

Figure S31: $^{13}C\{^{1}H\}$ NMR spectrum (in $CDCl_3$, 176 MHz) of **14PN**^{tBu}

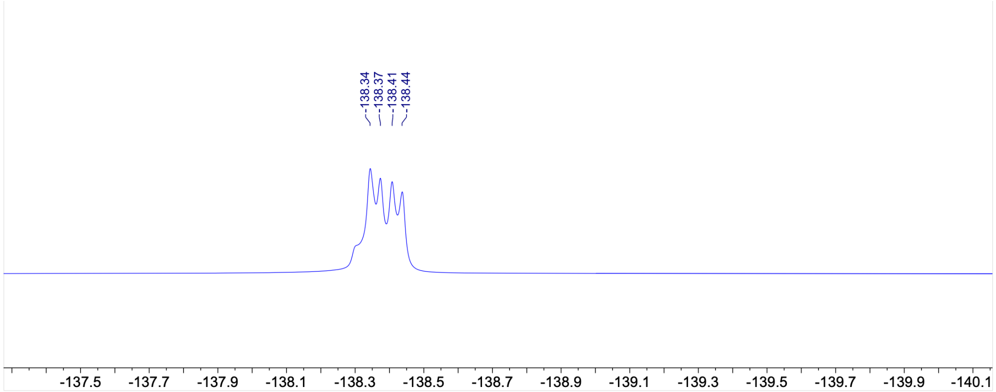

Figure S32: ^{19}F NMR spectrum (in $CDCl_3$, 376 MHz) of **14PN**^{tBu}

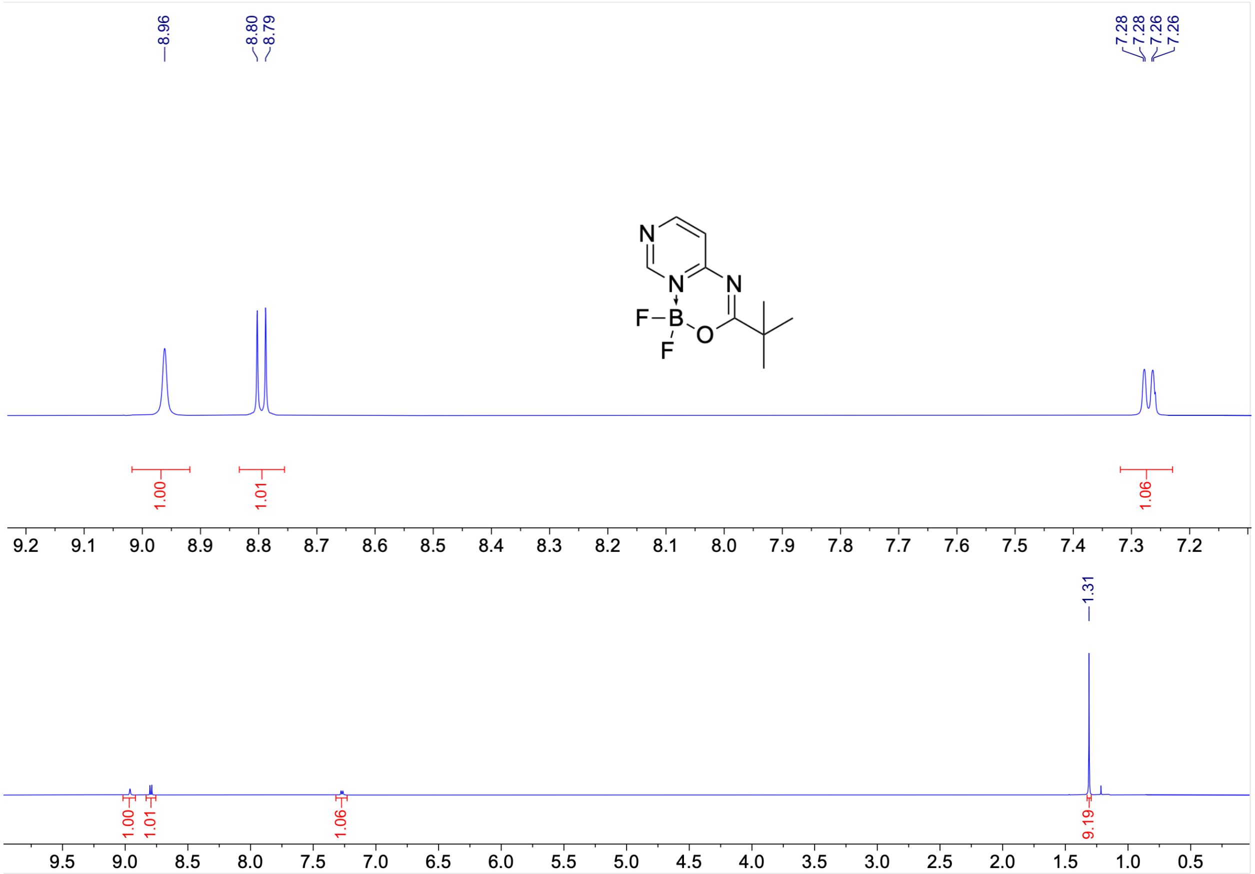

Figure S33: ^{1}H NMR spectrum (in $CDCl_3$, 400 MHz) of $\mathbf{15PN}^{tBu}$

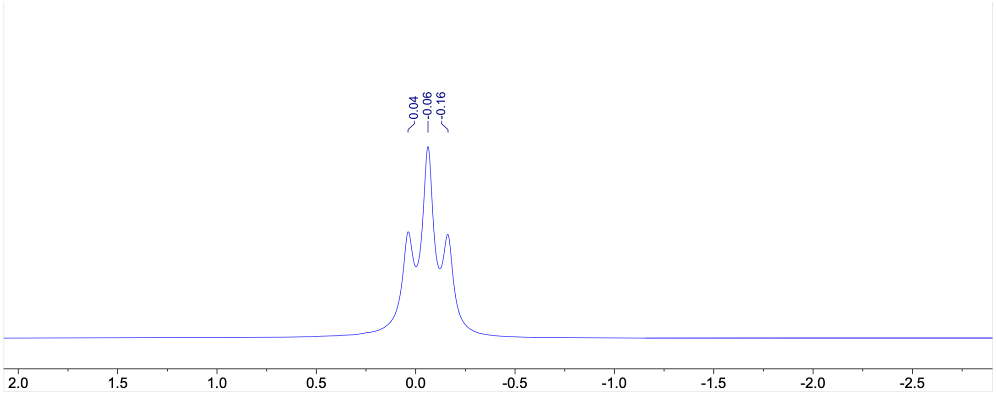

Figure S34: ^{11}B NMR spectrum (in $CDCl_3$, 128 MHz) of $\mathbf{15PN}^{tBu}$

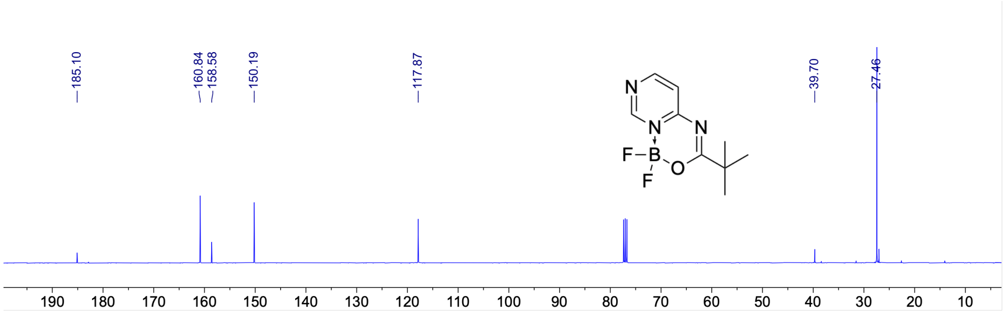

Figure S35: $^{13}C\{^{1}H\}$ NMR spectrum (in $CDCl_3$, 100 MHz) of **15PN**^{tBu}

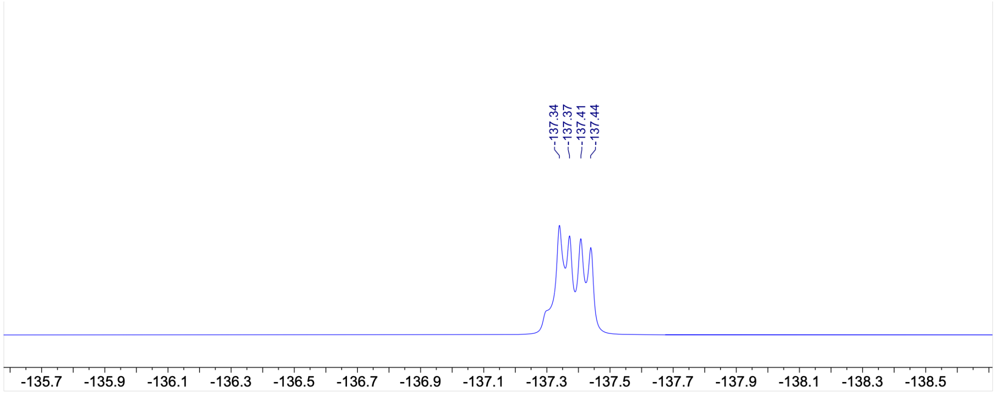

Figure S36: ^{19}F NMR spectrum (in $CDCl_3$, 376 MHz) of **15PN**^{tBu}

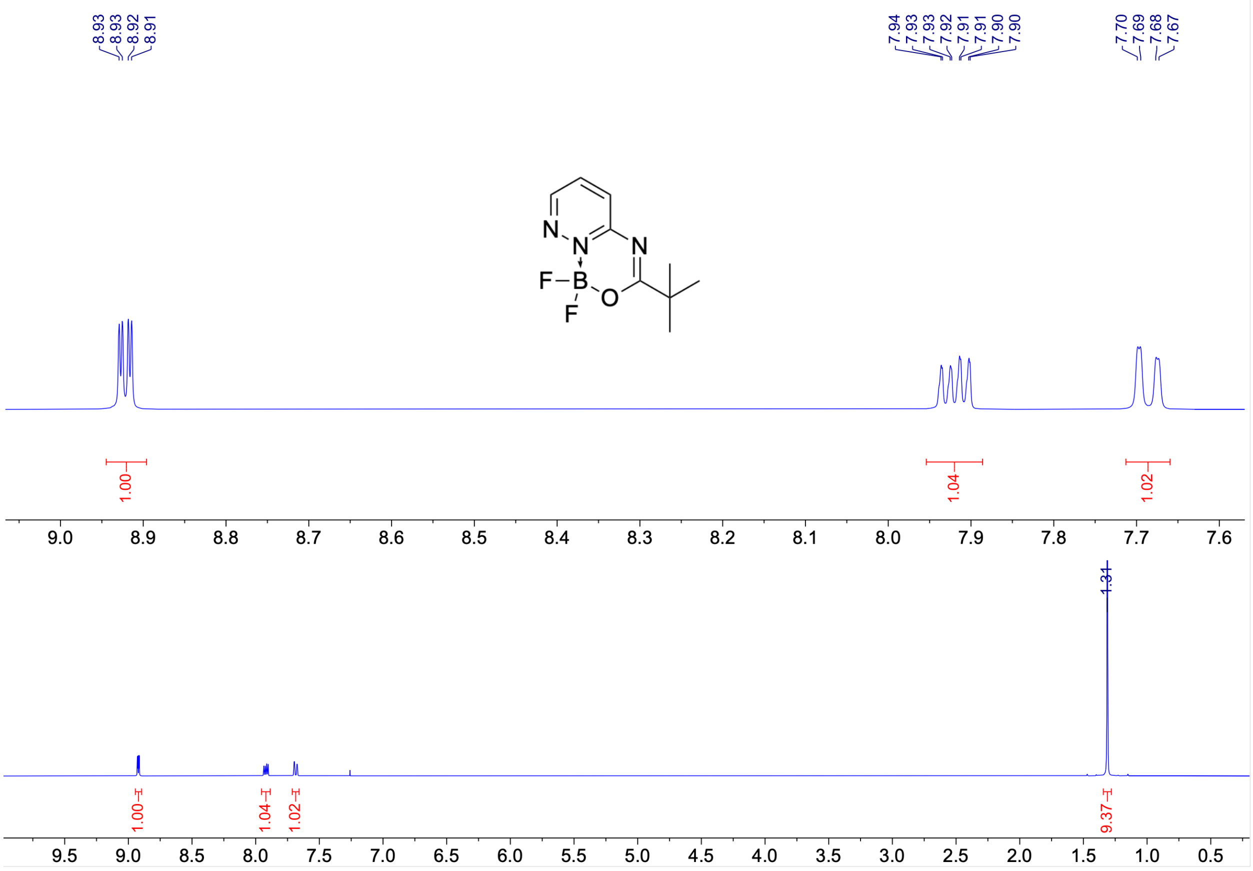

Figure S37: ^{1}H NMR spectrum (in $CDCl_3$, 400 MHz) of **16PN**^{tBu}

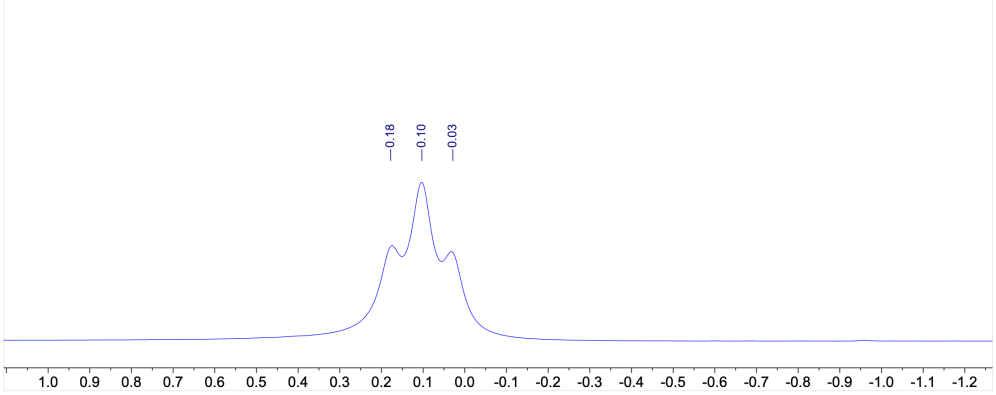

Figure S38: ^{11}B NMR spectrum (in $CDCl_3$, 128 MHz) of **16PN**^{tBu}

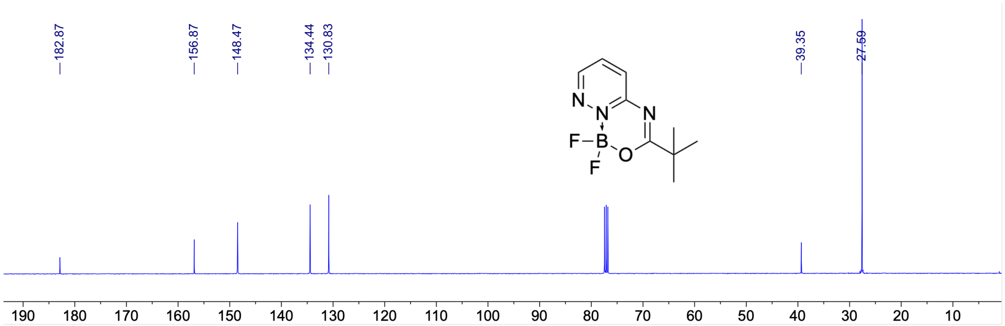

Figure S39: $^{13}C\{^{1}H\}$ NMR spectrum (in $CDCl_3$, 100 MHz) of $\mathbf{16PN}^{tBu}$

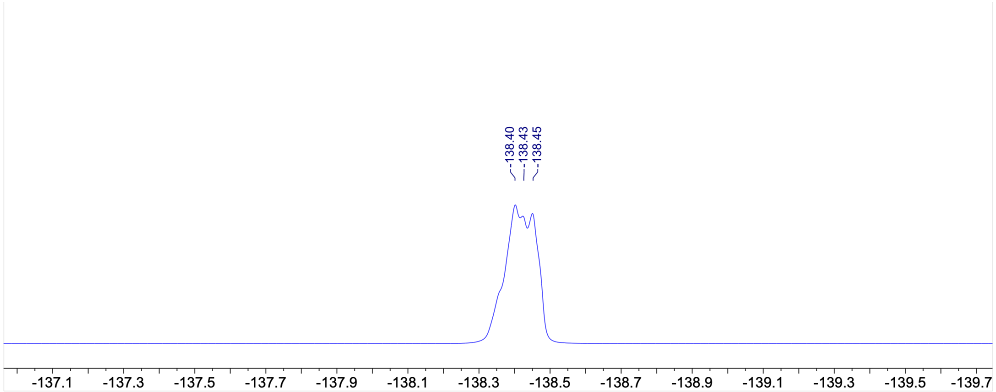

Figure S40: ^{19}F NMR spectrum (in $CDCl_3$, 376 MHz) of $\mathbf{16PN}^{tBu}$

3 Titration charts and CIS values

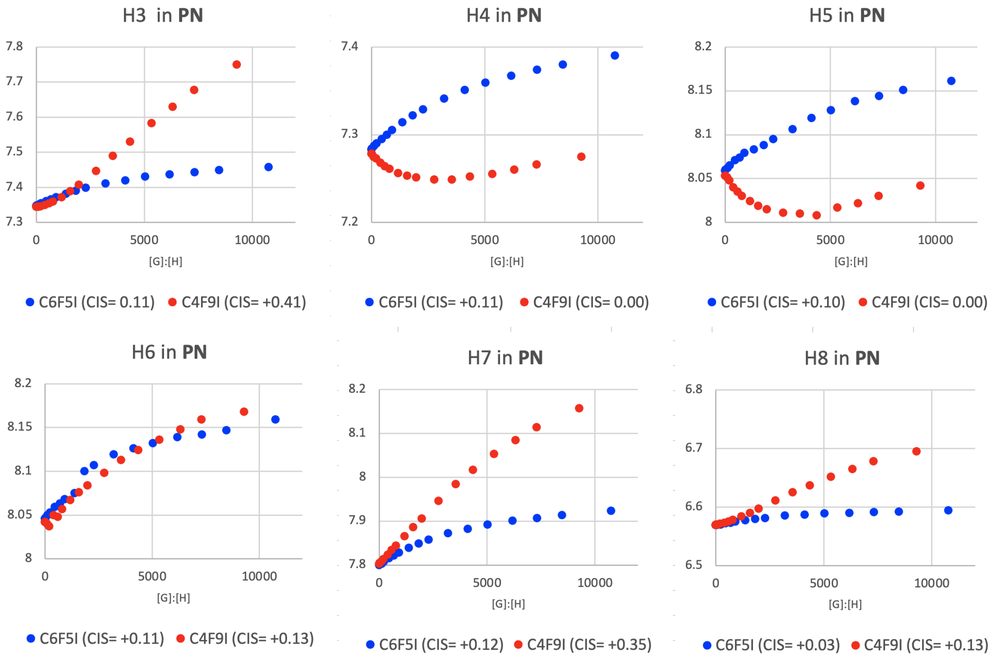

Figure S41: Changes in ^{1}H NMR chemical shifts in **PN** during titration

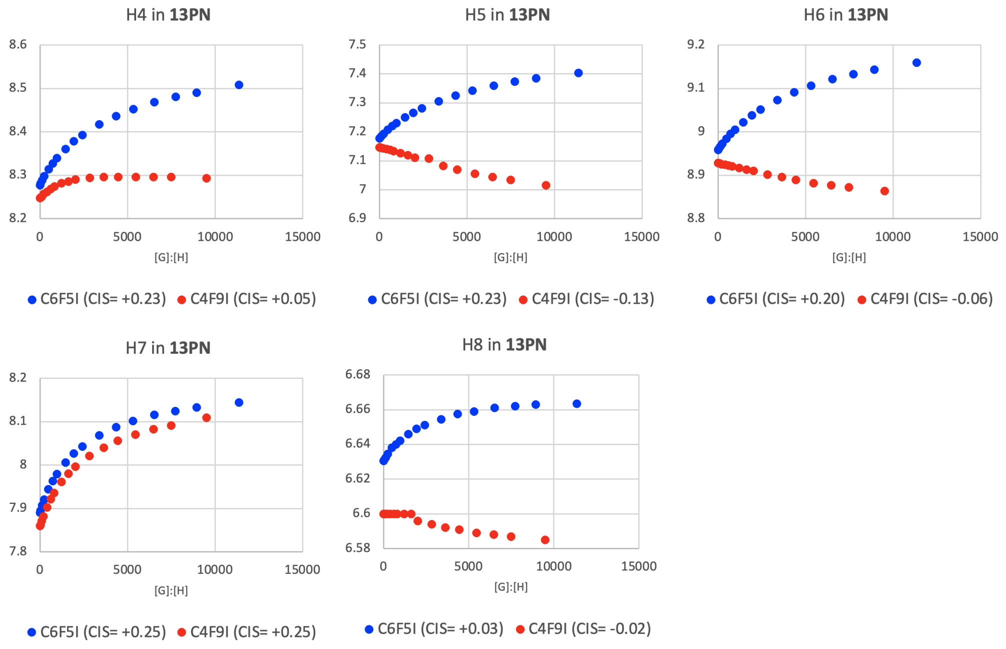

Figure S42: Changes in ^{1}H NMR chemical shifts in **13PN** during titration

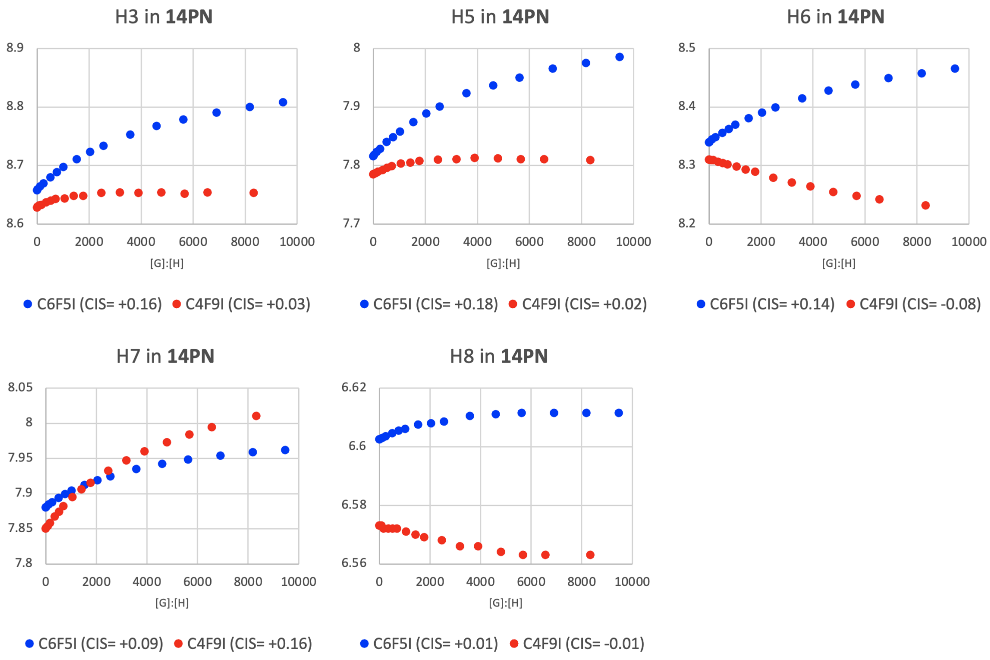

Figure S43: Changes in ^{1}H NMR chemical shifts in **14PN** during titration

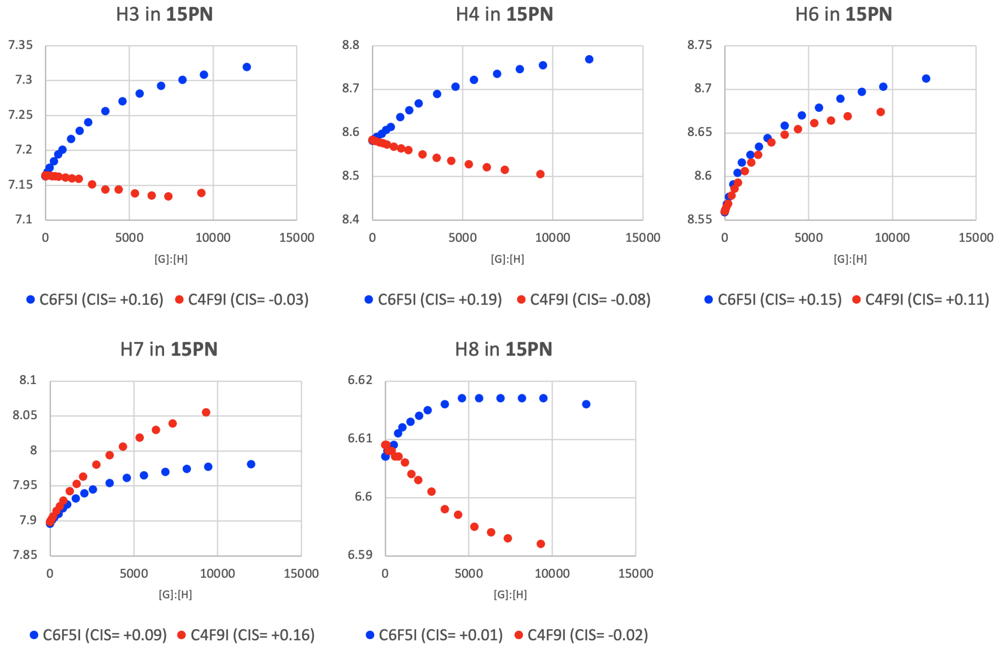

Figure S44: Changes in ^{1}H NMR chemical shifts in **15PN** during titration

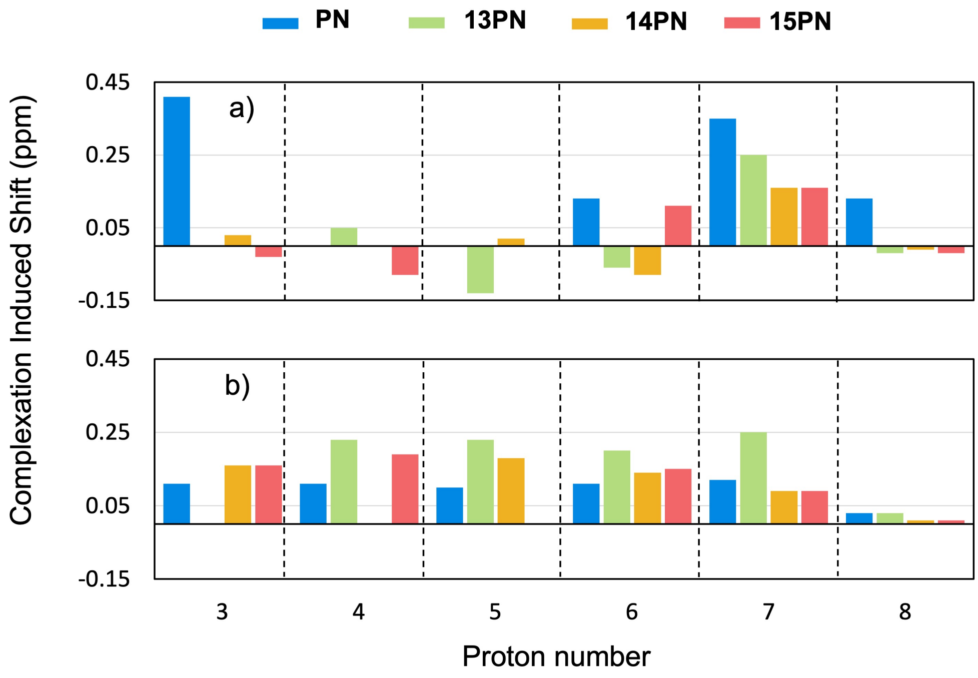

Figure S45: CIS values for every proton of the dyes studied obtained by the NMR titration by the addition of (a) C_4F_9I in C_6F_6 solution and (b) C_6F_5I in C_6F_6 solution.

4 Normalized absorption and fluorescence spectra

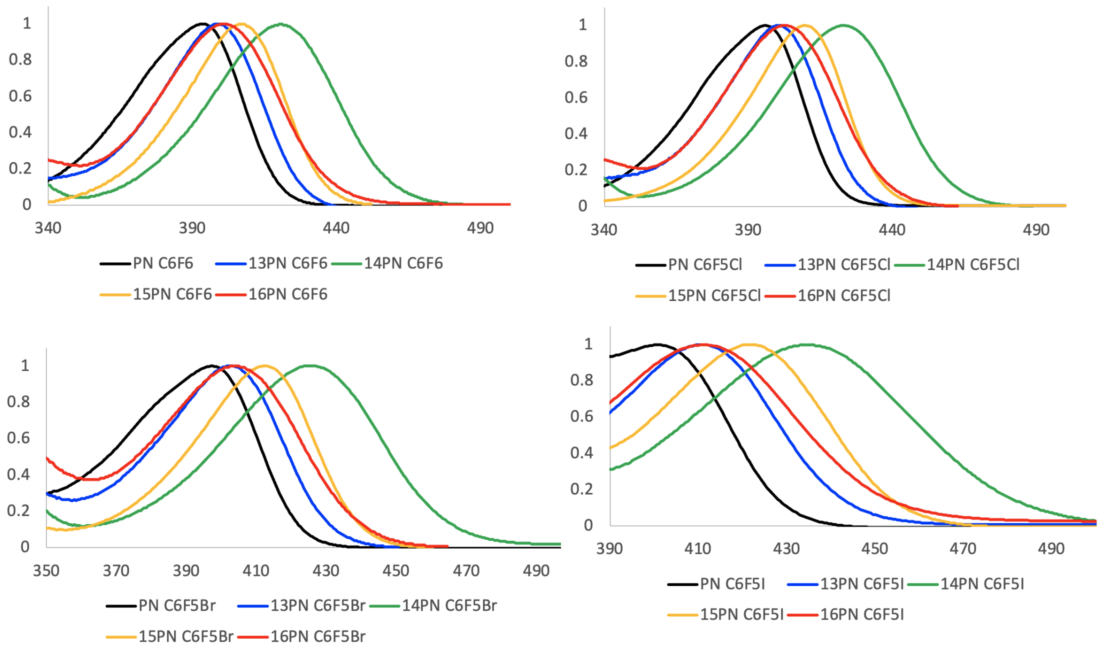

Figure S46: The normalized absorption spectra for **PN-16PN** in chosen solvents

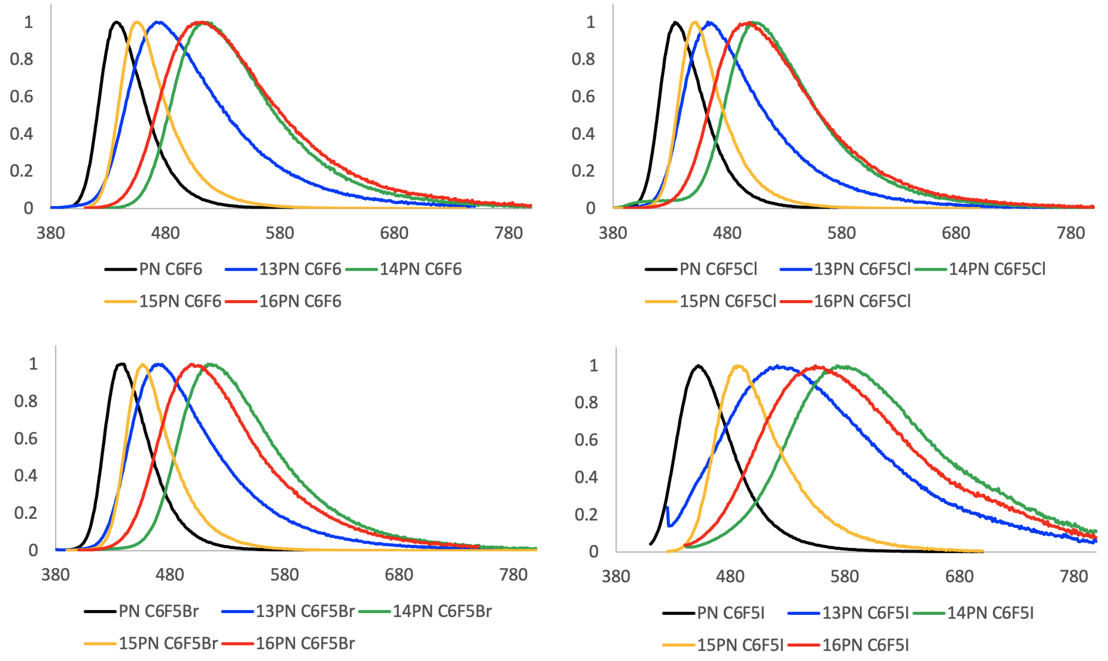

Figure S47: The normalized fluorescence spectra for **PN-16PN** in chosen solvents

5 Geometries

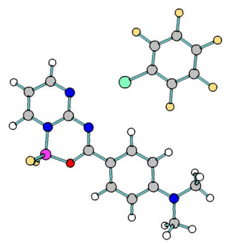

13PN:C_6F_5Cl

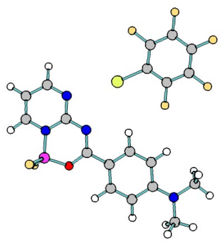

13PN C_6F_5Br

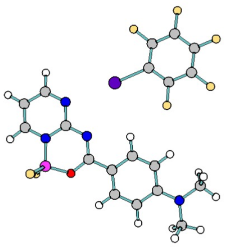

13PN:C_6F_5I

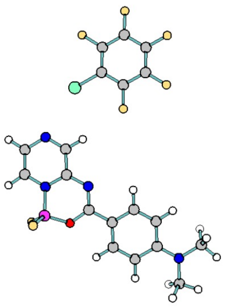

14PN:C_6F_5Cl

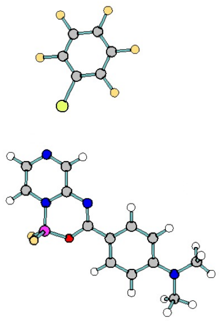

14PN:C_6F_5Br

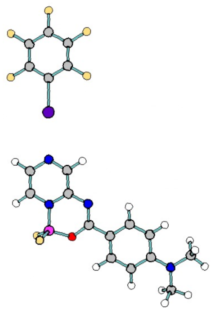

14N:C_6F_5I

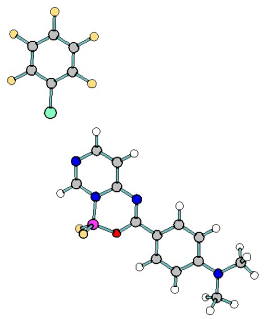

15PN:C_6F_5Cl

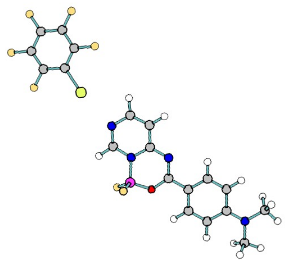

15PN:C_6F_5Br

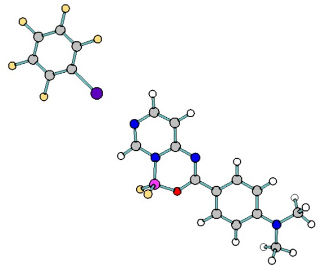

15PN:C_6F_5I

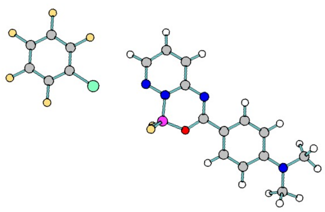

16PN:C_6F_5Cl

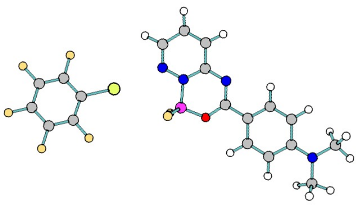

16PN:C_6F_5Br

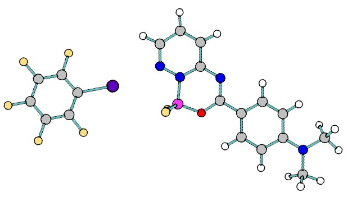

16PN:C_6F_5I

Figure S48: Equilibrium geometries of complexes formed by **xPN** and the halogen bond donors studied interacting through halogen bonding in the *heterocyclic* nitrogen, obtained at the MN15/aug-cc-pVDZ(PP) level of theory.

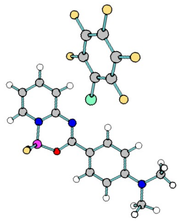

PN:C_6F_5Cl

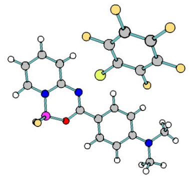

PN:C_6F_5Br

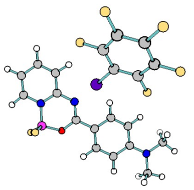

PN:C_6F_5I

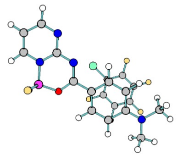

13PN:C_6F_5Cl

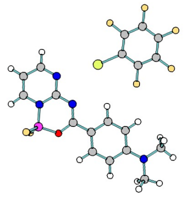

13PN:C_6F_5Br

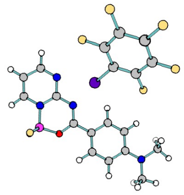

13PN:C_6F_5I

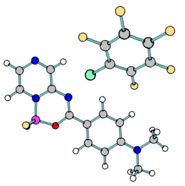

14PN:C_6F_5Cl

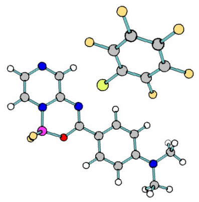

14PN:C_6F_5Br

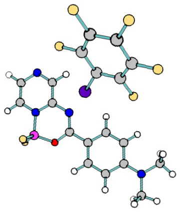

14PN:C_6F_5I

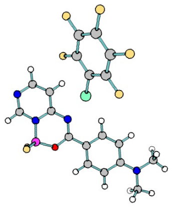

15PN:C_6F_5Cl

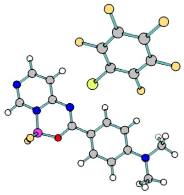

15PN:C_6F_5Br

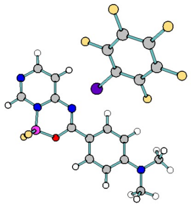

15PN:C_6F_5I

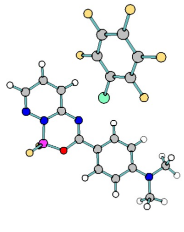

16PN:C_6F_5Cl

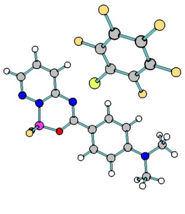

16PN:C_6F_5Br

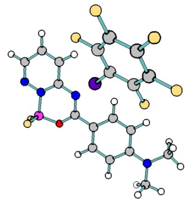

16PN:C_6F_5I

Figure S49: Equilibrium geometries of complexes formed by **xPN** and the halogen bond donors studied interacting through halogen bonding in the *imine* nitrogen, obtained at the MN15/aug-cc-pVDZ(PP) level of theory.

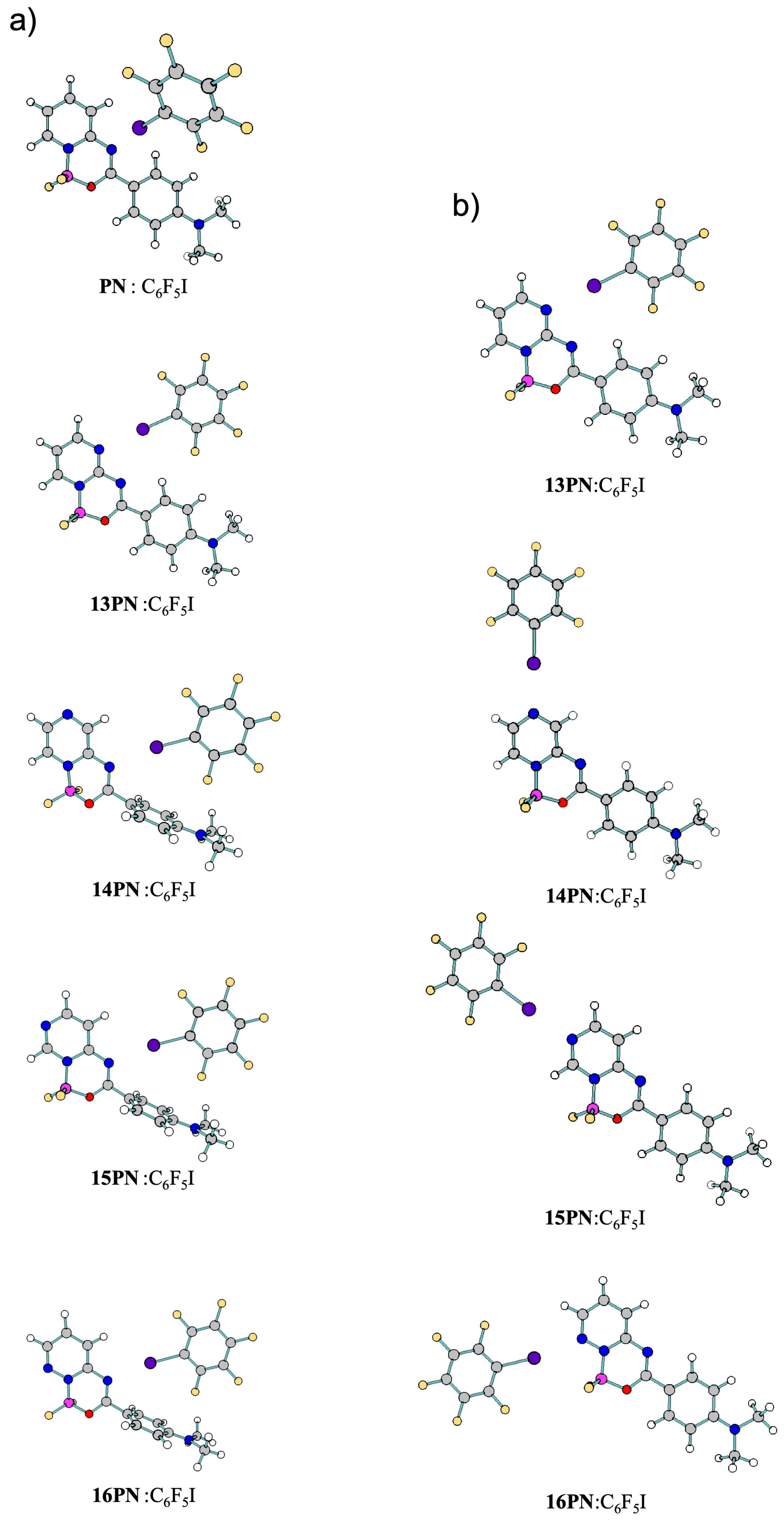

Figure S50: Equilibrium excited state geometries of complexes formed by **xPN** and C_6F_5I interacting through halogen bonding at the a) *imine* and b) *heterocyclic* nitrogen atom.

6 Calculated photophysical data

Table S1: Summary of the electronic structure calculations of **xPN** dyes and their complexes formed with C_6F_5X halogen bond donors interacting at the heterocyclic nitrogen using TD-DFT at MN15/aug-cc-pVDZ level of theory. Maximum absorption wavelength (λ_{abs}), maximum emission wavelength (λ_{em}), oscillator strengths (f), and Stokes shift.

Compound	Halogen bond donor	λ_{abs} nm	f	λ_{em} nm	f	Stokes cm^{-1}
**PN**	none	348	0.906	368	0.815	1579
**13PN**	none	349	0.830	452	0.078	6523
	C_6F_5Cl	354	0.752	468	0.063	6909
	C_6F_5Br	357	0.720	489	0.048	7535
	C_6F_5I	361	0.682	513	0.036	8159
**14PN**	none	368	0.766	438	0.305	4376
	C_6F_5Cl	370	0.738	447	0.288	4633
	C_6F_5Br	377	0.746	468	0.260	5202
	C_6F_5I	382	0.761	492	0.242	5834
**15PN**	none	351	0.949	368	0.839	1367
	C_6F_5Cl	352	1.047	371	0.930	1448
	C_6F_5Br	357	1.109	379	0.917	1668
	C_6F_5I	361	1.165	388	0.901	1988
**16PN**	none	356	0.639	453	0.126	6029
	C_6F_5Cl	357	0.714	463	0.127	6395
	C_6F_5Br	364	0.665	478	0.124	6581
	C_6F_5I	367	0.668	493	0.124	6927

Table S2: Summary of electronic structure calculations of complexes formed by **xPN** interacting with C_6F_5I at imine nitrogen, calculated at TD-DFT level. Maximum absorption wavelength (λ_{abs}), Maximum emission wavelength (λ_{em}), oscillator strengths (f), and Stokes shift.

Compound	λ_{abs} nm	f	λ_{em} nm	f	Stokes cm^{-1}
**PN**	351	0.800	372	0.727	1608
**13PN**	355	0.731	513	0.036	8676
**14PN**	371	0.685	569	0.000	9379
**15PN**	356	0.822	509	0.001	8444
**16PN**	360	0.588	562	0.000	9984

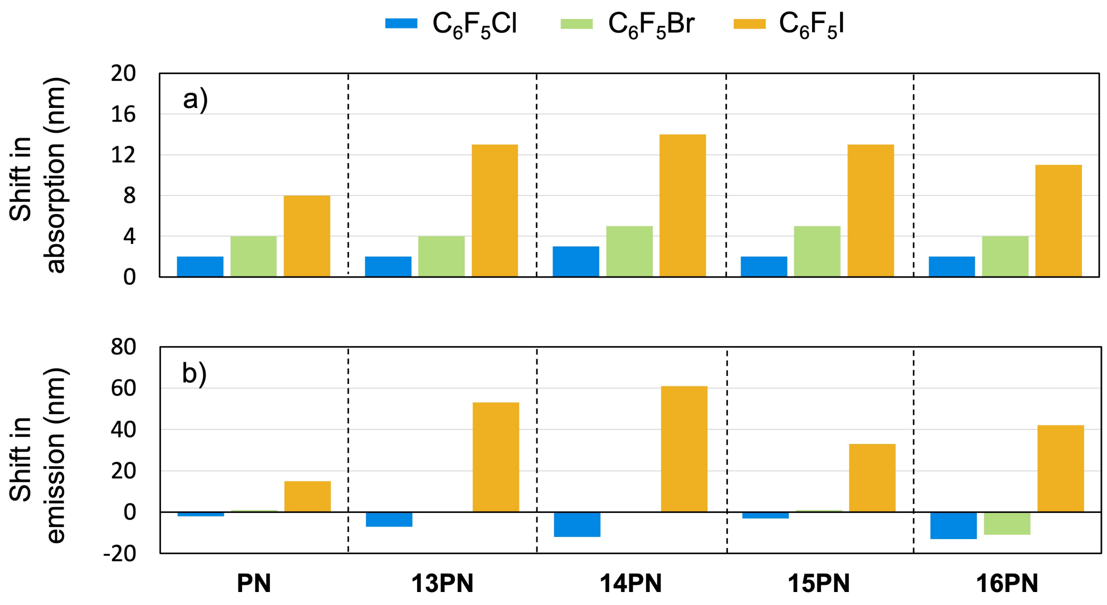

Figure S51: Changes in (a) absorption maximum wavelength for dyes in C_6F_5X with respect to C_6F_6 and (b) emission maximum wavelength for dyes in C_6F_5X with respect to C_6F_6. *Note the different scales for both panels.*

We have tested the effect of the solvent in the shift of the emission maximum wavelength using the PCM solvation model. Based on those calculations it may be safely concluded that the polarity of the solvent has minimal influence on the emission spectra, as indicated by the blue line in the graph (Fig. S52).

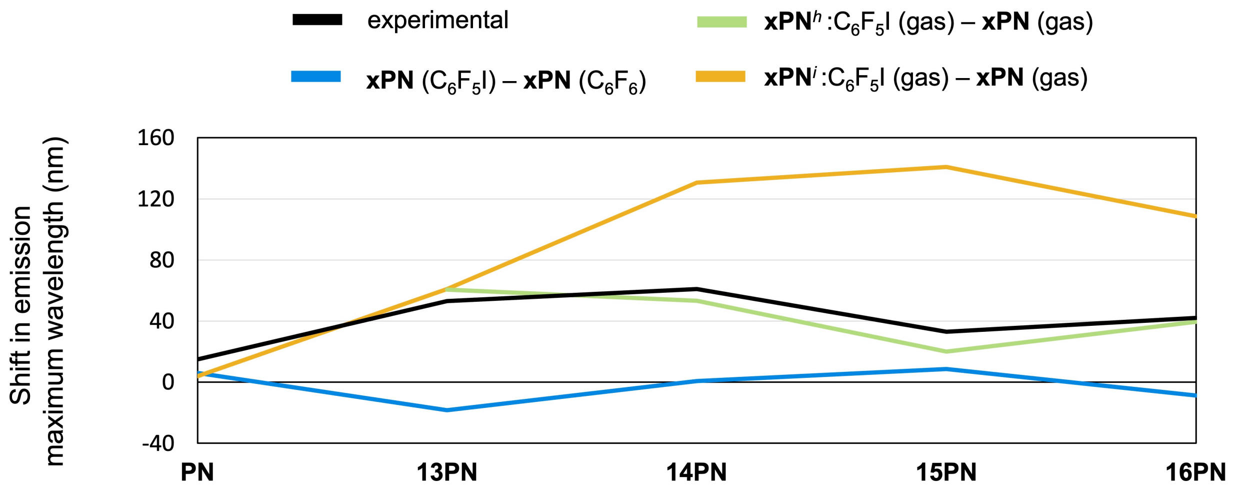

Figure S52: Effect of the solvent in the shift in the emission maximum wavelength of the isolated dye using PCM solvation model (blue), shift in emission maximum wavelength upon complex formation at heterocyclic nitrogen in the gas phase (green), shift in emission maximum wavelength upon complex formation at imine nitrogen in the gas phase (orange), and comparison with experimental values (black).

More significantly, the complexes where the heterocyclic nitrogen acts as the halogen bond acceptor (green line) closely mirror the experimental emission data, providing strong evidence that halogen bonding at this site plays a crucial role in determining the photophysical properties of the dyes. The simulated emission maximum wavelength was computed using the excited-state equilibrium geometries for complexes with C_6F_5X interacting at the heterocyclic nitrogen (Fig. SS1). The results indicate that as the halogen atom in the complexes changes (Cl, Br, I), a consistent red-shift is observed, with the shift magnitude correlating with the strength of the interaction in the order Cl, Br, and I.

7 Crucial interatomic distances and angled, and interaction energies

Table S3: Halogen bond distance (N$\cdots$I) in Å at the equilibrium geometry. In parenthesis C–I$\cdots$N angles in degrees at the equilibrium geometry.

dye	C_6F_5Cl	C_6F_5Br	C_6F_5I
		heterocyclic nitrogen atom	
**13PN**	3.26 (166.13)	3.06 (171.58)	3.01 (177.37)
**14PN**	3.20 (148.87)	2.92 (172.34)	2.90 (179.72)
**15PN**	3.18 (149.61)	2.91 (170.76)	2.91 (174.97)
**16PN**	3.19 (144.92)	2.95 (169.19)	3.01 (167.67)
		imine nitrogen atom	
**PN**	3.12 (158.73)	2.92 (177.36)	2.97 (176.73)
**13PN**	π-stacking	3.06 (171.54)	3.02 (173.93)
**14PN**	3.12 (167.04)	2.94 (176.55)	3.00 (176.32)
**15PN**	3.13 (159.03)	2.94 (176.60)	3.00 (176.67)
**16PN**	3.15 (156.57)	2.94 (176.59)	2.99 (175.85)

Table S4: Intermolecular interaction energy (ΔE, in kcal/mol) for halogen-bonded complexes between **xPN**-series dyes and C_6F_5X at the heterocyclic and imine nitrogen atoms, calculated using the DF-SCS-MP2 method and the aug-cc-pVDZ(PP) basis set.

dye	C_6F_5Cl	C_6F_5Br	C_6F_5I
	heterocyclic nitrogen atom		
**13PN**	-3.43	-4.83	-6.40
**14PN**	-1.97	-3.05	-4.64
**15PN**	-1.98	-3.00	-4.43
**16PN**	-2.53	-3.41	-4.72
	imine nitrogen atom		
**PN**	-3.03	-4.24	-5.73
**13PN**	-	-	-5.72
**14PN**	-2.79	-3.86	-5.09
**15PN**	-2.88	-3.89	-5.17
**16PN**	-3.11	-4.10	-5.40

8 Additional figures

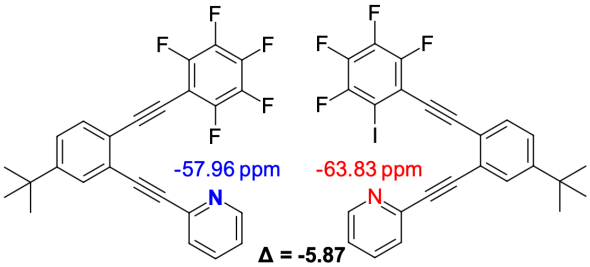

Figure S53: The effect of intramolecular halogen bond on ^{15}N NMR chemical shift

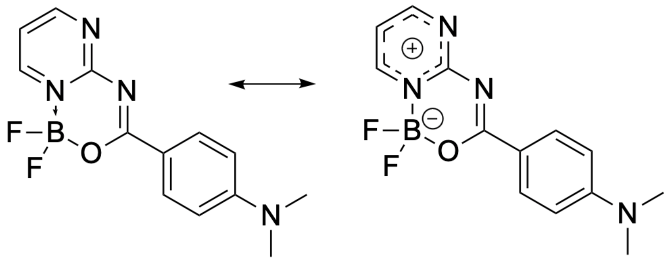

Figure S54: Electron withdrawing effect close to the BF_2 moiety (exemplified on **13PN**) causing electron density decrease in the heterocyclic ring

9 Cartesian coordinates

For all optimized structures the frequency calculations confirmed the geometry is in energy minima (no imaginary frequencies).

Table S5: Cartesian coordinates and total energy in a.u. of the ground state optimized geometry of **PN** dye at the MN15/aug-cc-pVDZ level of theory.

Atomic Number	X (Å)	Y (Å)	Z (Å)
6	1.777945000	-1.153282000	-0.124097000
6	1.013337000	0.020245000	-0.024477000
6	3.163754000	-1.106758000	-0.124259000

1	3.720281000	-2.037935000	-0.201837000
6	1.684311000	1.250117000	0.076769000
6	3.846884000	0.131023000	-0.022041000
1	1.093504000	2.161749000	0.153796000
6	3.068271000	1.312598000	0.079280000
1	3.550879000	2.284002000	0.159868000
1	1.267256000	-2.111986000	-0.199694000
7	5.218051000	0.185057000	-0.020682000
6	5.892790000	1.461806000	0.087738000
1	5.636210000	2.128061000	-0.750416000
1	5.637363000	1.976261000	1.027008000
1	6.973469000	1.294614000	0.072787000
6	5.990801000	-1.035368000	-0.126601000
1	7.055090000	-0.784192000	-0.110414000
1	5.785505000	-1.718628000	0.711879000
1	5.777322000	-1.569017000	-1.065552000
6	-0.448577000	-0.037012000	-0.023960000
8	-0.968286000	-1.232603000	-0.180499000
7	-1.143295000	1.074007000	0.099190000
5	-2.379485000	-1.523320000	0.095776000
6	-2.500140000	1.017835000	0.022419000
6	-3.248324000	2.218139000	0.000806000
7	-3.169472000	-0.161514000	-0.047274000
6	-4.623112000	2.171954000	-0.099062000
6	-5.283905000	0.929043000	-0.180902000
6	-4.517654000	-0.213483000	-0.148351000
9	-2.548409000	-1.972442000	1.399784000

9	-2.883974000	-2.419017000	-0.832357000
1	-4.935883000	-1.217534000	-0.199840000
1	-6.366094000	0.859917000	-0.261724000
1	-5.198620000	3.097864000	-0.116975000
1	-2.696393000	3.153381000	0.061177000
**Total Energy**		-1005.17155684	

Table S6: Cartesian coordinates and total energy in a.u. of the ground state optimized geometry of **13PN** dye at the MN15/aug-cc-pVDZ level of theory.

Atomic Number	X (Å)	Y (Å)	Z (Å)
6	-1.768678000	1.164629000	-0.106318000
6	-1.002340000	-0.010017000	-0.021677000
6	-3.153351000	1.115404000	-0.106097000
1	-3.712360000	2.045906000	-0.171818000
6	-1.669897000	-1.244073000	0.064567000
6	-3.833672000	-0.126193000	-0.018791000
1	-1.075186000	-2.154140000	0.129385000
6	-3.052982000	-1.308268000	0.067070000
1	-3.534085000	-2.281195000	0.135432000
1	-1.259925000	2.125176000	-0.170696000
7	-5.203384000	-0.182578000	-0.017049000
6	-5.876445000	-1.462244000	0.075379000
1	-5.619334000	-2.116546000	-0.771697000
1	-5.618938000	-1.987816000	1.007636000
1	-6.957257000	-1.296154000	0.063679000
6	-5.979401000	1.037562000	-0.107868000
1	-7.042927000	0.783412000	-0.093883000

1	-5.774924000	1.710738000	0.738737000
1	-5.767715000	1.582454000	-1.040589000
6	0.456233000	0.046198000	-0.020884000
8	0.978707000	1.242278000	-0.154196000
7	1.143354000	-1.075940000	0.085781000
5	2.396664000	1.521880000	0.083510000
6	2.490446000	-1.040503000	0.021220000
7	3.155568000	-2.218147000	0.013472000
7	3.164690000	0.143906000	-0.043002000
6	4.473960000	-2.187083000	-0.074023000
6	5.227860000	-1.001001000	-0.164724000
6	4.508484000	0.170618000	-0.139457000
9	2.605974000	2.001442000	1.369212000
9	2.901101000	2.380745000	-0.878979000
1	4.953178000	1.163992000	-0.191835000
1	6.311739000	-1.007230000	-0.241162000
1	4.978182000	-3.156766000	-0.074065000
**Total Energy**		-1021.20271630	

Table S7: Cartesian coordinates and total energy in a.u. of the ground state optimized geometry of **14PN** dye at the MN15/aug-cc-pVDZ level of theory.

Atomic Number	X (Å)	Y (Å)	Z (Å)
6	-1.769552000	1.157218000	-0.127856000
6	-1.005358000	-0.017379000	-0.023430000
6	-3.154300000	1.111137000	-0.128732000
1	-3.710734000	2.041955000	-0.209829000
6	-1.676910000	-1.247715000	0.081865000

6	-3.838188000	-0.127000000	-0.022364000
1	-1.087240000	-2.159714000	0.162406000
6	-3.060005000	-1.309324000	0.083720000
1	-3.543282000	-2.280000000	0.167343000
1	-1.258619000	2.115543000	-0.206450000
7	-5.207522000	-0.180272000	-0.021521000
6	-5.883872000	-1.456657000	0.091066000
1	-5.627497000	-2.125309000	-0.744945000
1	-5.628966000	-1.967383000	1.032238000
1	-6.964222000	-1.288299000	0.075280000
6	-5.980711000	1.040189000	-0.133045000
1	-7.044800000	0.788768000	-0.116779000
1	-5.776056000	1.726153000	0.703112000
1	-5.766282000	1.569429000	-1.074035000
6	0.452721000	0.038746000	-0.021277000
8	0.979878000	1.229781000	-0.182245000
7	1.149972000	-1.075641000	0.105594000
5	2.389602000	1.529243000	0.098823000
6	2.498754000	-1.006473000	0.026105000
6	3.271982000	-2.204014000	-0.003237000
7	3.175804000	0.160574000	-0.043273000
7	4.582268000	-2.209721000	-0.105662000
6	5.219865000	-1.016787000	-0.187321000
6	4.523592000	0.168901000	-0.153974000
9	2.555338000	1.969116000	1.403316000
9	2.899329000	2.417233000	-0.829210000
1	2.739675000	-3.153467000	0.059847000

1	4.992647000	1.149562000	-0.211015000
1	6.304582000	-1.028471000	-0.275355000
**Total Energy**		-1021.19535072	

Table S8: Cartesian coordinates and total energy in a.u. of the ground state optimized geometry of **15PN** dye at the MN15/aug-cc-pVDZ level of theory.

Atomic Number	X (Å)	Y (Å)	Z (Å)
6	1.770726000	-1.155798000	-0.117586000
6	1.007499000	0.020808000	-0.022997000
6	3.155084000	-1.111270000	-0.117711000
1	3.710643000	-2.043237000	-0.191187000
6	1.680345000	1.251422000	0.073045000
6	3.840343000	0.127319000	-0.020446000
1	1.091294000	2.164411000	0.145982000
6	3.063414000	1.311550000	0.075546000
1	3.547845000	2.282251000	0.151913000
1	1.258697000	-2.114081000	-0.189354000
7	5.209189000	0.178762000	-0.018762000
6	5.887345000	1.455186000	0.083838000
1	5.632546000	2.117113000	-0.757933000
1	5.632311000	1.973837000	1.020577000
1	6.967438000	1.285179000	0.070367000
6	5.981301000	-1.043538000	-0.120099000
1	7.045604000	-0.793114000	-0.104347000
1	5.774796000	-1.722704000	0.721040000
1	5.767452000	-1.579526000	-1.057320000
6	-0.449973000	-0.035161000	-0.021359000

8	-0.975390000	-1.225028000	-0.165813000
7	-1.143358000	1.086831000	0.093206000
5	-2.394736000	-1.518121000	0.092111000
6	-2.490079000	1.030895000	0.021256000
6	-3.265837000	2.213382000	0.000130000
7	-3.169359000	-0.147165000	-0.046655000
6	-4.633428000	2.097450000	-0.097671000
7	-5.280401000	0.906288000	-0.178158000
6	-4.517503000	-0.160421000	-0.145845000
1	-4.968768000	-1.150652000	-0.203025000
9	-2.571803000	-1.976373000	1.388880000
9	-2.888752000	-2.398407000	-0.851983000
1	-2.760096000	3.173952000	0.056374000
1	-5.265885000	2.986465000	-0.117238000
**Total Energy**		-1021.20617116	

Table S9: Cartesian coordinates and total energy in a.u. of the ground state optimized geometry of **16PN** dye at the MN15/aug-cc-pVDZ level of theory.

**Atomic Number**	**X (Å)**	**Y (Å)**	**Z (Å)**
6	1.773527000	-1.157857000	-0.000507000
6	1.010683000	0.021960000	-0.000418000
6	3.158494000	-1.113511000	-0.000563000
1	3.713561000	-2.048728000	-0.000515000
6	1.683160000	1.255461000	-0.000254000
6	3.843903000	0.128264000	-0.000614000
1	1.094997000	2.172000000	-0.000044000
6	3.067075000	1.315841000	-0.000308000

1	3.551177000	2.289813000	-0.000054000
1	1.261153000	-2.118713000	-0.000493000
7	5.213901000	0.179422000	-0.000998000
6	5.891557000	1.459608000	0.000953000
1	5.636133000	2.053628000	-0.890005000
1	5.637085000	2.050813000	0.894118000
1	6.971799000	1.289296000	-0.000026000
6	5.984935000	-1.047242000	0.000774000
1	7.049527000	-0.797033000	-0.000059000
1	5.774711000	-1.655729000	0.893695000
1	5.774127000	-1.658269000	-0.890212000
6	-0.449678000	-0.036381000	-0.000356000
8	-0.971239000	-1.229126000	-0.000472000
7	-1.142017000	1.092141000	-0.000083000
5	-2.423112000	-1.538390000	0.000200000
6	-2.492299000	1.026466000	-0.000019000
6	-3.247499000	2.234272000	-0.000017000
7	-3.171994000	-0.142458000	0.000027000
6	-4.611419000	2.157196000	0.000032000
6	-5.207417000	0.868298000	0.000083000
7	-4.502827000	-0.237413000	0.000088000
9	-2.753326000	-2.217694000	1.154274000
9	-2.754190000	-2.218504000	-1.153127000
1	-2.696578000	3.172489000	-0.000058000
1	-5.235967000	3.049868000	0.000035000
1	-6.288190000	0.735203000	0.000133000
**Total Energy**		-1021.16727741	

Table S10: Cartesian coordinates and total energy in a.u. of the ground state optimized geometry of the $\mathbf{C_6F_5Cl}$ molecule at the MN15/aug-cc-pVDZ level of theory.

Atomic Number	X (Å)	Y (Å)	Z (Å)
17	-2.831155000	-0.000001000	0.000001000
9	-1.054077000	2.363753000	0.000000000
9	3.009542000	0.000001000	-0.000001000
9	1.649918000	2.356678000	-0.000001000
9	-1.054076000	-2.363754000	0.000000000
9	1.649921000	-2.356676000	0.000000000
6	-1.115624000	-0.000001000	0.000001000
6	-0.407460000	1.202142000	0.000000000
6	0.984367000	-1.205323000	0.000000000
6	0.984366000	1.205323000	0.000000000
6	1.681576000	0.000001000	0.000001000
6	-0.407459000	-1.202143000	0.000001000
**Total Energy**		-1187.33482839	

Table S11: Cartesian coordinates and total energy in a.u. of the ground state optimized geometry of the $\mathbf{C_6F_5Br}$ molecule at the MN15/aug-cc-pVDZ-PP level of theory.

Atomic Number	X (Å)	Y (Å)	Z (Å)
35	2.503207000	0.000000000	-0.000017000
9	0.561519000	-2.365752000	-0.000051000
9	-3.495839000	0.000000000	0.000024000
9	-2.136261000	-2.356648000	-0.000033000
9	0.561518000	2.365752000	0.000044000
9	-2.136261000	2.356648000	0.000062000
6	0.632539000	0.000000000	-0.000004000

6	-0.078811000	-1.199045000	-0.000023000
6	-1.470548000	1.205091000	0.000034000
6	-1.470548000	-1.205091000	-0.000014000
6	-2.167876000	0.000000000	0.000015000
6	-0.078811000	1.199045000	0.000025000
**Total Energy**		-1144.50413300	

Table S12: Cartesian coordinates and total energy in a.u. of the ground state optimized geometry of the $\mathbf{C_6F_5I}$ molecule at the MN15/aug-cc-pVDZ-PP level of theory.

**Atomic Number**	**X (Å)**	**Y (Å)**	**Z (Å)**
53	-2.297884000	-0.000013000	0.000001000
9	-0.135465000	2.368090000	-0.000003000
9	3.916166000	0.000079000	0.000004000
9	2.557902000	2.357097000	0.000002000
9	-0.135273000	-2.368222000	-0.000003000
9	2.558068000	-2.356995000	0.000001000
6	-0.215802000	-0.000076000	-0.000005000
6	0.499792000	1.196518000	-0.000004000
6	1.892027000	-1.205858000	-0.000001000
6	1.891928000	1.205965000	-0.000001000
6	2.588066000	0.000057000	0.000000000
6	0.499871000	-1.196571000	-0.000004000
**Total Energy**		-1022.20939602	

Table S13: Cartesian coordinates and total energy in a.u. of the ground state optimized geometry of **13PN**:C_6F_5Cl complex with the halogen bond at the *heterocyclic* nitrogen at the MN15/aug-cc-pVDZ level of theory.

Atomic Number	X (Å)	Y (Å)	Z (Å)
6	3.196386000	2.569842000	-0.059226000
6	2.319487000	1.471554000	-0.036126000
6	2.713203000	3.868118000	-0.063340000
1	3.422261000	4.692591000	-0.079910000
6	0.934484000	1.715757000	-0.017264000
6	1.317153000	4.121258000	-0.043680000
1	0.252938000	0.866253000	-0.000593000
6	0.437641000	3.007288000	-0.020293000
1	-0.639399000	3.156818000	-0.005099000
1	4.270299000	2.390665000	-0.071729000
7	0.831873000	5.402582000	-0.046774000
6	-0.598223000	5.636666000	-0.023363000
1	-1.093598000	5.198749000	-0.903226000
1	-1.062176000	5.212375000	0.879992000
1	-0.780925000	6.714784000	-0.028410000
6	1.747717000	6.525058000	-0.070177000
1	1.170700000	7.453909000	-0.070060000
1	2.405937000	6.528016000	0.812132000
1	2.379504000	6.512003000	-0.971475000
6	2.830811000	0.106495000	-0.029541000
8	4.134652000	-0.016206000	-0.097422000
7	1.977293000	-0.901941000	0.019339000
5	4.835978000	-1.279501000	0.145661000
6	2.437577000	-2.167202000	-0.035558000
7	1.526584000	-3.165962000	-0.095299000

7	3.773823000	-2.438951000	-0.038756000
6	1.968335000	-4.409096000	-0.172132000
6	3.333047000	-4.755232000	-0.200102000
6	4.220610000	-3.707386000	-0.124096000
9	5.308673000	-1.348927000	1.448521000
9	5.843966000	-1.474809000	-0.783484000
1	5.304271000	-3.821519000	-0.125733000
1	3.667735000	-5.786575000	-0.268802000
1	1.204136000	-5.189004000	-0.215337000
9	-2.392356000	1.196944000	0.018550000
9	-6.941696000	0.014785000	0.047461000
9	-4.992719000	1.918000000	0.046386000
9	-3.667167000	-3.357619000	-0.007844000
9	-6.266186000	-2.621896000	0.020241000
6	-2.961915000	-1.100333000	0.005209000
6	-3.326739000	0.246151000	0.019137000
6	-5.314392000	-1.692177000	0.019961000
6	-4.664665000	0.627616000	0.033404000
6	-5.661730000	-0.343905000	0.033871000
6	-3.972573000	-2.062955000	0.005610000
17	-1.311512000	-1.562084000	-0.012500000
**Total Energy**		-2208.54531459	

Table S14: Cartesian coordinates and total energy in a.u. of the ground state optimized geometry of **13PN**:C_6F_5Br complex with the halogen bond at the *heterocyclic* nitrogen at the MN15/aug-cc-pVDZ-PP level of theory.

Atomic Number	X (Å)	Y (Å)	Z (Å)

6	3.399537000	2.582767000	-0.060726000
6	2.482969000	1.517277000	-0.034050000
6	2.962689000	3.896974000	-0.065367000
1	3.700223000	4.695983000	-0.084662000
6	1.106762000	1.810238000	-0.012603000
6	1.575926000	4.199755000	-0.042932000
1	0.395428000	0.985246000	0.005895000
6	0.656468000	3.118004000	-0.016413000
1	-0.414923000	3.304488000	0.000273000
1	4.466323000	2.364818000	-0.075492000
7	1.137166000	5.496911000	-0.046503000
6	-0.283960000	5.782241000	-0.020690000
1	-0.795996000	5.362139000	-0.899558000
1	-0.760791000	5.374839000	0.883559000
1	-0.427747000	6.866190000	-0.025534000
6	2.092341000	6.586271000	-0.074442000
1	1.548617000	7.534936000	-0.074469000
1	2.752440000	6.567292000	0.806172000
1	2.720587000	6.549123000	-0.977467000
6	2.939598000	0.135174000	-0.026476000
8	4.235233000	-0.049049000	-0.099660000
7	2.041810000	-0.835225000	0.027784000
5	4.878030000	-1.344866000	0.142518000
6	2.441538000	-2.117550000	-0.027820000
7	1.480141000	-3.069525000	-0.081766000
7	3.761747000	-2.455622000	-0.037755000

6	1.856283000	-4.334250000	-0.159312000
6	3.201857000	-4.747079000	-0.193743000
6	4.142304000	-3.745135000	-0.123987000
9	5.348533000	-1.435354000	1.444156000
9	5.871737000	-1.588252000	-0.789530000
1	5.218623000	-3.915021000	-0.131471000
1	3.483690000	-5.793982000	-0.262850000
1	1.053070000	-5.073885000	-0.197769000
9	-2.208914000	1.310674000	0.021076000
9	-6.836460000	0.517385000	0.051792000
9	-4.731015000	2.246393000	0.052192000
9	-3.869855000	-3.122537000	-0.011438000
9	-6.388811000	-2.168269000	0.019681000
6	-2.960462000	-0.937153000	0.004305000
6	-3.215884000	0.433245000	0.020821000
6	-5.360813000	-1.322693000	0.020168000
6	-4.514707000	0.931947000	0.036891000
6	-5.591484000	0.050009000	0.036621000
6	-4.053989000	-1.802626000	0.004186000
35	-1.202468000	-1.592174000	-0.016920000
**Total Energy**		-2165.71969157	

Table S15: Cartesian coordinates and total energy in a.u. of the ground state optimized geometry of **13PN**:C_6F_5I complex with the halogen bond at the *heterocyclic* nitrogen at the MN15/aug-cc-pVDZ-PP level of theory.

Atomic Number	X (Å)	Y (Å)	Z (Å)
6	3.590023000	2.631949000	-0.066122000

6	2.655257000	1.582161000	-0.029755000
6	3.175790000	3.952992000	-0.071188000
1	3.926587000	4.739268000	-0.097915000
6	1.283496000	1.898763000	0.000518000
6	1.794065000	4.279575000	-0.039896000
1	0.555915000	1.087642000	0.026096000
6	0.855967000	3.213670000	-0.004140000
1	-0.212183000	3.417849000	0.019090000
1	4.652789000	2.395547000	-0.088094000
7	1.377822000	5.583259000	-0.043929000
6	-0.038363000	5.893265000	-0.009081000
1	-0.563345000	5.479702000	-0.883222000
1	-0.515516000	5.496537000	0.899573000
1	-0.163268000	6.979491000	-0.015967000
6	2.351115000	6.656498000	-0.081478000
1	1.823366000	7.614083000	-0.080162000
1	3.016796000	6.628486000	0.794589000
1	2.971992000	6.606380000	-0.988874000
6	3.087284000	0.193994000	-0.022304000
8	4.377093000	-0.019707000	-0.106959000
7	2.170104000	-0.759254000	0.043702000
5	4.994612000	-1.330160000	0.127617000
6	2.540686000	-2.047063000	-0.014592000
7	1.556770000	-2.978662000	-0.056609000
7	3.851606000	-2.417798000	-0.039473000
6	1.899487000	-4.252982000	-0.136972000
6	3.234329000	-4.696151000	-0.185876000

6	4.199258000	-3.716020000	-0.128403000
9	5.476703000	-1.431579000	1.423515000
9	5.970341000	-1.595632000	-0.816420000
1	5.270994000	-3.912218000	-0.148290000
1	3.490533000	-5.749405000	-0.256780000
1	1.077206000	-4.971732000	-0.165750000
53	-1.135745000	-1.630663000	-0.008560000
9	-2.155049000	1.420396000	0.028585000
9	-6.823078000	0.940584000	0.038017000
9	-4.605630000	2.523048000	0.048864000
9	-4.117782000	-2.893985000	-0.014037000
9	-6.561397000	-1.768880000	0.006454000
6	-3.051788000	-0.777278000	0.006857000
6	-3.218176000	0.606448000	0.023104000
6	-5.476640000	-0.996332000	0.011955000
6	-4.478429000	1.196246000	0.033710000
6	-5.612389000	0.389020000	0.028143000
6	-4.204015000	-1.561489000	0.001572000
**Total Energy**		-2043.42822663	

Table S16: Cartesian coordinates and total energy in a.u. of the ground state optimized geometry of **14PN**:C_6F_5Cl complex with the halogen bond at the *heterocyclic* nitrogen at the MN15/aug-cc-pVDZ level of theory.

Atomic Number	X (Å)	Y (Å)	Z (Å)
6	-5.213318000	-0.324045000	-0.107705000
6	-3.810226000	-0.293443000	-0.035901000
6	-5.901837000	-1.526165000	-0.117655000

1	-6.987828000	-1.509146000	-0.172280000
6	-3.113748000	-1.513007000	0.026254000
6	-5.205561000	-2.760450000	-0.054579000
1	-2.026276000	-1.491844000	0.080869000
6	-3.788477000	-2.721809000	0.018284000
1	-3.214946000	-3.644499000	0.068126000
1	-5.761345000	0.615764000	-0.152972000
7	-5.879962000	-3.953283000	-0.062929000
6	-5.147354000	-5.201435000	0.007260000
1	-4.463342000	-5.317120000	-0.847342000
1	-4.557216000	-5.271983000	0.933742000
1	-5.858792000	-6.031696000	-0.009233000
6	-7.327069000	-3.967634000	-0.137451000
1	-7.672108000	-5.005268000	-0.134158000
1	-7.781173000	-3.450726000	0.721752000
1	-7.688505000	-3.487446000	-1.059562000
6	-3.091702000	0.975863000	-0.022207000
8	-3.829446000	2.054034000	-0.145349000
7	-1.774404000	0.979560000	0.078876000
5	-3.336296000	3.404859000	0.151977000
6	-1.125103000	2.163631000	0.014531000
6	0.300043000	2.190758000	-0.037718000
7	-1.761521000	3.355063000	-0.016490000
7	0.991632000	3.304923000	-0.125274000
6	0.312593000	4.477370000	-0.168256000
6	-1.060826000	4.508026000	-0.111821000
9	-3.602295000	3.753913000	1.467328000

9	-3.837078000	4.322746000	-0.751611000
1	0.830017000	1.237345000	-0.005956000
1	-1.648538000	5.423783000	-0.137542000
1	0.892134000	5.395177000	-0.244150000
9	2.462353000	-1.037669000	0.056017000
9	6.448821000	-3.531123000	0.117286000
9	3.729705000	-3.426071000	0.159690000
9	6.643720000	1.159784000	-0.134158000
9	7.898634000	-1.234140000	-0.029545000
6	4.524618000	0.115898000	-0.041358000
6	3.793531000	-1.069928000	0.034224000
6	6.569995000	-1.180423000	-0.008942000
6	4.437716000	-2.302188000	0.087856000
6	5.829105000	-2.357071000	0.066240000
6	5.917690000	0.048408000	-0.062414000
17	3.713506000	1.625657000	-0.106740000
**Total Energy**		-2208.53457448	

Table S17: Cartesian coordinates and total energy in a.u. of the ground state optimized geometry of **14PN**:C_6F_5Br complex with the halogen bond at the *heterocyclic* nitrogen at the MN15/aug-cc-pVDZ-PP level of theory.

Atomic Number	X (Å)	Y (Å)	Z (Å)
6	6.100747000	-0.034584000	-0.135172000
6	4.722537000	0.220337000	-0.027064000
6	7.019551000	1.001617000	-0.146987000
1	8.077634000	0.764893000	-0.230676000
6	4.291540000	1.555260000	0.070158000

6	6.591547000	2.350945000	-0.048530000
1	3.224367000	1.755610000	0.153198000
6	5.198228000	2.600808000	0.061368000
1	4.826748000	3.619939000	0.139105000
1	6.444140000	-1.065372000	-0.207714000
7	7.494400000	3.380600000	-0.058492000
6	7.033937000	4.750769000	0.046927000
1	6.368777000	5.018548000	-0.788099000
1	6.492207000	4.923195000	0.989359000
1	7.899424000	5.418575000	0.022715000
6	8.912206000	3.101556000	-0.171543000
1	9.461414000	4.047035000	-0.162850000
1	9.270963000	2.486886000	0.668026000
1	9.146353000	2.575826000	-1.109682000
6	3.760829000	-0.874332000	-0.012626000
8	4.257318000	-2.078831000	-0.164226000
7	2.471156000	-0.609599000	0.116423000
5	3.503138000	-3.305334000	0.125687000
6	1.593244000	-1.632993000	0.051077000
6	0.191660000	-1.367073000	0.028690000
7	1.968333000	-2.930170000	-0.009198000
7	-0.712542000	-2.316141000	-0.058441000
6	-0.294073000	-3.603473000	-0.130892000
6	1.044931000	-3.913705000	-0.104355000
9	3.713252000	-3.723157000	1.430327000
9	3.787101000	-4.289971000	-0.800480000
1	-0.136185000	-0.327651000	0.083361000

1	1.432026000	-4.929690000	-0.154643000
1	-1.052518000	-4.379631000	-0.205924000
9	-3.340403000	2.199874000	0.102418000
9	-7.996958000	2.808021000	0.039656000
9	-5.480095000	3.840361000	0.139239000
9	-6.228777000	-1.539330000	-0.135124000
9	-8.358818000	0.113770000	-0.097253000
6	-4.722914000	0.282304000	-0.017344000
6	-4.560795000	1.665030000	0.052680000
6	-7.128187000	0.617899000	-0.048657000
6	-5.656755000	2.522865000	0.072333000
6	-6.944460000	1.996113000	0.021453000
6	-6.020355000	-0.224879000	-0.067434000
35	-3.235350000	-0.862759000	-0.043325000
**Total Energy**		-2165.70727321	

Table S18: Cartesian coordinates and total energy in a.u. of the ground state optimized geometry of **14PN**:C_6F_5I complex with the halogen bond at the *heterocyclic* nitrogen at the MN15/aug-cc-pVDZ-PP level of theory.

**Atomic Number**	**X (Å)**	**Y (Å)**	**Z (Å)**
6	6.721624000	-0.300773000	-0.161499000
6	5.402431000	0.167962000	-0.030302000
6	7.792110000	0.577040000	-0.183540000
1	8.798008000	0.176689000	-0.284900000
6	5.189362000	1.553789000	0.079751000
6	7.584230000	1.976599000	-0.072339000
1	4.168790000	1.919863000	0.180747000

6	6.249475000	2.442829000	0.060713000
1	6.045215000	3.507245000	0.148897000
1	6.896827000	-1.372255000	-0.243778000
7	8.637802000	2.850269000	-0.092232000
6	8.401760000	4.275573000	0.026041000
1	7.775023000	4.650543000	-0.797290000
1	7.908665000	4.525242000	0.977740000
1	9.361647000	4.797935000	-0.009364000
6	9.992028000	2.351583000	-0.230037000
1	10.683686000	3.198413000	-0.226386000
1	10.262137000	1.682370000	0.600857000
1	10.125154000	1.802081000	-1.174217000
6	4.281950000	-0.761506000	-0.005432000
8	4.579406000	-2.027665000	-0.172386000
7	3.050442000	-0.299116000	0.146461000
5	3.648705000	-3.121558000	0.134450000
6	2.023513000	-1.171333000	0.087657000
6	0.680560000	-0.688605000	0.085653000
7	2.188535000	-2.511613000	0.015105000
7	-0.358069000	-1.487994000	0.003967000
6	-0.151722000	-2.824975000	-0.082839000
6	1.122960000	-3.339353000	-0.074949000
9	3.806098000	-3.558711000	1.439608000
9	3.763349000	-4.141800000	-0.788447000
1	0.518056000	0.387744000	0.151672000
1	1.347148000	-4.402598000	-0.136298000
1	-1.027059000	-3.467803000	-0.153170000

53	-3.032466000	-0.383320000	-0.004303000
9	-4.141460000	2.638248000	0.198427000
9	-8.789325000	2.026475000	-0.026970000
9	-6.628968000	3.668159000	0.187697000
9	-5.961545000	-1.714177000	-0.222823000
9	-8.441028000	-0.665224000	-0.231666000
6	-4.969668000	0.427752000	-0.011696000
6	-5.177600000	1.801773000	0.091673000
6	-7.382958000	0.136407000	-0.126676000
6	-6.456663000	2.351475000	0.087729000
6	-7.562496000	1.512946000	-0.022044000
6	-6.093638000	-0.388840000	-0.120396000
**Total Energy**		-2043.41551065	

Table S19: Cartesian coordinates and total energy in a.u. of the ground state optimized geometry of **15PN**:C_6F_5Cl complex with the halogen bond at the *heterocyclic* nitrogen at the MN15/aug-cc-pVDZ level of theory.

**Atomic Number**	**X (Å)**	**Y (Å)**	**Z (Å)**
6	-6.294978000	0.744558000	-0.151828000
6	-5.149764000	-0.060075000	-0.021007000
6	-7.561513000	0.185033000	-0.180511000
1	-8.423359000	0.840359000	-0.281792000
6	-5.315348000	-1.452338000	0.081819000
6	-7.735684000	-1.219254000	-0.076538000
1	-4.429443000	-2.077375000	0.182754000
6	-6.574950000	-2.025757000	0.056053000
1	-6.662962000	-3.106511000	0.138816000

1	-6.176610000	1.824137000	-0.229264000
7	-8.985378000	-1.779483000	-0.102824000
6	-9.139150000	-3.216230000	0.007053000
1	-8.633028000	-3.740884000	-0.817643000
1	-8.733358000	-3.594278000	0.957787000
1	-10.203659000	-3.463005000	-0.032706000
6	-10.155967000	-0.935859000	-0.239566000
1	-11.049304000	-1.566431000	-0.240665000
1	-10.238976000	-0.222202000	0.594194000
1	-10.135188000	-0.366274000	-1.181100000
6	-3.819854000	0.537307000	0.010127000
8	-3.773983000	1.836168000	-0.141650000
7	-2.760276000	-0.243560000	0.157294000
5	-2.572867000	2.642927000	0.129286000
6	-1.531036000	0.311395000	0.117284000
6	-0.368732000	-0.494554000	0.139175000
7	-1.339925000	1.657648000	0.043393000
6	0.858803000	0.122976000	0.075903000
7	1.013904000	1.470320000	-0.009240000
6	-0.092840000	2.174318000	-0.018859000
1	-0.043561000	3.260927000	-0.082840000
9	-2.612457000	3.163839000	1.413712000
9	-2.417711000	3.623022000	-0.833066000
1	-0.479686000	-1.573989000	0.200890000
1	1.779753000	-0.463565000	0.089445000
9	4.213472000	-1.783250000	0.082765000
9	8.886905000	-2.302031000	0.007786000

9	6.392619000	-3.386220000	0.121088000
9	7.022757000	2.009992000	-0.183765000
9	9.193065000	0.396258000	-0.144065000
6	5.568646000	0.151117000	-0.051522000
6	5.427158000	-1.234473000	0.026435000
6	7.973698000	-0.131104000	-0.089019000
6	6.542027000	-2.066565000	0.046733000
6	7.818540000	-1.512789000	-0.011196000
6	6.852268000	0.693992000	-0.108999000
17	4.180678000	1.157998000	-0.075340000
**Total Energy**		-2208.54581052	

Table S20: Cartesian coordinates and total energy in a.u. of the ground state optimized geometry of **15PN**:C_6F_5Br complex with the halogen bond at the *heterocyclic* nitrogen at the MN15/aug-cc-pVDZ-PP level of theory.

Atomic Number	X (Å)	Y (Å)	Z (Å)
6	-6.641391000	0.934066000	-0.167843000
6	-5.643609000	-0.046283000	-0.023454000
6	-7.980949000	0.587461000	-0.215608000
1	-8.723733000	1.373823000	-0.326639000
6	-6.034377000	-1.393731000	0.073154000
6	-8.382082000	-0.770493000	-0.118083000
1	-5.263381000	-2.154494000	0.184556000
6	-7.369437000	-1.755060000	0.028371000
1	-7.632923000	-2.807171000	0.106940000
1	-6.348422000	1.980227000	-0.240288000
7	-9.704824000	-1.120291000	-0.163333000

6	-10.091634000	-2.513152000	-0.057643000
1	-9.665600000	-3.113442000	-0.875843000
1	-9.766382000	-2.950901000	0.898339000
1	-11.181348000	-2.583658000	-0.113157000
6	-10.721608000	-0.097813000	-0.312797000
1	-11.705008000	-0.575536000	-0.328835000
1	-10.699824000	0.618230000	0.522670000
1	-10.595302000	0.462109000	-1.251748000
6	-4.236471000	0.326431000	0.028125000
8	-3.974627000	1.599355000	-0.120384000
7	-3.319452000	-0.618234000	0.189060000
5	-2.663755000	2.197035000	0.182381000
6	-2.017657000	-0.273549000	0.164571000
6	-1.003395000	-1.261081000	0.196398000
7	-1.605842000	1.023971000	0.096792000
6	0.307994000	-0.854190000	0.146436000
7	0.681434000	0.450600000	0.065221000
6	-0.292091000	1.329956000	0.047133000
1	-0.062193000	2.393140000	-0.013659000
9	-2.639721000	2.694639000	1.475630000
9	-2.333655000	3.151711000	-0.759909000
1	-1.291375000	-2.307449000	0.254465000
1	1.123481000	-1.580097000	0.167699000
9	4.982796000	-2.430726000	0.102634000
9	9.452274000	-1.001976000	-0.100639000
9	7.616774000	-3.006104000	0.061137000
9	5.999561000	2.180962000	-0.179458000

9	8.630636000	1.589923000	-0.220441000
6	5.415231000	-0.107300000	-0.037217000
6	5.858470000	-1.427462000	0.023083000
6	7.732846000	0.610935000	-0.141385000
6	7.214880000	-1.738487000	0.002441000
6	8.154446000	-0.714469000	-0.080174000
6	6.371822000	0.903255000	-0.119487000
35	3.581276000	0.293509000	-0.007835000
**Total Energy**		-2165.71865908	

Table S21: Cartesian coordinates and total energy in a.u. of the ground state optimized geometry of **15PN**:C_6F_5I complex with the halogen bond at the *heterocyclic* nitrogen at the MN15/aug-cc-pVDZ-PP level of theory.

**Atomic Number**	**X (Å)**	**Y (Å)**	**Z (Å)**
6	-6.893181000	1.019754000	-0.193103000
6	-5.973228000	-0.031266000	-0.026620000
6	-8.254063000	0.774396000	-0.250348000
1	-8.934432000	1.612839000	-0.378527000
6	-6.465350000	-1.344528000	0.082272000
6	-8.757430000	-0.548522000	-0.140300000
1	-5.754981000	-2.159685000	0.210616000
6	-7.823098000	-1.604839000	0.028403000
1	-8.166043000	-2.632948000	0.117033000
1	-6.521578000	2.039905000	-0.275052000
7	-10.101625000	-0.798068000	-0.194615000
6	-10.593755000	-2.156572000	-0.076499000
1	-10.207127000	-2.797182000	-0.883538000

1	-10.310922000	-2.605479000	0.887660000
1	-11.685102000	-2.145111000	-0.141687000
6	-11.037289000	0.295981000	-0.367891000
1	-12.053572000	-0.106767000	-0.388222000
1	-10.969607000	1.019332000	0.458667000
1	-10.860059000	0.831905000	-1.312515000
6	-4.544175000	0.236348000	0.035245000
8	-4.185382000	1.483522000	-0.126385000
7	-3.700207000	-0.772689000	0.216715000
5	-2.839774000	1.984559000	0.198298000
6	-2.377918000	-0.527003000	0.197874000
6	-1.439712000	-1.588153000	0.244013000
7	-1.869133000	0.736302000	0.121451000
6	-0.102079000	-1.282106000	0.196347000
7	0.365637000	-0.008040000	0.104730000
6	-0.537614000	0.943674000	0.074816000
1	-0.226385000	1.985587000	0.005913000
9	-2.798331000	2.470737000	1.494845000
9	-2.426459000	2.915022000	-0.734093000
1	-1.805551000	-2.609355000	0.310085000
1	0.658193000	-2.065161000	0.227667000
53	3.273193000	0.027063000	0.014470000
9	5.276808000	-2.499309000	0.031249000
9	9.493251000	-0.446082000	-0.149861000
9	7.960622000	-2.693032000	-0.040191000
9	5.631752000	2.221442000	-0.117665000
9	8.314827000	2.006920000	-0.188179000

6	5.365592000	-0.131594000	-0.040944000
6	5.996015000	-1.374230000	-0.022457000
6	7.563419000	0.909052000	-0.134451000
6	7.382527000	-1.493319000	-0.058732000
6	8.167488000	-0.345023000	-0.114902000
6	6.174674000	1.001781000	-0.097447000
**Total Energy**		-2043.42671345	

Table S22: Cartesian coordinates and total energy in a.u. of the ground state optimized geometry of **16PN**:C_6F_5Cl complex with the halogen bond at the *heterocyclic* nitrogen at the MN15/aug-cc-pVDZ level of theory.

**Atomic Number**	**X (Å)**	**Y (Å)**	**Z (Å)**
6	5.562782000	-1.246176000	-0.001364000
6	5.005255000	0.043474000	-0.000818000
6	6.935940000	-1.430964000	-0.001912000
1	7.329317000	-2.444795000	-0.002311000
6	5.872075000	1.149177000	-0.000839000
6	7.816717000	-0.319332000	-0.001924000
1	5.442748000	2.149927000	-0.000419000
6	7.246755000	0.980214000	-0.001370000
1	7.885294000	1.860610000	-0.001358000
1	4.898634000	-2.109047000	-0.001329000
7	9.176365000	-0.494967000	-0.002450000
6	10.056195000	0.655857000	-0.002417000
1	9.902265000	1.282566000	-0.894269000
1	9.902856000	1.282062000	0.889891000
1	11.093421000	0.309454000	-0.002857000

6	9.734499000	-1.832172000	-0.003003000
1	10.825739000	-1.761023000	-0.003386000
1	9.426595000	-2.398957000	0.888942000
1	9.425941000	-2.398487000	-0.895020000
6	3.555531000	0.227766000	-0.000202000
8	2.843569000	-0.863056000	-0.000718000
7	3.059132000	1.454975000	0.000472000
5	1.362476000	-0.928239000	0.002156000
6	1.716382000	1.613711000	0.000471000
6	1.167934000	2.928173000	0.000076000
7	0.854909000	0.572480000	0.000797000
6	-0.190495000	3.074885000	0.000012000
6	-0.990499000	1.902001000	0.000361000
7	-0.472687000	0.697109000	0.000778000
9	0.921541000	-1.541273000	1.156821000
9	0.917383000	-1.545077000	-1.148781000
1	1.864047000	3.764344000	-0.000168000
1	-0.660350000	4.057665000	-0.000287000
1	-2.079424000	1.943689000	0.000340000
9	-5.461087000	-2.790820000	0.000590000
9	-9.102610000	0.182258000	-0.000042000
9	-8.129318000	-2.357753000	0.000322000
9	-4.717109000	1.879280000	0.000134000
9	-7.387457000	2.297731000	-0.000134000
6	-5.024364000	-0.467693000	0.000370000
6	-5.914609000	-1.542202000	0.000415000
6	-6.912552000	1.054493000	0.000044000

6	-7.290189000	-1.326103000	0.000277000
6	-7.790211000	-0.026519000	0.000091000
6	-5.539905000	0.828572000	0.000183000
17	-3.330445000	-0.724802000	0.000536000
**Total Energy**		-2208.50729315	

Table S23: Cartesian coordinates and total energy in a.u. of the ground state optimized geometry of **16PN**:C_6F_5Br complex with the halogen bond at the *heterocyclic* nitrogen at the MN15/aug-cc-pVDZ-PP level of theory.

Atomic Number	X (Å)	Y (Å)	Z (Å)
6	5.067617000	-1.379947000	-0.003386000
6	4.855295000	0.009406000	-0.000582000
6	6.348581000	-1.906692000	-0.002884000
1	6.472210000	-2.987055000	-0.005115000
6	5.974461000	0.859523000	0.002770000
6	7.482721000	-1.054392000	0.000514000
1	5.813255000	1.936438000	0.004937000
6	7.260962000	0.347560000	0.003329000
1	8.101911000	1.037134000	0.005949000
1	4.206608000	-2.046416000	-0.005958000
7	8.752557000	-1.568719000	0.001068000
6	9.895931000	-0.678699000	0.004490000
1	9.907283000	-0.032101000	-0.886268000
1	9.904901000	-0.035924000	0.898033000
1	10.811154000	-1.277063000	0.004437000
6	8.953846000	-3.004035000	-0.001962000
1	10.027472000	-3.211486000	-0.000964000

1	8.511001000	-3.475735000	0.888412000
1	8.513442000	-3.471638000	-0.895705000
6	3.501100000	0.554327000	-0.001090000
8	2.535190000	-0.320744000	-0.004974000
7	3.331267000	1.868762000	0.001999000
5	1.088317000	-0.009996000	-0.002179000
6	2.074232000	2.361737000	0.001047000
6	1.869937000	3.772516000	0.002608000
7	0.979253000	1.570299000	-0.001377000
6	0.591444000	4.254744000	0.001713000
6	-0.477066000	3.319138000	-0.000686000
7	-0.272570000	2.024963000	-0.002125000
9	0.498285000	-0.486513000	1.150541000
9	0.494312000	-0.486043000	-1.152924000
1	2.752139000	4.409178000	0.004493000
1	0.383142000	5.323867000	0.002847000
1	-1.522190000	3.627028000	-0.001394000
9	-3.671106000	-2.505547000	0.001370000
9	-8.318696000	-1.836174000	0.002561000
9	-6.171447000	-3.509456000	0.003162000
9	-5.447550000	1.880226000	-0.001628000
9	-7.941237000	0.861074000	0.000164000
6	-4.481426000	-0.282580000	-0.000185000
6	-4.699002000	-1.660236000	0.001052000
6	-6.890921000	0.041325000	0.000454000
6	-5.986294000	-2.190937000	0.001985000
6	-7.085278000	-1.336853000	0.001682000

6	-5.597062000	0.553851000	-0.000462000
35	-2.745043000	0.423312000	-0.001475000
**Total Energy**		-2165.68072472	

Table S24: Cartesian coordinates and total energy in a.u. of the ground state optimized geometry of **16PN**:C_6F_5I complex with the halogen bond at the *heterocyclic* nitrogen at the MN15/aug-cc-pVDZ-PP level of theory.

**Atomic Number**	**X (Å)**	**Y (Å)**	**Z (Å)**
6	5.377226000	-1.405065000	-0.002649000
6	5.153498000	-0.017236000	-0.000305000
6	6.662229000	-1.921194000	-0.002189000
1	6.794764000	-3.000479000	-0.004031000
6	6.265798000	0.842249000	0.002544000
6	7.789486000	-1.059439000	0.000678000
1	6.095956000	1.917825000	0.004369000
6	7.556223000	0.340875000	0.003052000
1	8.391533000	1.037228000	0.005295000
1	4.521842000	-2.078719000	-0.004818000
7	9.063045000	-1.563185000	0.001146000
6	10.199364000	-0.663879000	0.004257000
1	10.205251000	-0.017530000	-0.886663000
1	10.203169000	-0.021038000	0.897726000
1	11.119308000	-1.254897000	0.004161000
6	9.276276000	-2.996972000	-0.001209000
1	10.351564000	-3.195429000	-0.000442000
1	8.837637000	-3.471703000	0.889571000
1	8.839523000	-3.468464000	-0.894629000

6	3.795749000	0.516201000	-0.000766000
8	2.836377000	-0.366720000	-0.004173000
7	3.614215000	1.829584000	0.001758000
5	1.388947000	-0.067865000	-0.001763000
6	2.353852000	2.311821000	0.000756000
6	2.137167000	3.721314000	0.001872000
7	1.265615000	1.511084000	-0.001280000
6	0.855010000	4.192978000	0.000914000
6	-0.206251000	3.248779000	-0.001081000
7	0.010363000	1.956675000	-0.002108000
9	0.799181000	-0.547377000	1.150423000
9	0.795682000	-0.547256000	-1.152096000
1	3.014001000	4.365367000	0.003471000
1	0.637473000	5.260161000	0.001702000
1	-1.254526000	3.546770000	-0.001826000
53	-2.545958000	0.362759000	-0.001538000
9	-3.808783000	-2.600727000	0.000153000
9	-8.420254000	-1.731617000	0.002848000
9	-6.346772000	-3.494826000	0.002046000
9	-5.401358000	1.861310000	-0.000158000
9	-7.930713000	0.946809000	0.001734000
6	-4.517501000	-0.339825000	-0.000064000
6	-4.797659000	-1.705767000	0.000520000
6	-6.914821000	0.084146000	0.001342000
6	-6.105129000	-2.184952000	0.001501000
6	-7.166643000	-1.284705000	0.001912000
6	-5.599377000	0.539305000	0.000363000

**Total Energy**	-2043.38898030

Table S25: Cartesian coordinates and total energy in a.u. of the ground state optimized geometry of **PN**:C_6F_5Cl complex with the halogen bond at the *imine* nitrogen at the MN15/aug-cc-pVDZ level of theory.

Atomic Number	X (Å)	Y (Å)	Z (Å)
6	4.293149000	0.392789000	0.496091000
6	3.079516000	0.148179000	-0.166466000
6	4.904614000	1.635553000	0.434009000
1	5.843046000	1.786000000	0.962651000
6	2.494713000	1.193674000	-0.900672000
6	4.317696000	2.695688000	-0.301592000
1	1.557470000	1.007508000	-1.423887000
6	3.092885000	2.441065000	-0.970790000
1	2.608328000	3.224906000	-1.548356000
1	4.753563000	-0.410814000	1.068529000
7	4.915152000	3.928987000	-0.365430000
6	4.293119000	4.997380000	-1.119964000
1	4.191871000	4.735153000	-2.184383000
1	3.293393000	5.240489000	-0.728304000
1	4.916279000	5.893056000	-1.046447000
6	6.165574000	4.164503000	0.326830000
1	6.476679000	5.198843000	0.155541000
1	6.062390000	4.011088000	1.412073000
1	6.962563000	3.499961000	-0.040684000
6	2.436033000	-1.163169000	-0.087888000
8	3.100464000	-2.084971000	0.568025000

7	1.273729000	-1.350651000	-0.676151000
5	2.489574000	-3.345643000	1.006918000
6	0.699280000	-2.584360000	-0.639353000
6	-0.482822000	-2.826899000	-1.376581000
7	1.231370000	-3.601611000	0.084454000
6	-1.066825000	-4.076232000	-1.349425000
6	-0.482297000	-5.112287000	-0.593017000
6	0.667180000	-4.831140000	0.109638000
9	2.037617000	-3.247259000	2.317091000
9	3.372522000	-4.399460000	0.840171000
1	1.189645000	-5.563523000	0.723028000
1	-0.917292000	-6.108108000	-0.553584000
1	-1.981276000	-4.260216000	-1.913848000
1	-0.898451000	-2.000676000	-1.948640000
9	-3.558710000	-1.199316000	-0.921476000
9	-6.870007000	1.850215000	0.438052000
9	-6.168523000	-0.549303000	-0.639419000
9	-2.331638000	2.962022000	0.959054000
9	-4.944914000	3.600092000	1.233765000
6	-2.883495000	0.865461000	0.016107000
6	-3.881732000	-0.021189000	-0.388934000
6	-4.600440000	2.427349000	0.709343000
6	-5.226929000	0.305555000	-0.248450000
6	-5.586979000	1.532828000	0.302497000
6	-3.256780000	2.092141000	0.565361000
17	-1.231262000	0.441352000	-0.162246000
**Total Energy**		-2192.51278413	

Table S26: Cartesian coordinates and total energy in a.u. of the ground state optimized geometry of **PN**:C_6F_5Br complex with the halogen bond at the *imine* nitrogen at the MN15/aug-cc-pVDZ-PP level of theory.

Atomic Number	X (Å)	Y (Å)	Z (Å)
6	2.928009000	2.458159000	0.729660000
6	2.334540000	1.511611000	-0.122675000
6	2.532825000	3.786121000	0.708970000
1	3.010243000	4.486835000	1.389909000
6	1.340938000	1.946193000	-1.016958000
6	1.517319000	4.226210000	-0.178120000
1	0.892438000	1.225551000	-1.698812000
6	0.935470000	3.269469000	-1.049667000
1	0.161561000	3.564205000	-1.754327000
1	3.704568000	2.133516000	1.420400000
7	1.112974000	5.535679000	-0.196775000
6	0.063450000	5.957109000	-1.102606000
1	0.349217000	5.798315000	-2.153681000
1	-0.874693000	5.413404000	-0.913760000
1	-0.123339000	7.024635000	-0.956513000
6	1.723518000	6.495565000	0.700388000
1	1.270319000	7.476467000	0.532124000
1	1.567539000	6.219915000	1.754709000
1	2.806380000	6.581692000	0.522996000
6	2.739718000	0.109763000	-0.070161000
8	3.809589000	-0.149966000	0.641691000
7	2.047963000	-0.796899000	-0.735097000
5	4.154277000	-1.510645000	1.076119000

6	2.479664000	-2.089779000	-0.747034000
6	1.843453000	-3.030177000	-1.588489000
7	3.515198000	-2.504546000	0.024620000
6	2.290804000	-4.335197000	-1.621612000
6	3.379479000	-4.728702000	-0.818125000
6	3.961129000	-3.781244000	-0.006256000
9	3.591816000	-1.790361000	2.314141000
9	5.525672000	-1.690782000	1.068220000
1	4.801172000	-3.990193000	0.654088000
1	3.755580000	-5.748936000	-0.823714000
1	1.801816000	-5.061009000	-2.271645000
1	1.010419000	-2.681515000	-2.195603000
9	-2.832259000	-3.059732000	0.243101000
9	-6.763440000	-0.523915000	0.664399000
9	-5.487927000	-2.928396000	0.696287000
9	-2.703590000	1.640934000	-0.280569000
9	-5.359797000	1.755918000	0.173191000
6	-2.690707000	-0.713123000	-0.027381000
6	-3.429047000	-1.868093000	0.224323000
6	-4.734614000	0.580560000	0.191294000
6	-4.800270000	-1.813967000	0.458709000
6	-5.453964000	-0.584841000	0.442277000
6	-3.364287000	0.507671000	-0.040976000
35	-0.840946000	-0.782354000	-0.342052000
**Total Energy**		-2149.68665572	

Table S27: Cartesian coordinates and total energy in a.u. of the ground state optimized geometry of **PN**:C_6F_5I complex with the halogen bond at the *imine* nitrogen at the MN15/aug-cc-pVDZ-PP level of theory.

Atomic Number	X (Å)	Y (Å)	Z (Å)
6	3.472182000	1.990523000	0.808975000
6	2.756129000	1.179382000	-0.088026000
6	3.362214000	3.370755000	0.768695000
1	3.924167000	3.963581000	1.486503000
6	1.938129000	1.801863000	-1.047072000
6	2.525963000	4.003517000	-0.186841000
1	1.399974000	1.183852000	-1.764570000
6	1.821239000	3.179901000	-1.102841000
1	1.180229000	3.622565000	-1.861353000
1	4.115078000	1.518272000	1.550137000
7	2.406204000	5.367462000	-0.228263000
6	1.534565000	5.989682000	-1.204692000
1	1.854779000	5.764646000	-2.233441000
1	0.492700000	5.655765000	-1.085709000
1	1.564873000	7.074008000	-1.066750000
6	3.138211000	6.188662000	0.714977000
1	2.912823000	7.240536000	0.518848000
1	2.853129000	5.962791000	1.753924000
1	4.224922000	6.045296000	0.616019000
6	2.869375000	-0.273631000	-0.021452000
8	3.849418000	-0.739021000	0.713634000
7	2.028032000	-1.031361000	-0.703658000
5	3.863908000	-2.133168000	1.181902000

6	2.222568000	-2.383119000	-0.733848000
6	1.475312000	-3.177597000	-1.630891000
7	3.128780000	-2.988766000	0.072539000
6	1.688849000	-4.540682000	-1.680799000
6	2.649664000	-5.135601000	-0.840153000
6	3.346984000	-4.322396000	0.024943000
9	3.140979000	-2.262406000	2.359100000
9	5.161992000	-2.591570000	1.299225000
1	4.103946000	-4.689060000	0.716045000
1	2.841283000	-6.205798000	-0.858220000
1	1.114377000	-5.155237000	-2.374081000
1	0.749813000	-2.676363000	-2.268443000
53	-0.874073000	-0.502382000	-0.330113000
9	-3.327337000	-2.557698000	0.090421000
9	-6.972107000	0.339755000	0.678675000
9	-5.956227000	-2.180922000	0.528477000
9	-2.698583000	2.135478000	-0.054055000
9	-5.330017000	2.490898000	0.384477000
6	-2.924656000	-0.223409000	0.007077000
6	-3.788664000	-1.306262000	0.159957000
6	-4.831051000	1.257928000	0.312955000
6	-5.151006000	-1.129754000	0.386521000
6	-5.672049000	0.158855000	0.463259000
6	-3.472371000	1.055757000	0.087139000
**Total Energy**		-2027.39483698	

Table S28: Cartesian coordinates and total energy in a.u. of the ground state optimized geometry of **13PN**:C_6F_5Cl complex with the halogen bond at the *imine* nitrogen at the MN15/aug-cc-pVDZ level of theory.

Atomic Number	X (Å)	Y (Å)	Z (Å)
6	-0.122244000	-2.072494000	-0.039318000
6	0.531995000	-1.032297000	-0.721172000
6	-1.466645000	-2.331178000	-0.253189000
1	-1.941582000	-3.139502000	0.297894000
6	-0.198793000	-0.270071000	-1.648986000
6	-2.218102000	-1.548780000	-1.168676000
1	0.312785000	0.530654000	-2.182365000
6	-1.542546000	-0.519988000	-1.876632000
1	-2.076288000	0.090358000	-2.602070000
1	0.440272000	-2.675376000	0.671405000
7	-3.558825000	-1.766091000	-1.347371000
6	-4.302995000	-0.970262000	-2.303032000
1	-4.262459000	0.101906000	-2.057117000
1	-3.919847000	-1.101471000	-3.326244000
1	-5.350599000	-1.281477000	-2.282947000
6	-4.234249000	-2.807349000	-0.596041000
1	-5.298972000	-2.785944000	-0.842962000
1	-3.840207000	-3.805622000	-0.840035000
1	-4.133156000	-2.647971000	0.487233000
6	1.943982000	-0.748491000	-0.475725000
8	2.519171000	-1.484910000	0.444676000
7	2.530641000	0.221726000	-1.150158000
5	3.976055000	-1.547671000	0.594382000

6	3.810079000	0.547888000	-0.867229000
7	4.331547000	1.642677000	-1.464108000
7	4.551816000	-0.196182000	0.002907000
6	5.574588000	1.980421000	-1.165811000
6	6.384721000	1.271074000	-0.258760000
6	5.814922000	0.156583000	0.311424000
9	4.509969000	-2.590066000	-0.149925000
9	4.335581000	-1.617523000	1.928360000
1	6.321584000	-0.495431000	1.022210000
1	7.401643000	1.576744000	-0.028300000
1	5.965069000	2.867862000	-1.670036000
9	-1.997047000	3.340181000	-0.609133000
9	-5.056488000	0.052895000	0.785201000
9	-4.487932000	2.288097000	-0.659699000
9	-0.639804000	-0.041867000	2.401305000
9	-3.128217000	-1.097416000	2.327754000
6	-1.260305000	1.674833000	0.899812000
6	-2.259358000	2.261907000	0.123634000
6	-2.848315000	-0.004186000	1.619545000
6	-3.541434000	1.722681000	0.090455000
6	-3.835374000	0.583949000	0.835200000
6	-1.567218000	0.539902000	1.650456000
17	0.327121000	2.320848000	0.904978000
**Total Energy**		-2208.55722629	

Table S29: Cartesian coordinates and total energy in a.u. of the ground state optimized geometry of **13PN**:C_6F_5Br complex with the halogen bond at the *imine* nitrogen at the MN15/aug-cc-pVDZ-PP level of theory.

Atomic Number	X (Å)	Y (Å)	Z (Å)
6	3.399826000	-2.582296000	0.060235000
6	2.482994000	-1.517034000	0.034126000
6	2.963316000	-3.896611000	0.064667000
1	3.701082000	-4.695399000	0.083434000
6	1.106860000	-1.810336000	0.012951000
6	1.576611000	-4.199747000	0.042698000
1	0.395289000	-0.985554000	-0.005306000
6	0.656884000	-3.118216000	0.016596000
1	-0.414463000	-3.304940000	0.000024000
1	4.466566000	-2.364105000	0.074706000
7	1.138107000	-5.496998000	0.046280000
6	-0.282948000	-5.782590000	0.019594000
1	-0.795524000	-5.362206000	0.897981000
1	-0.759366000	-5.375654000	-0.885108000
1	-0.426544000	-6.866562000	0.024932000
6	2.093503000	-6.586232000	0.071851000
1	1.549918000	-7.534977000	0.071987000
1	2.752345000	-6.566460000	-0.809703000
1	2.723017000	-6.549696000	0.973995000
6	2.939377000	-0.134874000	0.026738000
8	4.235052000	0.049329000	0.098901000
7	2.041515000	0.835478000	-0.026945000
5	4.878224000	1.345344000	-0.140921000

6	2.441260000	2.117817000	0.027730000
7	1.479884000	3.069871000	0.081049000
7	3.761441000	2.455903000	0.037248000
6	1.856060000	4.334638000	0.157297000
6	3.201652000	4.747542000	0.190867000
6	4.142053000	3.745521000	0.122010000
9	5.352158000	1.436720000	-1.441253000
9	5.869655000	1.588341000	0.793726000
1	5.218388000	3.915352000	0.129126000
1	3.483466000	5.794527000	0.258784000
1	1.052863000	5.074320000	0.195304000
9	-2.209670000	-1.311205000	-0.019508000
9	-6.837070000	-0.516983000	-0.051419000
9	-4.731973000	-2.246493000	-0.050541000
9	-3.869705000	3.122340000	0.010334000
9	-6.388895000	2.168511000	-0.020667000
6	-2.960795000	0.936762000	-0.004090000
6	-3.216494000	-0.433613000	-0.019896000
6	-5.361036000	1.322762000	-0.020528000
6	-4.515405000	-0.932090000	-0.035905000
6	-5.591979000	-0.049906000	-0.036287000
6	-4.054133000	1.802469000	-0.004596000
35	-1.202610000	1.591295000	0.017069000
**Total Energy**		-2165.71969166	

Table S30: Cartesian coordinates and total energy in a.u. of the ground state optimized geometry of **13PN**:C_6F_5I complex with the halogen bond at the *imine* nitrogen at the MN15/aug-cc-pVDZ-PP level of theory.

Atomic Number	X (Å)	Y (Å)	Z (Å)
6	-3.384145000	2.220305000	0.782857000
6	-2.679231000	1.344100000	-0.062121000
6	-3.144091000	3.583216000	0.757878000
1	-3.700889000	4.227345000	1.434249000
6	-1.737326000	1.886064000	-0.955712000
6	-2.181638000	4.133536000	-0.129110000
1	-1.207391000	1.219761000	-1.634329000
6	-1.490079000	3.246455000	-0.995273000
1	-0.755267000	3.625801000	-1.701095000
1	-4.122151000	1.811852000	1.471422000
7	-1.931634000	5.478625000	-0.152600000
6	-0.933934000	6.015663000	-1.057370000
1	0.058992000	5.582965000	-0.863858000
1	-1.197196000	5.822840000	-2.108353000
1	-0.870916000	7.097684000	-0.913161000
6	-2.652948000	6.367189000	0.736963000
1	-2.315063000	7.392614000	0.564165000
1	-3.737427000	6.326697000	0.554547000
1	-2.470027000	6.117015000	1.793059000
6	-2.924980000	-0.089276000	-0.009903000
8	-3.981344000	-0.465721000	0.666198000
7	-2.108572000	-0.916163000	-0.648827000
5	-4.210148000	-1.858532000	1.068594000

6	-2.392578000	-2.238675000	-0.688811000
7	-1.619319000	-3.020240000	-1.471818000
7	-3.421077000	-2.761704000	0.033657000
6	-1.905312000	-4.309983000	-1.537562000
6	-2.974795000	-4.909042000	-0.845781000
6	-3.719472000	-4.073544000	-0.045761000
9	-5.550990000	-2.183130000	0.982089000
9	-3.683642000	-2.107075000	2.326268000
1	-4.565409000	-4.397414000	0.559574000
1	-3.193217000	-5.970453000	-0.925069000
1	-1.255264000	-4.914508000	-2.174387000
53	0.892092000	-0.693560000	-0.382338000
9	2.772623000	1.917560000	-0.334566000
9	6.977825000	0.119386000	0.717666000
9	5.398184000	2.260441000	0.139936000
9	3.278618000	-2.744519000	0.344241000
9	5.902421000	-2.379446000	0.816418000
6	2.935984000	-0.426056000	-0.010278000
6	3.514179000	0.841657000	-0.054112000
6	5.128188000	-1.332871000	0.534413000
6	4.870713000	1.037707000	0.188613000
6	5.679690000	-0.055821000	0.484062000
6	3.768557000	-1.503571000	0.287545000
**Total Energy**		-2043.42565269	

Table S31: Cartesian coordinates and total energy in a.u. of the ground state optimized geometry of **14PN**:C_6F_5Cl complex with the halogen bond at the *imine* nitrogen at the MN15/aug-cc-pVDZ level of theory.

Atomic Number	X (Å)	Y (Å)	Z (Å)
6	-2.417302000	2.811440000	0.643291000
6	-1.945969000	1.719432000	-0.106339000
6	-1.739828000	4.019511000	0.649310000
1	-2.134013000	4.839590000	1.245097000
6	-0.770532000	1.881682000	-0.861487000
6	-0.548280000	4.186156000	-0.103031000
1	-0.403986000	1.045032000	-1.453813000
6	-0.082054000	3.082190000	-0.863410000
1	0.824854000	3.165203000	-1.457865000
1	-3.327280000	2.696634000	1.230073000
7	0.131418000	5.375313000	-0.096710000
6	1.349814000	5.517701000	-0.868597000
1	2.116940000	4.797025000	-0.547150000
1	1.166358000	5.370090000	-1.943770000
1	1.745164000	6.526905000	-0.723952000
6	-0.366098000	6.491401000	0.682193000
1	0.308167000	7.342531000	0.553151000
1	-1.371694000	6.795938000	0.354378000
1	-0.411738000	6.247240000	1.754520000
6	-2.656644000	0.446554000	-0.092007000
8	-3.796047000	0.431222000	0.557271000
7	-2.149853000	-0.593866000	-0.731500000
5	-4.499324000	-0.802183000	0.934292000

6	-2.854102000	-1.748741000	-0.754684000
6	-2.398203000	-2.842260000	-1.547945000
7	-3.997619000	-1.930147000	-0.059635000
7	-3.044781000	-3.983096000	-1.634756000
6	-4.197098000	-4.116724000	-0.934343000
6	-4.675435000	-3.097427000	-0.144220000
9	-5.864036000	-0.660668000	0.770930000
9	-4.155766000	-1.197983000	2.217843000
1	-1.469755000	-2.713111000	-2.106000000
1	-5.591060000	-3.161997000	0.440949000
1	-4.729587000	-5.062526000	-1.013826000
9	2.561412000	-3.514794000	0.719044000
9	6.658928000	-1.223504000	0.461611000
9	5.248032000	-3.479225000	1.029771000
9	2.683376000	0.981764000	-0.737764000
9	5.368599000	1.004320000	-0.422042000
6	2.559435000	-1.267661000	-0.014565000
6	3.232870000	-2.402814000	0.436776000
6	4.677834000	-0.098768000	-0.143594000
6	4.615732000	-2.391601000	0.598843000
6	5.339237000	-1.237837000	0.308268000
6	3.295808000	-0.118327000	-0.303325000
17	0.855793000	-1.270440000	-0.214386000
**Total Energy**		-2208.53621760	

Table S32: Cartesian coordinates and total energy in a.u. of the ground state optimized geometry of **14PN**:C_6F_5Br complex with the halogen bond at the *imine* nitrogen at the MN15/aug-cc-pVDZ-PP level of theory.

Atomic Number	X (Å)	Y (Å)	Z (Å)
6	-2.825450000	2.540080000	0.758029000
6	-2.276819000	1.574337000	-0.104356000
6	-2.370734000	3.847875000	0.746524000
1	-2.813905000	4.564040000	1.434579000
6	-1.265658000	1.970663000	-0.998351000
6	-1.338111000	4.249022000	-0.140937000
1	-0.850449000	1.236095000	-1.686282000
6	-0.800787000	3.273380000	-1.021214000
1	-0.014907000	3.537948000	-1.724819000
1	-3.613715000	2.245200000	1.449051000
7	-0.876220000	5.537423000	-0.150677000
6	0.188593000	5.919250000	-1.057312000
1	1.101801000	5.332841000	-0.875363000
1	-0.107847000	5.781437000	-2.108163000
1	0.422958000	6.976130000	-0.903592000
6	-1.441484000	6.518219000	0.754480000
1	-0.945400000	7.478834000	0.591109000
1	-2.519606000	6.653394000	0.579873000
1	-1.295092000	6.228700000	1.806275000
6	-2.741548000	0.194808000	-0.063063000
8	-3.813569000	-0.033240000	0.655652000
7	-2.096166000	-0.736169000	-0.748703000
5	-4.222321000	-1.379760000	1.081448000

6	-2.589233000	-1.998483000	-0.759425000
6	-2.014774000	-2.979267000	-1.618289000
7	-3.627179000	-2.387134000	0.010735000
7	-2.464116000	-4.212438000	-1.696058000
6	-3.519454000	-4.556106000	-0.920339000
6	-4.102307000	-3.650669000	-0.063455000
9	-5.597165000	-1.502466000	1.070421000
9	-3.663693000	-1.702585000	2.307654000
1	-1.166594000	-2.682466000	-2.236840000
1	-4.945874000	-3.883061000	0.583960000
1	-3.888553000	-5.577330000	-0.993240000
9	2.732683000	-3.129398000	0.405470000
9	6.739944000	-0.691248000	0.651958000
9	5.392106000	-3.046926000	0.853633000
9	2.747690000	1.523336000	-0.453808000
9	5.406794000	1.589510000	-0.003260000
6	2.665236000	-0.805935000	-0.034852000
6	3.366455000	-1.962470000	0.301150000
6	4.746256000	0.437719000	0.096232000
6	4.738767000	-1.932689000	0.534261000
6	5.429502000	-0.728282000	0.431334000
6	3.374628000	0.390282000	-0.133647000
35	0.815412000	-0.837463000	-0.348724000
**Total Energy**		-2165.70961277	

Table S33: Cartesian coordinates and total energy in a.u. of the ground state optimized geometry of **14PN**:C_6F_5I complex with the halogen bond at the *imine* nitrogen at the MN15/aug-cc-pVDZ-PP level of theory.

**Atomic Number**	**X (Å)**	**Y (Å)**	**Z (Å)**
6	-3.574140000	1.871371000	0.836401000
6	-2.816396000	1.100955000	-0.063805000
6	-3.522623000	3.254434000	0.811659000
1	-4.115716000	3.815262000	1.530012000
6	-2.015067000	1.768223000	-1.008229000
6	-2.704675000	3.932734000	-0.129655000
1	-1.443742000	1.182037000	-1.726687000
6	-1.955853000	3.149606000	-1.047417000
1	-1.326252000	3.627530000	-1.793871000
1	-4.203111000	1.364475000	1.566490000
7	-2.642888000	5.299238000	-0.155704000
6	-1.792346000	5.969519000	-1.119879000
1	-0.738066000	5.680070000	-0.996037000
1	-2.095739000	5.741259000	-2.152812000
1	-1.871083000	7.049817000	-0.970853000
6	-3.421048000	6.079409000	0.786381000
1	-3.238442000	7.141679000	0.602840000
1	-4.499132000	5.890477000	0.672260000
1	-3.138330000	5.855448000	1.826155000
6	-2.868807000	-0.352720000	-0.015795000
8	-3.812921000	-0.873637000	0.728543000
7	-2.009627000	-1.068339000	-0.728218000
5	-3.778560000	-2.274373000	1.177105000

6	-2.155182000	-2.418225000	-0.760402000
6	-1.397157000	-3.195833000	-1.681153000
7	-3.012211000	-3.079267000	0.044906000
7	-1.517174000	-4.501978000	-1.780118000
6	-2.400589000	-5.124668000	-0.965785000
6	-3.149238000	-4.420479000	-0.049982000
9	-5.054506000	-2.786912000	1.282571000
9	-3.042870000	-2.401042000	2.343477000
1	-0.693667000	-2.676586000	-2.333377000
1	-3.867268000	-4.877087000	0.628565000
1	-2.496193000	-6.205038000	-1.056178000
53	0.904892000	-0.458255000	-0.342927000
9	3.352056000	-2.462969000	0.276939000
9	6.982151000	0.484425000	0.677659000
9	5.974960000	-2.043262000	0.717842000
9	2.714147000	2.200604000	-0.252432000
9	5.339442000	2.599385000	0.190567000
6	2.947449000	-0.144827000	-0.000359000
6	3.810665000	-1.209947000	0.250743000
6	4.844677000	1.363012000	0.210946000
6	5.169379000	-1.010458000	0.480449000
6	5.685990000	0.282164000	0.459965000
6	3.489715000	1.138977000	-0.015907000
**Total Energy**		-2043.41744675	

Table S34: Cartesian coordinates and total energy in a.u. of the ground state optimized geometry of **15PN**:C_6F_5Cl complex with the halogen bond at the *imine* nitrogen at the MN15/aug-cc-pVDZ level of theory.

Atomic Number	X (Å)	Y (Å)	Z (Å)
6	4.292568000	0.446246000	0.489777000
6	3.075048000	0.183804000	-0.161817000
6	4.880761000	1.698512000	0.427313000
1	5.821970000	1.863441000	0.946373000
6	2.463317000	1.222751000	-0.885479000
6	4.266575000	2.752365000	-0.297567000
1	1.524103000	1.022916000	-1.399744000
6	3.038601000	2.479708000	-0.955689000
1	2.534649000	3.257814000	-1.524090000
1	4.773217000	-0.351902000	1.053126000
7	4.840886000	3.994059000	-0.361174000
6	4.193136000	5.056159000	-1.104541000
1	4.086585000	4.797027000	-2.168930000
1	3.193757000	5.279639000	-0.701252000
1	4.801749000	5.961683000	-1.032141000
6	6.095611000	4.248732000	0.318274000
1	6.386058000	5.289101000	0.148253000
1	6.006755000	4.088695000	1.403551000
1	6.899319000	3.600139000	-0.062089000
6	2.457586000	-1.135079000	-0.082431000
8	3.134294000	-2.047317000	0.565666000
7	1.288763000	-1.338158000	-0.670137000
5	2.565113000	-3.339820000	0.984594000

6	0.739039000	-2.571535000	-0.629651000
6	-0.451678000	-2.859560000	-1.335884000
7	1.294875000	-3.592095000	0.078086000
6	-0.960765000	-4.136054000	-1.264458000
7	-0.375194000	-5.137583000	-0.558864000
6	0.724828000	-4.818222000	0.080352000
1	1.248352000	-5.571980000	0.667864000
9	2.141186000	-3.281440000	2.303495000
9	3.469049000	-4.362311000	0.765983000
1	-0.929572000	-2.069407000	-1.909204000
1	-1.880604000	-4.396304000	-1.790266000
9	-3.561727000	-1.240734000	-0.924585000
9	-6.903978000	1.776877000	0.431476000
9	-6.179085000	-0.615316000	-0.646001000
9	-2.376806000	2.931110000	0.958321000
9	-4.996896000	3.544419000	1.230330000
6	-2.909222000	0.830082000	0.014260000
6	-3.897852000	-0.066326000	-0.392313000
6	-4.640788000	2.375301000	0.706036000
6	-5.246324000	0.247631000	-0.253505000
6	-5.618243000	1.471465000	0.297507000
6	-3.293861000	2.053041000	0.563566000
17	-1.252193000	0.422097000	-0.162870000
**Total Energy**		-2208.54716277	

Table S35: Cartesian coordinates and total energy in a.u. of the ground state optimized geometry of **15PN**:C_6F_5Br complex with the halogen bond at the *imine* nitrogen at the MN15/aug-cc-pVDZ-PP level of theory.

Atomic Number	X (Å)	Y (Å)	Z (Å)
6	2.875426000	2.514943000	0.726266000
6	2.301116000	1.548215000	-0.118916000
6	2.439847000	3.828643000	0.707133000
1	2.902143000	4.545241000	1.381994000
6	1.284562000	1.950344000	-1.004465000
6	1.401337000	4.235809000	-0.171219000
1	0.850989000	1.215122000	-1.680086000
6	0.838789000	3.259409000	-1.035000000
1	0.048419000	3.528844000	-1.731650000
1	3.668189000	2.215335000	1.410030000
7	0.958106000	5.530113000	-0.187817000
6	-0.113930000	5.918321000	-1.083403000
1	0.166144000	5.766753000	-2.136800000
1	-1.032334000	5.346227000	-0.883169000
1	-0.331632000	6.979671000	-0.936376000
6	1.548963000	6.511790000	0.700292000
1	1.064702000	7.477756000	0.533255000
1	1.411718000	6.234899000	1.756643000
1	2.626485000	6.629437000	0.510521000
6	2.747055000	0.163511000	-0.067503000
8	3.818989000	-0.070912000	0.642571000
7	2.075666000	-0.764070000	-0.739444000
5	4.230351000	-1.423801000	1.058937000

6	2.537335000	-2.035074000	-0.746352000
6	1.935550000	-3.020616000	-1.560997000
7	3.594810000	-2.426336000	0.013793000
6	2.464524000	-4.291515000	-1.544976000
7	3.534835000	-4.647007000	-0.789997000
6	4.047924000	-3.698832000	-0.042277000
1	4.905544000	-3.914367000	0.594209000
9	3.710127000	-1.727246000	2.307027000
9	5.604205000	-1.549939000	1.008284000
1	1.081704000	-2.747151000	-2.176644000
1	2.030389000	-5.081143000	-2.160093000
9	-2.803213000	-3.118067000	0.261505000
9	-6.757791000	-0.614803000	0.660841000
9	-5.460848000	-3.006942000	0.714137000
9	-2.717740000	1.578488000	-0.305151000
9	-5.375543000	1.673311000	0.148850000
6	-2.685420000	-0.773158000	-0.030459000
6	-3.411822000	-1.933115000	0.231916000
6	-4.739777000	0.503933000	0.177121000
6	-4.783463000	-1.888926000	0.466166000
6	-5.448191000	-0.665861000	0.438852000
6	-3.369046000	0.441689000	-0.055002000
35	-0.835928000	-0.825414000	-0.345009000
**Total Energy**		-2165.72061441	

Table S36: Cartesian coordinates and total energy in a.u. of the ground state optimized geometry of **15PN**:C_6F_5I complex with the halogen bond at the *imine* nitrogen at the MN15/aug-cc-pVDZ-PP level of theory.

Atomic Number	X (Å)	Y (Å)	Z (Å)
6	3.505369000	1.977144000	0.802580000
6	2.772970000	1.167267000	-0.084605000
6	3.400526000	3.356336000	0.761895000
1	3.974108000	3.948191000	1.471086000
6	1.943682000	1.792032000	-1.034217000
6	2.553126000	3.991489000	-0.184070000
1	1.393573000	1.175622000	-1.743743000
6	1.832241000	3.169447000	-1.090269000
1	1.183183000	3.614157000	-1.840536000
1	4.156145000	1.503273000	1.535741000
7	2.438380000	5.353663000	-0.225100000
6	1.557369000	5.979619000	-1.191935000
1	1.864689000	5.752105000	-2.223806000
1	0.515970000	5.650302000	-1.059243000
1	1.594449000	7.063705000	-1.054965000
6	3.187456000	6.174351000	0.706453000
1	2.962864000	7.226463000	0.511537000
1	2.915886000	5.951023000	1.749274000
1	4.271739000	6.026566000	0.591372000
6	2.880811000	-0.281875000	-0.017037000
8	3.850230000	-0.754544000	0.720439000
7	2.033270000	-1.036305000	-0.709848000
5	3.890271000	-2.159374000	1.168858000

6	2.217395000	-2.378657000	-0.733912000
6	1.467122000	-3.199387000	-1.604418000
7	3.130258000	-2.995741000	0.061977000
6	1.720405000	-4.553342000	-1.601971000
7	2.654355000	-5.138001000	-0.810902000
6	3.314357000	-4.332785000	-0.012116000
1	4.076351000	-4.734100000	0.654971000
9	3.198336000	-2.306672000	2.359679000
9	5.192750000	-2.607073000	1.241690000
1	0.722543000	-2.743061000	-2.252631000
1	1.164035000	-5.222674000	-2.259766000
53	-0.896244000	-0.498540000	-0.335389000
9	-3.352913000	-2.549218000	0.066691000
9	-6.987442000	0.355538000	0.682484000
9	-5.981123000	-2.167082000	0.509089000
9	-2.707580000	2.142397000	-0.035129000
9	-5.338152000	2.503353000	0.407472000
6	-2.943949000	-0.215843000	0.004698000
6	-3.810793000	-1.297769000	0.147857000
6	-4.843857000	1.269535000	0.324482000
6	-5.172421000	-1.118044000	0.376431000
6	-5.688569000	0.171903000	0.465039000
6	-3.486000000	1.064879000	0.096421000
**Total Energy**		-2043.42846797	

Table S37: Cartesian coordinates and total energy in a.u. of the ground state optimized geometry of **16PN**:C_6F_5Cl complex with the halogen bond at the *imine* nitrogen at the MN15/aug-cc-pVDZ level of theory.

Atomic Number	X (Å)	Y (Å)	Z (Å)
6	4.094681000	0.413049000	0.698249000
6	2.987500000	0.118311000	-0.114828000
6	4.714642000	1.650766000	0.640057000
1	5.569772000	1.840659000	1.284472000
6	2.520004000	1.107976000	-0.996362000
6	4.246394000	2.654923000	-0.245367000
1	1.666811000	0.882539000	-1.635113000
6	3.129908000	2.349397000	-1.066260000
1	2.739273000	3.089183000	-1.761066000
1	4.466460000	-0.349757000	1.380503000
7	4.854572000	3.881777000	-0.308129000
6	4.362017000	4.889227000	-1.225293000
1	4.418162000	4.546838000	-2.269996000
1	3.317642000	5.159248000	-1.006116000
1	4.975682000	5.788948000	-1.126812000
6	5.994381000	4.170124000	0.538861000
1	6.330916000	5.192315000	0.344721000
1	5.735092000	4.089825000	1.605533000
1	6.832924000	3.486721000	0.335160000
6	2.338838000	-1.189103000	-0.042028000
8	2.821437000	-2.022547000	0.835607000
7	1.302245000	-1.439564000	-0.825449000
5	2.460555000	-3.460970000	0.903259000

6	0.643194000	-2.613733000	-0.680840000
6	-0.564015000	-2.829196000	-1.402338000
7	1.075896000	-3.597031000	0.139494000
6	-1.232007000	-4.008503000	-1.224791000
6	-0.676235000	-4.962816000	-0.334611000
7	0.444224000	-4.757122000	0.317441000
9	2.299069000	-3.832440000	2.215158000
9	3.397182000	-4.214582000	0.218764000
1	-0.911166000	-2.034939000	-2.059605000
1	-2.165402000	-4.220457000	-1.744993000
1	-1.153764000	-5.924015000	-0.150809000
9	-2.179982000	3.034578000	0.802078000
9	-6.736506000	1.906296000	0.540315000
9	-4.778060000	3.681585000	1.182320000
9	-3.490699000	-1.187444000	-0.875965000
9	-6.084429000	-0.528115000	-0.489309000
6	-2.774739000	0.908229000	-0.042000000
6	-3.122065000	2.152815000	0.483391000
6	-5.126980000	0.339511000	-0.173350000
6	-4.457803000	2.492395000	0.681396000
6	-5.461623000	1.584700000	0.353095000
6	-3.789718000	0.008528000	-0.368034000
17	-1.131332000	0.480187000	-0.286733000
**Total Energy**		-2208.50879396	

Table S38: Cartesian coordinates and total energy in a.u. of the ground state optimized geometry of **16PN**:C_6F_5Br complex with the halogen bond at the *imine* nitrogen at the MN15/aug-cc-pVDZ-PP level of theory.

Atomic Number	X (Å)	Y (Å)	Z (Å)
6	3.268728000	2.051310000	0.756916000
6	2.551719000	1.212567000	-0.114029000
6	3.103296000	3.425734000	0.722408000
1	3.670539000	4.040563000	1.417145000
6	1.671620000	1.801550000	-1.038749000
6	2.205976000	4.024952000	-0.199227000
1	1.127459000	1.162207000	-1.732135000
6	1.497679000	3.173897000	-1.087070000
1	0.808615000	3.591057000	-1.817417000
1	3.958814000	1.604694000	1.471040000
7	2.031988000	5.382894000	-0.234327000
6	1.103583000	5.971049000	-1.179054000
1	1.394309000	5.755101000	-2.218421000
1	0.079446000	5.600054000	-1.021942000
1	1.099629000	7.056050000	-1.043404000
6	2.768577000	6.232680000	0.679926000
1	2.494111000	7.274748000	0.494416000
1	2.534282000	5.995896000	1.728993000
1	3.855206000	6.132231000	0.537334000
6	2.722013000	-0.235278000	-0.051959000
8	3.674562000	-0.669269000	0.719935000
7	1.926595000	-1.014911000	-0.773144000
5	3.915026000	-2.093460000	1.066833000

6	2.113075000	-2.356958000	-0.737280000
6	1.312556000	-3.193627000	-1.564199000
7	3.032882000	-2.944157000	0.058206000
6	1.514821000	-4.544784000	-1.519522000
6	2.516092000	-5.041493000	-0.646072000
7	3.244088000	-4.259170000	0.115455000
9	3.465039000	-2.346438000	2.347579000
9	5.241354000	-2.396173000	0.873408000
1	0.568696000	-2.717421000	-2.200163000
1	0.930476000	-5.231346000	-2.130852000
1	2.729106000	-6.105983000	-0.561929000
9	-2.564198000	2.011068000	-0.024667000
9	-6.815129000	0.155202000	0.713368000
9	-5.196393000	2.329601000	0.469466000
9	-3.149312000	-2.683881000	-0.037965000
9	-5.779797000	-2.349197000	0.456807000
6	-2.781209000	-0.345987000	-0.039628000
6	-3.331825000	0.927791000	0.092952000
6	-4.987279000	-1.286556000	0.340731000
6	-4.688770000	1.105319000	0.347072000
6	-5.518108000	-0.006096000	0.471700000
6	-3.627971000	-1.445747000	0.086577000
35	-0.947792000	-0.565502000	-0.379730000
**Total Energy**		-2165.68225591	

Table S39: Cartesian coordinates and total energy in a.u. of the ground state optimized geometry of **16PN**:C_6F_5I complex with the halogen bond at the *imine* nitrogen at the MN15/aug-cc-pVDZ-PP level of theory.

Atomic Number	X (Å)	Y (Å)	Z (Å)
6	3.565657000	1.871084000	0.795286000
6	2.811426000	1.087720000	-0.096179000
6	3.514731000	3.253785000	0.749736000
1	4.104852000	3.824947000	1.462384000
6	2.015910000	1.740644000	-1.054399000
6	2.701720000	3.918181000	-0.205301000
1	1.448508000	1.144031000	-1.767438000
6	1.957789000	3.121885000	-1.115083000
1	1.332857000	3.588924000	-1.872324000
1	4.191262000	1.374068000	1.535056000
7	2.640489000	5.284768000	-0.251597000
6	1.793902000	5.940734000	-1.228465000
1	2.100927000	5.697235000	-2.256935000
1	0.738871000	5.653511000	-1.104538000
1	1.872376000	7.023164000	-1.095351000
6	3.412187000	6.078107000	0.684619000
1	3.230797000	7.137713000	0.484942000
1	3.122723000	5.868797000	1.725646000
1	4.491052000	5.887871000	0.580490000
6	2.865223000	-0.367050000	-0.022065000
8	3.801394000	-0.872503000	0.726874000
7	1.998625000	-1.090973000	-0.722228000
5	3.836091000	-2.292128000	1.164188000

6	2.120352000	-2.443915000	-0.720239000
6	1.310364000	-3.219274000	-1.594459000
7	2.988572000	-3.092193000	0.084920000
6	1.453244000	-4.579433000	-1.582864000
6	2.404620000	-5.143373000	-0.695847000
7	3.141867000	-4.415359000	0.110438000
9	3.194431000	-2.423591000	2.381807000
9	5.130543000	-2.741709000	1.158780000
1	0.609586000	-2.694630000	-2.241256000
1	0.859380000	-5.222132000	-2.231503000
1	2.568915000	-6.218117000	-0.636450000
53	-0.898907000	-0.445541000	-0.332979000
9	-2.645657000	2.221463000	0.103174000
9	-6.975600000	0.508292000	0.688568000
9	-5.270156000	2.624777000	0.547363000
9	-3.411086000	-2.450103000	-0.065822000
9	-6.032063000	-2.025869000	0.379209000
6	-2.942041000	-0.127970000	0.008459000
6	-3.452233000	1.159080000	0.168609000
6	-5.195954000	-0.991951000	0.312498000
6	-4.806478000	1.385457000	0.398259000
6	-5.679958000	0.303806000	0.470495000
6	-3.837499000	-1.193194000	0.083738000
**Total Energy**		-2043.38995373	

Table S40: Cartesian coordinates and total energy in a.u. of the excited state optimized geometry of **PN** dye at the MN15/aug-cc-pVDZ level of theory.

Atomic Number	X (Å)	Y (Å)	Z (Å)
6	1.778414000	-1.173224000	-0.197059000
6	0.998765000	0.002658000	-0.059178000
6	3.157255000	-1.112930000	-0.182462000
1	3.728013000	-2.032482000	-0.289966000
6	1.664820000	1.247548000	0.094458000
6	3.829795000	0.135992000	-0.027758000
1	1.055365000	2.143101000	0.200404000
6	3.040380000	1.317676000	0.109938000
1	3.520152000	2.286213000	0.230333000
1	1.262983000	-2.124785000	-0.311578000
7	5.194531000	0.198744000	-0.011852000
6	5.868261000	1.474585000	0.144874000
1	5.604852000	2.163493000	-0.671075000
1	5.600513000	1.948858000	1.100534000
1	6.948150000	1.310385000	0.127540000
6	5.987470000	-1.009626000	-0.147250000
1	7.046693000	-0.745248000	-0.107226000
1	5.771470000	-1.717653000	0.666167000
1	5.786494000	-1.509226000	-1.106250000
6	-0.460055000	-0.071483000	-0.076892000
8	-0.970143000	-1.282719000	-0.249359000
7	-1.134752000	1.055904000	0.048859000
5	-2.376439000	-1.520211000	0.135279000
6	-2.495782000	1.030010000	-0.003655000

6	-3.228146000	2.222863000	0.005247000
7	-3.158918000	-0.190022000	-0.097279000
6	-4.616872000	2.208082000	-0.095585000
6	-5.266610000	0.947981000	-0.206213000
6	-4.514067000	-0.206805000	-0.194688000
9	-2.442106000	-1.854029000	1.492478000
9	-2.923532000	-2.518584000	-0.660888000
1	-4.959320000	-1.198392000	-0.263251000
1	-6.349309000	0.874625000	-0.292898000
1	-5.187523000	3.134909000	-0.090699000
1	-2.662246000	3.150187000	0.081368000
**Total Energy**		-1005.16776517	

Table S41: Cartesian coordinates and total energy in a.u. of the excited state optimized geometry of **13PN** dye at the MN15/aug-cc-pVDZ level of theory.

Atomic Number	X (Å)	Y (Å)	Z (Å)
6	-1.774716000	1.184427000	-0.000234000
6	-1.013122000	-0.002626000	-0.000092000
6	-3.152113000	1.132155000	-0.000228000
1	-3.722066000	2.057624000	-0.000236000
6	-1.667065000	-1.255517000	0.000083000
6	-3.824528000	-0.130647000	-0.000034000
1	-1.044527000	-2.148405000	0.000139000
6	-3.040757000	-1.327924000	0.000130000
1	-3.524870000	-2.300958000	0.000160000
1	-1.247800000	2.136105000	-0.000305000
7	-5.179033000	-0.190531000	-0.000011000

6	-5.864571000	-1.475834000	0.000930000
1	-5.600374000	-2.056765000	-0.892235000
1	-5.598620000	-2.056297000	0.893853000
1	-6.941977000	-1.301850000	0.001968000
6	-5.977812000	1.027878000	-0.000965000
1	-7.035041000	0.757045000	-0.001943000
1	-5.767818000	1.630291000	0.892343000
1	-5.766104000	1.629920000	-0.894092000
6	0.481685000	0.059775000	-0.000106000
8	0.949840000	1.280093000	-0.000324000
7	1.098104000	-1.067613000	0.000035000
5	2.428466000	1.514593000	0.000299000
6	2.514446000	-1.072380000	-0.000112000
7	3.096432000	-2.228149000	-0.000224000
7	3.134316000	0.147782000	-0.000091000
6	4.489442000	-2.242733000	-0.000331000
6	5.209461000	-1.047134000	-0.000325000
6	4.535637000	0.155433000	-0.000208000
9	2.759946000	2.239312000	1.146058000
9	2.760743000	2.240321000	-1.144564000
1	5.007775000	1.132393000	-0.000203000
1	6.299160000	-1.052596000	-0.000418000
1	4.970351000	-3.216573000	-0.000394000
**Total Energy**		-1021.18253129	

Table S42: Cartesian coordinates and total energy in a.u. of the excited state optimized geometry of **14PN**dye at the MN15/aug-cc-pVDZ level of theory.

Atomic Number	X (Å)	Y (Å)	Z (Å)
6	-1.768366000	1.164432000	-0.170679000
6	-1.007468000	-0.015279000	-0.037342000
6	-3.147982000	1.115945000	-0.165791000
1	-3.713426000	2.038431000	-0.271031000
6	-1.668532000	-1.255200000	0.104779000
6	-3.824873000	-0.133019000	-0.022870000
1	-1.056643000	-2.149375000	0.206624000
6	-3.044210000	-1.322626000	0.112714000
1	-3.530110000	-2.288627000	0.224133000
1	-1.241520000	2.110615000	-0.274729000
7	-5.183470000	-0.189502000	-0.015392000
6	-5.869865000	-1.464127000	0.128452000
1	-5.603502000	-2.145234000	-0.691080000
1	-5.610472000	-1.941868000	1.082997000
1	-6.947434000	-1.290682000	0.104232000
6	-5.975274000	1.023888000	-0.148658000
1	-7.034318000	0.760877000	-0.115702000
1	-5.759365000	1.722636000	0.670868000
1	-5.765262000	1.523976000	-1.103852000
6	0.482806000	0.048537000	-0.044304000
8	0.957395000	1.268837000	-0.165577000
7	1.124441000	-1.065896000	0.054442000
5	2.400579000	1.525420000	0.115169000
6	2.521340000	-1.035735000	-0.002666000

6	3.246448000	-2.203414000	-0.020759000
7	3.151773000	0.186626000	-0.074018000
7	4.607953000	-2.257851000	-0.101363000
6	5.200822000	-1.042914000	-0.170266000
6	4.524118000	0.159709000	-0.157268000
9	2.526700000	1.971104000	1.433264000
9	2.880813000	2.470556000	-0.782352000
1	2.697945000	-3.143581000	0.025965000
1	5.025632000	1.122863000	-0.209977000
1	6.290125000	-1.027588000	-0.235611000
**Total Energy**		-1021.18258492	

Table S43: Cartesian coordinates and total energy in a.u. of the excited state optimized geometry of **15PN** dye at the MN15/aug-cc-pVDZ level of theory.

Atomic Number	X (Å)	Y (Å)	Z (Å)
6	1.767201000	-1.159204000	-0.225639000
6	0.999859000	0.018456000	-0.057433000
6	3.148398000	-1.108799000	-0.213705000
1	3.712608000	-2.029189000	-0.345551000
6	1.670484000	1.254237000	0.125740000
6	3.828010000	0.131312000	-0.030767000
1	1.065830000	2.149938000	0.256141000
6	3.047457000	1.315570000	0.138346000
1	3.534273000	2.277468000	0.281446000
1	1.243885000	-2.103532000	-0.361284000
7	5.194575000	0.186052000	-0.016540000
6	5.877692000	1.453216000	0.167032000

1	5.613040000	2.162309000	-0.630962000
1	5.616572000	1.905683000	1.135026000
1	6.956170000	1.282644000	0.140014000
6	5.979945000	-1.023479000	-0.180057000
1	7.040741000	-0.767341000	-0.134759000
1	5.757635000	-1.747932000	0.617061000
1	5.773136000	-1.499194000	-1.149962000
6	-0.472276000	-0.045207000	-0.073568000
8	-0.977662000	-1.258260000	-0.252009000
7	-1.141773000	1.076784000	0.055921000
5	-2.378185000	-1.504780000	0.153774000
6	-2.502015000	1.044019000	-0.003176000
6	-3.277557000	2.210151000	-0.017968000
7	-3.167496000	-0.173817000	-0.085246000
6	-4.652553000	2.099455000	-0.135005000
7	-5.284138000	0.885078000	-0.238392000
6	-4.516892000	-0.176705000	-0.199168000
1	-4.970645000	-1.165822000	-0.264761000
9	-2.422266000	-1.813015000	1.515656000
9	-2.928511000	-2.512269000	-0.623533000
1	-2.776877000	3.174165000	0.049667000
1	-5.291857000	2.981527000	-0.151978000
**Total Energy**		-1021.2027073	

Table S44: Cartesian coordinates and total energy in a.u. of the excited state optimized geometry of **16PN** dye at the MN15/aug-cc-pVDZ level of theory.

**Atomic Number**	**X (Å)**	**Y (Å)**	**Z (Å)**

6	1.769540000	-1.164194000	-0.000186000
6	1.016636000	0.028116000	0.000006000
6	3.147972000	-1.123101000	-0.000155000
1	3.709608000	-2.053661000	-0.000285000
6	1.681050000	1.273979000	0.000264000
6	3.830487000	0.132952000	0.000072000
1	1.069640000	2.173997000	0.000438000
6	3.056281000	1.335964000	0.000299000
1	3.548141000	2.305205000	0.000527000
1	1.234341000	-2.111278000	-0.000347000
7	5.186794000	0.182446000	0.000080000
6	5.882315000	1.461670000	0.000454000
1	5.621979000	2.045228000	-0.892333000
1	5.622354000	2.044533000	0.893815000
1	6.958374000	1.279277000	0.000139000
6	5.974843000	-1.042397000	0.000039000
1	7.034475000	-0.780916000	-0.000146000
1	5.758661000	-1.643132000	0.893182000
1	5.758399000	-1.643214000	-0.892978000
6	-0.481189000	-0.033516000	-0.000027000
8	-0.943684000	-1.250728000	-0.000258000
7	-1.109797000	1.088525000	0.000046000
5	-2.431596000	-1.521038000	0.000110000
6	-2.520515000	1.063857000	-0.000148000
6	-3.221587000	2.240647000	-0.000284000
7	-3.143685000	-0.147185000	-0.000221000

6	-4.651494000	2.161752000	-0.000476000
6	-5.198635000	0.879489000	-0.000475000
7	-4.514686000	-0.259677000	-0.000346000
9	-2.722863000	-2.238652000	1.151176000
9	-2.723279000	-2.239454000	-1.150322000
1	-2.670378000	3.176504000	-0.000234000
1	-5.286527000	3.043237000	-0.000589000
1	-6.280943000	0.738134000	-0.000587000
**Total Energy**		-1021.15037560	

Table S45: Cartesian coordinates and total energy in a.u. of the excited state optimized geometry of **13PN**:C_6F_5Cl complex with the halogen bond at the *heterocyclic* nitrogen at the MN15/aug-cc-pVDZ level of theory.

Atomic Number	X (Å)	Y (Å)	Z (Å)
6	3.154321000	2.613696000	-0.019436000
6	2.283388000	1.504748000	-0.010943000
6	2.650333000	3.896944000	-0.018445000
1	3.335970000	4.740365000	-0.024747000
6	0.885054000	1.707930000	-0.001816000
6	1.235925000	4.114197000	-0.008757000
1	0.242792000	0.828950000	0.004107000
6	0.363193000	2.980164000	-0.000718000
1	-0.715246000	3.114382000	0.006342000
1	4.226572000	2.430536000	-0.026064000
7	0.731452000	5.372354000	-0.007090000
6	-0.708881000	5.593705000	0.002727000
1	-1.174975000	5.148894000	-0.885811000

1	-1.162343000	5.151971000	0.899308000
1	-0.901056000	6.667944000	0.002241000
6	1.616473000	6.529415000	-0.014543000
1	1.011113000	7.437387000	-0.011300000
1	2.260867000	6.530334000	0.874139000
1	2.248412000	6.528419000	-0.912126000
6	2.831256000	0.111600000	-0.010749000
8	4.136232000	0.062443000	-0.023109000
7	1.967238000	-0.838993000	-0.000119000
5	4.837864000	-1.260450000	0.026495000
6	2.423281000	-2.178008000	-0.008250000
7	1.513201000	-3.100050000	-0.016111000
7	3.774121000	-2.373026000	-0.011073000
6	1.946750000	-4.425822000	-0.026081000
6	3.308915000	-4.722357000	-0.027789000
6	4.233072000	-3.698311000	-0.020088000
9	5.574783000	-1.324397000	1.209689000
9	5.682294000	-1.354548000	-1.078793000
1	5.309744000	-3.831121000	-0.021715000
1	3.652701000	-5.756237000	-0.034907000
1	1.179105000	-5.193793000	-0.031436000
9	-2.297790000	1.125199000	0.007015000
9	-6.874201000	0.057087000	0.010041000
9	-4.870872000	1.910377000	0.013535000
9	-3.688217000	-3.398269000	-0.006727000
9	-6.263745000	-2.596140000	-0.000126000
6	-2.917156000	-1.163828000	0.000054000

6	-3.255455000	0.189031000	0.005286000
6	-5.288726000	-1.691856000	0.001650000
6	-4.580981000	0.608042000	0.008655000
6	-5.603631000	-0.335572000	0.006852000
6	-3.956273000	-2.097525000	-0.001719000
17	-1.281196000	-1.671074000	-0.004230000
**Total Energy**		-2208.52446584	

Table S46: Cartesian coordinates and total energy in a.u. of the excited state optimized geometry of **13PN**:C_6F_5Br complex with the halogen bond at the *heterocyclic* nitrogen at the MN15/aug-cc-pVDZ-PP level of theory.

Atomic Number	X (Å)	Y (Å)	Z (Å)
6	3.419291000	2.577388000	-0.009617000
6	2.477659000	1.529128000	-0.005627000
6	2.997930000	3.890349000	-0.010211000
1	3.735623000	4.688640000	-0.013234000
6	1.094677000	1.819606000	-0.002210000
6	1.599562000	4.197519000	-0.006659000
1	0.397949000	0.982704000	0.000465000
6	0.655113000	3.122116000	-0.002649000
1	-0.413272000	3.322710000	-0.000066000
1	4.477772000	2.325991000	-0.011869000
7	1.177007000	5.485066000	-0.007090000
6	-0.246619000	5.798176000	-0.003452000
1	-0.735903000	5.383793000	-0.894105000
1	-0.730876000	5.385856000	0.890896000
1	-0.369627000	6.882455000	-0.004352000

6	2.133921000	6.583582000	-0.011146000
1	1.587518000	7.528142000	-0.010838000
1	2.773043000	6.543864000	0.880387000
1	2.768085000	6.541583000	-0.906110000
6	2.924910000	0.099984000	-0.004946000
8	4.219683000	-0.056040000	-0.010366000
7	1.988351000	-0.777981000	-0.000505000
5	4.809235000	-1.435677000	0.023348000
6	2.331054000	-2.145827000	-0.007390000
7	1.340727000	-2.984871000	-0.015406000
7	3.654125000	-2.458369000	-0.008066000
6	1.654093000	-4.346607000	-0.023843000
6	2.983766000	-4.758571000	-0.024309000
6	3.996505000	-3.819926000	-0.016387000
9	5.547489000	-1.570595000	1.197597000
9	5.627355000	-1.593291000	-1.092723000
1	5.057087000	-4.046645000	-0.016683000
1	3.234665000	-5.818697000	-0.030682000
1	0.821013000	-5.042638000	-0.029377000
9	-2.062531000	1.267795000	0.001273000
9	-6.721791000	0.703603000	0.012856000
9	-4.524234000	2.323693000	0.010059000
9	-3.943516000	-3.081354000	-0.002265000
9	-6.406115000	-2.003068000	0.006636000
6	-2.913268000	-0.951082000	-0.000786000
6	-3.110926000	0.428206000	0.002506000
6	-5.337125000	-1.209992000	0.005305000

6	-4.380180000	0.995631000	0.007077000
6	-5.501317000	0.172173000	0.008503000
6	-4.054870000	-1.753985000	0.000703000
35	-1.186376000	-1.705321000	-0.006881000
**Total Energy**		-2165.69774393	

Table S47: Cartesian coordinates and total energy in a.u. of the excited state optimized geometry of **13PN**:C_6F_5I complex with the halogen bond at the *heterocyclic* nitrogen at the MN15/aug-cc-pVDZ-PP level of theory.

Atomic Number	X (Å)	Y (Å)	Z (Å)
6	3.540427000	2.679425000	-0.002676000
6	2.601852000	1.629035000	-0.001211000
6	3.114799000	3.991178000	-0.002888000
1	3.849829000	4.791886000	-0.004014000
6	1.217558000	1.914272000	0.000021000
6	1.715112000	4.293581000	-0.001605000
1	0.519271000	1.078152000	0.000982000
6	0.773775000	3.215346000	-0.000174000
1	-0.295626000	3.410908000	0.000697000
1	4.599878000	2.431913000	-0.003554000
7	1.288105000	5.579278000	-0.001783000
6	-0.137009000	5.887548000	-0.000544000
1	-0.622845000	5.471719000	-0.892279000
1	-0.621101000	5.472604000	0.892553000
1	-0.263609000	6.971342000	-0.000962000
6	2.240861000	6.681557000	-0.003208000
1	1.690876000	7.623952000	-0.003006000

1	2.878401000	6.643087000	0.889459000
1	2.876549000	6.642382000	-0.897170000
6	3.052226000	0.199459000	-0.000996000
8	4.344786000	0.038274000	-0.002906000
7	2.115267000	-0.677576000	0.000624000
5	4.930886000	-1.345126000	0.005107000
6	2.454022000	-2.042828000	-0.001143000
7	1.457334000	-2.879172000	-0.002730000
7	3.769648000	-2.365752000	-0.001706000
6	1.758965000	-4.245594000	-0.004810000
6	3.085022000	-4.663197000	-0.005262000
6	4.104750000	-3.730578000	-0.003712000
9	5.696542000	-1.492638000	1.157888000
9	5.714589000	-1.498484000	-1.134473000
1	5.163808000	-3.963354000	-0.004069000
1	3.328758000	-5.724855000	-0.006782000
1	0.919349000	-4.933655000	-0.005881000
53	-1.060407000	-1.722553000	-0.001202000
9	-2.032307000	1.375046000	0.001309000
9	-6.710169000	1.014721000	0.002615000
9	-4.444191000	2.536899000	0.002889000
9	-4.108401000	-2.891637000	-0.000974000
9	-6.517686000	-1.702553000	0.000678000
6	-2.975398000	-0.810841000	0.000088000
6	-3.116641000	0.574365000	0.001109000
6	-5.411884000	-0.959264000	0.000816000
6	-4.357213000	1.202368000	0.001952000

6	-5.513569000	0.428912000	0.001810000
6	-4.153080000	-1.556573000	-0.000026000
**Total Energy**		-2043.40519892	

Table S48: Cartesian coordinates and total energy in a.u. of the excited state optimized geometry of **14PN**:C_6F_5Cl complex with the halogen bond at the *heterocyclic* nitrogen at the MN15/aug-cc-pVDZ level of theory.

Atomic Number	X (Å)	Y (Å)	Z (Å)
6	5.602467000	0.127576000	-0.148870000
6	4.202009000	0.250625000	-0.041842000
6	6.408605000	1.248171000	-0.148643000
1	7.485889000	1.129211000	-0.232874000
6	3.623180000	1.533896000	0.067935000
6	5.832686000	2.550046000	-0.037675000
1	2.539887000	1.598857000	0.149491000
6	4.411773000	2.663124000	0.070712000
1	3.943456000	3.640325000	0.156770000
1	6.029732000	-0.869794000	-0.228873000
7	6.619207000	3.658842000	-0.034523000
6	6.029307000	4.984200000	0.077815000
1	5.343759000	5.177872000	-0.758175000
1	5.474556000	5.085667000	1.020379000
1	6.826757000	5.729436000	0.057753000
6	8.065661000	3.540604000	-0.140839000
1	8.505238000	4.539570000	-0.116927000
1	8.471533000	2.955989000	0.695760000
1	8.348751000	3.052638000	-1.083297000

6	3.339408000	-0.967751000	-0.041607000
8	4.015001000	-2.090753000	-0.141110000
7	2.065471000	-0.791205000	0.042717000
5	3.333307000	-3.388158000	0.140165000
6	1.234538000	-1.915509000	-0.004336000
6	-0.131775000	-1.773892000	-0.031017000
7	1.814557000	-3.162700000	-0.053491000
7	-1.006259000	-2.821082000	-0.099811000
6	-0.410147000	-4.035205000	-0.147523000
6	0.954571000	-4.233805000	-0.125265000
9	3.603454000	-3.758778000	1.459196000
9	3.787672000	-4.348384000	-0.754435000
1	-0.542734000	-0.764478000	-0.000918000
1	1.410422000	-5.219937000	-0.161241000
1	-1.064303000	-4.906599000	-0.202992000
9	-3.078481000	1.747284000	0.032559000
9	-7.607002000	3.009421000	0.062176000
9	-4.967178000	3.676829000	0.090760000
9	-6.467702000	-1.549201000	-0.084216000
9	-8.346249000	0.390857000	-0.025352000
6	-4.723489000	0.047062000	-0.027266000
6	-4.362833000	1.394622000	0.017723000
6	-7.055469000	0.716566000	-0.011361000
6	-5.329449000	2.395348000	0.047896000
6	-6.679336000	2.056033000	0.033377000
6	-6.080988000	-0.277232000	-0.041410000
17	-3.524426000	-1.176945000	-0.063768000

Total Energy	-2208.52110627

Table S49: Cartesian coordinates and total energy in a.u. of the excited state optimized geometry of **14PN**:C_6F_5Br complex with the halogen bond at the *heterocyclic* nitrogen at the MN15/aug-cc-pVDZ-PP level of theory.

Atomic Number	X (Å)	Y (Å)	Z (Å)
6	6.157378000	-0.120144000	-0.169124000
6	4.792328000	0.203547000	-0.035960000
6	7.117275000	0.872044000	-0.172941000
1	8.164433000	0.599806000	-0.277941000
6	4.406046000	1.554369000	0.096499000
6	6.737080000	2.242026000	-0.039245000
1	3.344991000	1.773832000	0.198052000
6	5.349373000	2.558139000	0.096015000
1	5.028706000	3.591549000	0.200053000
1	6.434427000	-1.167749000	-0.266043000
7	7.675073000	3.225356000	-0.039841000
6	7.285024000	4.621055000	0.094663000
1	6.620234000	4.920121000	-0.726708000
1	6.767445000	4.790603000	1.048381000
1	8.181286000	5.243432000	0.065698000
6	9.087825000	2.901472000	-0.172785000
1	9.667041000	3.826281000	-0.145825000
1	9.417655000	2.253655000	0.650407000
1	9.282085000	2.390267000	-1.125241000
6	3.759661000	-0.879341000	-0.032021000
8	4.267234000	-2.084693000	-0.145989000

7	2.527752000	-0.521994000	0.068791000
5	3.410967000	-3.277706000	0.122842000
6	1.542279000	-1.517438000	0.023670000
6	0.212292000	-1.180823000	0.013114000
7	1.936576000	-2.832279000	-0.037545000
7	-0.800641000	-2.099356000	-0.050393000
6	-0.388878000	-3.385777000	-0.108905000
6	0.933797000	-3.773588000	-0.103191000
9	3.645039000	-3.711757000	1.428088000
9	3.707538000	-4.271834000	-0.799519000
1	-0.057786000	-0.125855000	0.052689000
1	1.246673000	-4.813279000	-0.148561000
1	-1.162242000	-4.153184000	-0.160039000
9	-3.483762000	2.303098000	0.065726000
9	-8.166892000	2.644408000	0.026311000
9	-5.708903000	3.817657000	0.092187000
9	-6.157749000	-1.598404000	-0.094066000
9	-8.374526000	-0.070221000	-0.066771000
6	-4.746832000	0.300867000	-0.014993000
6	-4.671103000	1.692181000	0.032517000
6	-7.172680000	0.504479000	-0.034462000
6	-5.812109000	2.489105000	0.046735000
6	-7.068342000	1.891431000	0.013119000
6	-6.017437000	-0.272374000	-0.047965000
35	-3.190387000	-0.762554000	-0.033553000
**Total Energy**		-2165.69250844	

Table S50: Cartesian coordinates and total energy in a.u. of the excited state optimized geometry of **14PN**:C_6F_5I complex with the halogen bond at the *heterocyclic* nitrogen at the MN15/aug-cc-pVDZ-PP level of theory.

Atomic Number	X (Å)	Y (Å)	Z (Å)
6	6.632534000	-0.277224000	-0.192664000
6	5.311590000	0.185274000	-0.033012000
6	7.690416000	0.609869000	-0.202103000
1	8.701723000	0.231753000	-0.327653000
6	5.069125000	1.566112000	0.121437000
6	7.456494000	2.010070000	-0.046892000
1	4.038457000	1.893237000	0.243170000
6	6.111458000	2.466897000	0.115851000
1	5.901662000	3.526471000	0.236842000
1	6.798018000	-1.346493000	-0.305365000
7	8.491148000	2.890606000	-0.053101000
6	8.251028000	4.317721000	0.104176000
1	7.608293000	4.694831000	-0.702388000
1	7.769125000	4.527070000	1.068501000
1	9.206745000	4.843550000	0.067132000
6	9.860516000	2.423970000	-0.215304000
1	10.532650000	3.283568000	-0.190437000
1	10.135321000	1.736260000	0.595525000
1	9.983426000	1.906377000	-1.176021000
6	4.170007000	-0.786387000	-0.024306000
8	4.550853000	-2.034624000	-0.153147000
7	2.983866000	-0.305769000	0.093445000
5	3.581075000	-3.138792000	0.112096000

6	1.900409000	-1.196490000	0.049298000
6	0.613547000	-0.723834000	0.053172000
7	2.157045000	-2.542131000	-0.024256000
7	-0.484202000	-1.543046000	-0.007243000
6	-0.211206000	-2.864409000	-0.077570000
6	1.065368000	-3.381151000	-0.086508000
9	3.782575000	-3.610199000	1.408429000
9	3.762811000	-4.143873000	-0.826466000
1	0.447332000	0.350926000	0.102463000
1	1.274095000	-4.445901000	-0.141725000
1	-1.062962000	-3.544411000	-0.126128000
53	-2.949198000	-0.461230000	-0.007272000
9	-3.981956000	2.617800000	0.092059000
9	-8.651470000	2.155743000	-0.015359000
9	-6.430522000	3.729297000	0.087967000
9	-5.956096000	-1.685474000	-0.112300000
9	-8.395930000	-0.554166000	-0.115210000
6	-4.878238000	0.424083000	-0.009807000
6	-5.045848000	1.806101000	0.040434000
6	-7.307388000	0.215231000	-0.064530000
6	-6.304245000	2.401658000	0.039249000
6	-7.440165000	1.599563000	-0.013553000
6	-6.035120000	-0.350527000	-0.062004000
**Total Energy**		-2043.39927072	

Table S51: Cartesian coordinates and total energy in a.u. of the excited state optimized geometry of **15PN**:C_6F_5Cl complex with the halogen bond at the *heterocyclic* nitrogen at the MN15/aug-cc-pVDZ level of theory.

Atomic Number	X (Å)	Y (Å)	Z (Å)
6	-6.353092000	0.849053000	-0.283002000
6	-5.281313000	-0.046751000	-0.054317000
6	-7.657183000	0.392823000	-0.322898000
1	-8.461098000	1.103330000	-0.500854000
6	-5.565064000	-1.422580000	0.136094000
6	-7.948456000	-0.990081000	-0.133369000
1	-4.729768000	-2.097625000	0.313420000
6	-6.861869000	-1.888194000	0.097483000
1	-7.049439000	-2.948848000	0.247463000
1	-6.125900000	1.903860000	-0.423614000
7	-9.237061000	-1.446196000	-0.170764000
6	-9.524019000	-2.856095000	0.019832000
1	-9.026433000	-3.466260000	-0.748066000
1	-9.185139000	-3.197417000	1.009022000
1	-10.602479000	-3.012204000	-0.053713000
6	-10.336078000	-0.525393000	-0.396922000
1	-11.275011000	-1.083224000	-0.386670000
1	-10.373724000	0.243089000	0.388960000
1	-10.234865000	-0.023107000	-1.370152000
6	-3.893533000	0.448824000	-0.016465000
8	-3.760741000	1.754030000	-0.208848000
7	-2.929792000	-0.423058000	0.168066000
5	-2.514691000	2.410656000	0.240689000

6	-1.638359000	0.008402000	0.159375000
6	-0.553735000	-0.876681000	0.205633000
7	-1.358049000	1.367851000	0.069688000
6	0.731198000	-0.367112000	0.138571000
7	0.978712000	0.979402000	0.027545000
6	-0.067578000	1.768701000	0.008445000
1	0.077582000	2.846476000	-0.065890000
9	-2.621704000	2.740983000	1.593368000
9	-2.250636000	3.521521000	-0.545241000
1	-0.750184000	-1.944365000	0.280431000
1	1.604255000	-1.019465000	0.168727000
9	4.750336000	-2.168699000	0.149987000
9	9.410019000	-1.577605000	-0.064506000
9	7.243802000	-3.212954000	0.145234000
9	6.580455000	2.171334000	-0.266470000
9	9.067867000	1.114275000	-0.269921000
6	5.603334000	0.027982000	-0.057790000
6	5.795784000	-1.350261000	0.046705000
6	8.007920000	0.315987000	-0.167302000
6	7.075929000	-1.896122000	0.044969000
6	8.184701000	-1.060936000	-0.062284000
6	6.723510000	0.853098000	-0.164727000
17	4.020064000	0.685681000	-0.054337000
**Total Energy**		-2208.54209530	

Table S52: Cartesian coordinates and total energy in a.u. of the excited state optimized geometry of **15PN**:C_6F_5Br complex with the halogen bond at the *heterocyclic* nitrogen at the MN15/aug-cc-pVDZ-PP level of theory.

Atomic Number	X (Å)	Y (Å)	Z (Å)
6	-6.556054000	1.003761000	-0.305544000
6	-5.629267000	-0.033846000	-0.054164000
6	-7.911254000	0.738033000	-0.370696000
1	-8.602161000	1.554759000	-0.566064000
6	-6.107055000	-1.353202000	0.133522000
6	-8.399101000	-0.588460000	-0.184022000
1	-5.379274000	-2.138795000	0.328491000
6	-7.456045000	-1.630938000	0.070173000
1	-7.795117000	-2.653461000	0.218459000
1	-6.178115000	2.014787000	-0.443233000
7	-9.737577000	-0.857288000	-0.245563000
6	-10.226089000	-2.211556000	-0.057842000
1	-9.805856000	-2.889176000	-0.815056000
1	-9.957761000	-2.592312000	0.938347000
1	-11.314104000	-2.212821000	-0.151932000
6	-10.690824000	0.209080000	-0.495367000
1	-11.699341000	-0.209862000	-0.501250000
1	-10.632799000	0.978990000	0.287595000
1	-10.500930000	0.685919000	-1.467937000
6	-4.180660000	0.261199000	0.011112000
8	-3.868920000	1.535686000	-0.169704000
7	-3.356005000	-0.734922000	0.207610000
5	-2.549268000	2.012872000	0.301153000

6	-2.012888000	-0.489586000	0.220636000
6	-1.066375000	-1.516417000	0.277386000
7	-1.547253000	0.820251000	0.139865000
6	0.279619000	-1.193568000	0.230564000
7	0.708171000	0.108960000	0.128965000
6	-0.213732000	1.039279000	0.097930000
1	0.083687000	2.085551000	0.029803000
9	-2.632286000	2.349142000	1.653195000
9	-2.122929000	3.078206000	-0.475217000
1	-1.411990000	-2.545992000	0.344028000
1	1.056797000	-1.956401000	0.269771000
9	5.217439000	-2.480648000	0.090845000
9	9.493437000	-0.561057000	-0.198165000
9	7.895002000	-2.757962000	-0.007420000
9	5.707437000	2.217211000	-0.193746000
9	8.383974000	1.923949000	-0.290592000
6	5.377717000	-0.122086000	-0.048131000
6	5.970800000	-1.382966000	-0.002487000
6	7.602283000	0.849548000	-0.197090000
6	7.352682000	-1.541816000	-0.052302000
6	8.170777000	-0.420036000	-0.149925000
6	6.217776000	0.986317000	-0.146061000
35	3.507166000	0.075256000	0.019670000
**Total Energy**		-2165.71418218	

Table S53: Cartesian coordinates and total energy in a.u. of the excited state optimized geometry of **15PN**:C_6F_5I complex with the halogen bond at the *heterocyclic* nitrogen at the MN15/aug-cc-pVDZ-PP level of theory.

Atomic Number	X (Å)	Y (Å)	Z (Å)
6	-6.747492000	1.106531000	-0.271622000
6	-5.909419000	-0.009774000	-0.056761000
6	-8.118946000	0.951725000	-0.358468000
1	-8.741412000	1.827595000	-0.525175000
6	-6.489001000	-1.293018000	0.072921000
6	-8.710517000	-0.338750000	-0.230458000
1	-5.826687000	-2.140376000	0.240662000
6	-7.855060000	-1.461746000	-0.012238000
1	-8.274506000	-2.459629000	0.090874000
1	-6.289885000	2.089352000	-0.364198000
7	-10.064508000	-0.498961000	-0.313211000
6	-10.660137000	-1.817197000	-0.184722000
1	-10.286273000	-2.494431000	-0.965972000
1	-10.433251000	-2.257126000	0.797109000
1	-11.743709000	-1.728631000	-0.287035000
6	-10.929193000	0.648209000	-0.526872000
1	-11.967066000	0.310099000	-0.557449000
1	-10.819465000	1.378918000	0.287266000
1	-10.691810000	1.146566000	-1.477801000
6	-4.438135000	0.167620000	0.031938000
8	-4.031610000	1.420858000	-0.087764000
7	-3.698154000	-0.894808000	0.189947000
5	-2.682542000	1.774542000	0.412635000

6	-2.336709000	-0.756624000	0.220129000
6	-1.476100000	-1.854087000	0.235246000
7	-1.773254000	0.518112000	0.196582000
6	-0.106804000	-1.639143000	0.205932000
7	0.415645000	-0.366874000	0.159483000
6	-0.427691000	0.635800000	0.165581000
1	-0.044919000	1.656341000	0.140913000
9	-2.754193000	2.047494000	1.778652000
9	-2.170587000	2.839952000	-0.308120000
1	-1.902147000	-2.854933000	0.256557000
1	0.610810000	-2.458253000	0.215419000
53	3.184135000	-0.174334000	0.035556000
9	5.469529000	-2.467515000	0.084808000
9	9.433424000	0.021787000	-0.258306000
9	8.155149000	-2.372734000	-0.042125000
9	5.306997000	2.257703000	-0.220418000
9	7.994123000	2.331048000	-0.346138000
6	5.292950000	-0.107379000	-0.062844000
6	6.058286000	-1.271036000	-0.020487000
6	7.366305000	1.159880000	-0.240127000
6	7.448665000	-1.242669000	-0.084979000
6	8.103715000	-0.019676000	-0.195419000
6	5.976733000	1.101787000	-0.173662000
**Total Energy**		-2043.42117502	

Table S54: Cartesian coordinates and total energy in a.u. of the excited state optimized geometry of **16PN**:C_6F_5Cl complex with the halogen bond at the *heterocyclic* nitrogen at the MN15/aug-cc-pVDZ level of theory.

Atomic Number	X (Å)	Y (Å)	Z (Å)
6	-4.589755000	-1.380261000	0.000236000
6	-4.496955000	0.026729000	0.000025000
6	-5.820846000	-2.001572000	0.000210000
1	-5.870744000	-3.087327000	0.000371000
6	-5.674547000	0.805196000	-0.000217000
6	-7.019709000	-1.222857000	-0.000037000
1	-5.566752000	1.887867000	-0.000370000
6	-6.912892000	0.203930000	-0.000251000
1	-7.807396000	0.821302000	-0.000432000
1	-3.667995000	-1.957974000	0.000415000
7	-8.235126000	-1.825996000	-0.000067000
6	-9.456914000	-1.033548000	-0.000361000
1	-9.506455000	-0.396932000	0.892699000
1	-9.506154000	-0.397146000	-0.893588000
1	-10.315474000	-1.707297000	-0.000427000
6	-8.343618000	-3.278642000	0.000213000
1	-9.399607000	-3.554249000	0.000199000
1	-7.866834000	-3.703346000	-0.892818000
1	-7.866952000	-3.702989000	0.893478000
6	-3.150911000	0.687646000	0.000062000
8	-2.164337000	-0.163271000	0.000240000
7	-3.132276000	1.973335000	-0.000135000
5	-0.731124000	0.307526000	0.000215000

6	-1.879942000	2.623495000	-0.000118000
6	-1.818941000	3.992412000	-0.000287000
7	-0.758770000	1.853577000	0.000078000
6	-0.520551000	4.598323000	-0.000250000
6	0.568473000	3.730048000	-0.000036000
7	0.502043000	2.402209000	0.000128000
9	-0.125300000	-0.179000000	-1.149977000
9	-0.125325000	-0.178800000	1.150501000
1	-2.746423000	4.557237000	-0.000437000
1	-0.378374000	5.675243000	-0.000376000
1	1.589184000	4.116267000	0.000016000
9	3.460346000	-2.345759000	-0.000075000
9	8.156273000	-2.153486000	-0.000295000
9	5.849329000	-3.599138000	-0.000323000
9	5.671248000	1.837076000	0.000236000
9	8.053328000	0.568801000	-0.000013000
6	4.496339000	-0.217960000	0.000086000
6	4.568974000	-1.612910000	-0.000059000
6	6.923973000	-0.139959000	-0.000028000
6	5.798072000	-2.266881000	-0.000187000
6	6.978697000	-1.530350000	-0.000171000
6	5.690839000	0.505334000	0.000100000
17	2.985376000	0.583513000	0.000245000
**Total Energy**		-2208.48974361	

Table S55: Cartesian coordinates and total energy in a.u. of the excited state optimized geometry of **16PN**:C_6F_5Br complex with the halogen bond at the *heterocyclic* nitrogen at the MN15/aug-cc-pVDZ-PP level of theory.

Atomic Number	X (Å)	Y (Å)	Z (Å)
6	4.991871000	-1.388816000	-0.001214000
6	4.804579000	0.008439000	-0.000248000
6	6.262176000	-1.925437000	-0.000954000
1	6.385345000	-3.005299000	-0.001709000
6	5.926281000	0.865060000	0.000987000
6	7.405576000	-1.067267000	0.000305000
1	5.745790000	1.937972000	0.001701000
6	7.202593000	0.349018000	0.001267000
1	8.053201000	1.025561000	0.002237000
1	4.111585000	-2.027903000	-0.002143000
7	8.658868000	-1.586569000	0.000586000
6	9.824365000	-0.713175000	0.001818000
1	9.830756000	-0.074216000	-0.890875000
1	9.829890000	-0.075570000	0.895485000
1	10.726499000	-1.327259000	0.001788000
6	8.865905000	-3.028630000	-0.000338000
1	9.938158000	-3.231714000	0.000153000
1	8.418562000	-3.484982000	0.892261000
1	8.419622000	-3.483690000	-0.894128000
6	3.416128000	0.576972000	-0.000547000
8	2.490254000	-0.339423000	-0.001497000
7	3.309444000	1.858082000	0.000261000
5	1.029140000	0.029844000	-0.000905000

6	2.015767000	2.420969000	0.000018000
6	1.859564000	3.782954000	0.000499000
7	0.952294000	1.575818000	-0.000662000
6	0.521399000	4.297949000	0.000255000
6	-0.504288000	3.359379000	-0.000321000
7	-0.340531000	2.038734000	-0.000743000
9	0.455281000	-0.492008000	1.149036000
9	0.454467000	-0.491730000	-1.150525000
1	2.745135000	4.411129000	0.001050000
1	0.305664000	5.362506000	0.000586000
1	-1.551318000	3.668821000	-0.000429000
9	-3.502533000	-2.437613000	0.000010000
9	-8.177668000	-2.005710000	0.000826000
9	-5.944769000	-3.566392000	0.000524000
9	-5.501656000	1.852861000	0.000097000
9	-7.936690000	0.709059000	0.000608000
6	-4.416570000	-0.253552000	0.000035000
6	-4.570165000	-1.640276000	0.000150000
6	-6.844416000	-0.056851000	0.000460000
6	-5.827717000	-2.237765000	0.000415000
6	-6.969367000	-1.442523000	0.000572000
6	-5.577615000	0.519684000	0.000194000
35	-2.714898000	0.542725000	-0.000310000
**Total Energy**		-2165.66281880	

Table S56: Cartesian coordinates and total energy in a.u. of the excited state optimized geometry of **16PN**:C_6F_5I complex with the halogen bond at the *heterocyclic* nitrogen at the MN15/aug-cc-pVDZ-PP level of theory.

Atomic Number	X (Å)	Y (Å)	Z (Å)
6	5.285985000	-1.418866000	-0.002532000
6	5.085675000	-0.023650000	-0.000213000
6	6.561301000	-1.943526000	-0.002261000
1	6.694586000	-3.022171000	-0.004086000
6	6.198662000	0.843738000	0.002426000
6	7.696416000	-1.074475000	0.000404000
1	6.008458000	1.914968000	0.004167000
6	7.479932000	0.339851000	0.002752000
1	8.324011000	1.024492000	0.004807000
1	4.412179000	-2.066755000	-0.004505000
7	8.954419000	-1.581832000	0.000705000
6	10.111872000	-0.697628000	0.003389000
1	10.112612000	-0.057807000	-0.888678000
1	10.110950000	-0.061045000	0.897770000
1	11.019609000	-1.303342000	0.003136000
6	9.175033000	-3.021993000	-0.001664000
1	10.249107000	-3.215014000	-0.000912000
1	8.731530000	-3.483300000	0.890276000
1	8.733358000	-3.480083000	-0.896167000
6	3.691476000	0.531651000	-0.000572000
8	2.774836000	-0.393764000	-0.002724000
7	3.570292000	1.811102000	0.001122000
5	1.312199000	-0.041148000	-0.001462000

6	2.270830000	2.360230000	0.000521000
6	2.102300000	3.721403000	0.001312000
7	1.216817000	1.505158000	-0.000914000
6	0.759045000	4.223451000	0.000675000
6	-0.257729000	3.278799000	-0.000458000
7	-0.080114000	1.957647000	-0.001213000
9	0.741835000	-0.565668000	1.148593000
9	0.740114000	-0.565031000	-1.150874000
1	2.981854000	4.357664000	0.002438000
1	0.533170000	5.285837000	0.001196000
1	-1.307778000	3.577910000	-0.000803000
53	-2.497783000	0.498018000	-0.000692000
9	-3.608758000	-2.544333000	0.000986000
9	-8.262343000	-1.941083000	0.001577000
9	-6.088271000	-3.581426000	0.001957000
9	-5.456744000	1.820398000	-0.000764000
9	-7.926263000	0.762495000	0.000213000
6	-4.437607000	-0.322670000	0.000081000
6	-4.645318000	-1.701552000	0.000784000
6	-6.860356000	-0.040929000	0.000402000
6	-5.922079000	-2.257216000	0.001293000
6	-7.034281000	-1.421273000	0.001099000
6	-5.572297000	0.487462000	-0.000095000
**Total Energy**		-2043.37040604	

Table S57: Cartesian coordinates and total energy in a.u. of the excited state optimized geometry of **PN**:C_6F_5I complex with the halogen bond at the *imine* nitrogen at the MN15/aug-cc-pVDZ-PP level of theory.

Atomic Number	X (Å)	Y (Å)	Z (Å)
6	2.942752000	2.494971000	0.839319000
6	2.470642000	1.558471000	-0.113878000
6	2.528429000	3.810770000	0.801318000
1	2.901853000	4.504792000	1.550701000
6	1.589940000	2.007895000	-1.133897000
6	1.615333000	4.257520000	-0.201673000
1	1.253546000	1.292581000	-1.881730000
6	1.167745000	3.318450000	-1.178292000
1	0.484728000	3.627523000	-1.965649000
1	3.631217000	2.148711000	1.607600000
7	1.185584000	5.553517000	-0.228302000
6	0.247857000	5.994084000	-1.245390000
1	0.673409000	5.873196000	-2.252256000
1	-0.688824000	5.420443000	-1.191272000
1	0.021835000	7.050743000	-1.086079000
6	1.643067000	6.501923000	0.771205000
1	1.186240000	7.474051000	0.572964000
1	1.357004000	6.178206000	1.782617000
1	2.736463000	6.614036000	0.736985000
6	2.886619000	0.157060000	-0.034581000
8	3.849786000	-0.110703000	0.834370000
7	2.283936000	-0.715562000	-0.823648000
5	3.964132000	-1.501655000	1.322870000

6	2.670232000	-2.026377000	-0.799792000
6	2.155498000	-2.937198000	-1.732926000
7	3.615310000	-2.443363000	0.124091000
6	2.604201000	-4.251359000	-1.756741000
6	3.589644000	-4.647579000	-0.810614000
6	4.054225000	-3.728659000	0.103494000
9	3.028632000	-1.712808000	2.340302000
9	5.260250000	-1.754132000	1.743210000
1	4.796978000	-3.973701000	0.861063000
1	3.978117000	-5.663976000	-0.789599000
1	2.209505000	-4.960298000	-2.482307000
1	1.413501000	-2.565228000	-2.438992000
53	-0.667058000	-0.748616000	-0.408329000
9	-2.916371000	-3.009624000	0.138126000
9	-6.794442000	-0.436553000	0.745658000
9	-5.557494000	-2.858471000	0.638209000
9	-2.722511000	1.715852000	-0.156184000
9	-5.361925000	1.844745000	0.345234000
6	-2.729710000	-0.653548000	-0.022358000
6	-3.487487000	-1.804665000	0.184924000
6	-4.755191000	0.658562000	0.294654000
6	-4.854443000	-1.744542000	0.444316000
6	-5.488817000	-0.506560000	0.499305000
6	-3.390455000	0.571822000	0.036958000
**Total Energy**		-2027.39097662	

Table S58: Cartesian coordinates and total energy in a.u. of the excited state optimized geometry of **13PN**:C_6F_5I complex.

Atomic Number	X (Å)	Y (Å)	Z (Å)
6	-3.540334000	2.679539000	0.000597000
6	-2.601793000	1.629121000	0.000147000
6	-3.114647000	3.991273000	0.000650000
1	-3.849645000	4.792012000	0.000994000
6	-1.217487000	1.914303000	-0.000263000
6	-1.714947000	4.293623000	0.000239000
1	-0.519279000	1.078118000	-0.000602000
6	-0.773643000	3.215356000	-0.000221000
1	0.295763000	3.410904000	-0.000515000
1	-4.599796000	2.432072000	0.000894000
7	-1.287893000	5.579304000	0.000287000
6	0.137234000	5.887517000	-0.000198000
1	0.622504000	5.472043000	0.892013000
1	0.621860000	5.472180000	-0.892821000
1	0.263875000	6.971307000	-0.000158000
6	-2.240607000	6.681623000	0.000800000
1	-1.690580000	7.623995000	0.000850000
1	-2.877482000	6.643020000	-0.892339000
1	-2.876961000	6.642630000	0.894291000
6	-3.052194000	0.199553000	0.000139000
8	-4.344752000	0.038382000	0.000601000
7	-2.115240000	-0.677490000	-0.000231000
5	-4.930892000	-1.344999000	-0.000693000
6	-2.454021000	-2.042745000	0.000088000

7	-1.457360000	-2.879118000	0.000293000
7	-3.769657000	-2.365635000	0.000252000
6	-1.759025000	-4.245533000	0.000638000
6	-3.085089000	-4.663102000	0.000764000
6	-4.104794000	-3.730458000	0.000576000
9	-5.704279000	-1.494929000	-1.147886000
9	-5.706954000	-1.495942000	1.144524000
1	-5.163860000	-3.963194000	0.000708000
1	-3.328845000	-5.724757000	0.001012000
1	-0.919428000	-4.933617000	0.000748000
53	1.060407000	-1.722778000	0.000119000
9	2.032082000	1.374837000	-0.000386000
9	6.709977000	1.014910000	-0.000280000
9	4.443856000	2.536898000	-0.000549000
9	4.108541000	-2.891671000	0.000330000
9	6.517724000	-1.702375000	0.000160000
6	2.975355000	-0.810976000	-0.000017000
6	3.116489000	0.574240000	-0.000245000
6	5.411859000	-0.959184000	0.000029000
6	4.357003000	1.202359000	-0.000334000
6	5.513424000	0.429003000	-0.000196000
6	4.153107000	-1.556600000	0.000114000
**Total Energy**		-2043.40519565	

Table S59: Cartesian coordinates and total energy in a.u. of the excited state optimized geometry of **14PN**:C_6F_5I complex.

**Atomic Number**	**X (Å)**	**Y (Å)**	**Z (Å)**

6	-1.101498000	2.323977000	1.098056000
6	-1.633557000	1.882752000	-0.135429000
6	0.044383000	3.080182000	1.136400000
1	0.462023000	3.375853000	2.094759000
6	-1.043092000	2.314763000	-1.344255000
6	0.707337000	3.433911000	-0.083618000
1	-1.486568000	2.003153000	-2.287713000
6	0.105631000	3.068858000	-1.331685000
1	0.571085000	3.355456000	-2.270552000
1	-1.594778000	2.018304000	2.018836000
7	1.897302000	4.070021000	-0.057129000
6	2.626566000	4.336035000	-1.291667000
1	2.858462000	3.392811000	-1.802565000
1	2.036025000	4.976691000	-1.956888000
1	3.558838000	4.846834000	-1.047394000
6	2.559568000	4.357202000	1.210101000
1	3.498212000	4.874687000	1.007910000
1	1.929282000	4.999365000	1.836066000
1	2.773511000	3.422376000	1.743815000
6	-2.782892000	0.928164000	-0.147224000
8	-3.953573000	1.524107000	-0.238746000
7	-2.506557000	-0.329758000	-0.070048000
5	-5.135090000	0.722133000	0.242339000
6	-3.557730000	-1.241554000	-0.098622000
6	-3.332881000	-2.604837000	-0.156857000
7	-4.854458000	-0.749587000	-0.106237000

7	-4.299332000	-3.533865000	-0.226202000
6	-5.574819000	-3.023098000	-0.238488000
6	-5.861886000	-1.689082000	-0.174737000
9	-6.279964000	1.168999000	-0.396171000
9	-5.210064000	0.897763000	1.632154000
1	-2.297908000	-2.951132000	-0.154873000
1	-6.877312000	-1.300813000	-0.178842000
1	-6.391203000	-3.741942000	-0.301028000
53	0.244381000	-0.994859000	-0.008797000
9	2.233015000	-3.570068000	0.052763000
9	6.483289000	-1.578015000	0.083925000
9	4.911909000	-3.801316000	0.092435000
9	2.669605000	1.153509000	-0.005052000
9	5.331042000	0.899681000	0.034619000
6	2.347687000	-1.206967000	0.022999000
6	2.965433000	-2.456514000	0.047989000
6	4.570099000	-0.199562000	0.039365000
6	4.352276000	-2.595413000	0.068787000
6	5.158872000	-1.460208000	0.064432000
6	3.184503000	-0.095330000	0.019160000
**Total Energy**		-2043.38730409	

Table S60: Cartesian coordinates and total energy in a.u. of the excited state optimized geometry of **15PN**:C_6F_5I complex.

Atomic Number	X (Å)	Y (Å)	Z (Å)
6	1.152812000	2.350582000	0.999332000
6	1.625954000	1.893988000	-0.258308000

6	0.008281000	3.100762000	1.092850000
1	-0.361729000	3.401785000	2.069139000
6	0.976992000	2.335921000	-1.438198000
6	-0.717389000	3.445837000	-0.095290000
1	1.376507000	2.025308000	-2.401687000
6	-0.171334000	3.085473000	-1.371576000
1	-0.682947000	3.369887000	-2.286934000
1	1.699576000	2.052655000	1.892894000
7	-1.908361000	4.070416000	-0.012420000
6	-2.695654000	4.335421000	-1.211637000
1	-2.138969000	4.977772000	-1.903708000
1	-2.951238000	3.392398000	-1.711727000
1	-3.615913000	4.845346000	-0.923168000
6	-2.516335000	4.343267000	1.285508000
1	-3.475431000	4.838601000	1.127863000
1	-2.683762000	3.404584000	1.828818000
1	-1.873168000	5.000874000	1.881821000
6	2.754269000	0.931646000	-0.306803000
8	3.957536000	1.494650000	-0.423578000
7	2.485333000	-0.347952000	-0.177909000
5	5.029544000	0.741870000	0.283828000
6	3.505144000	-1.238411000	-0.119391000
6	3.329669000	-2.637294000	-0.155833000
7	4.818060000	-0.765712000	-0.043257000
6	4.437111000	-3.447557000	-0.158041000
7	5.731672000	-2.954487000	-0.123416000
6	5.840724000	-1.655300000	-0.054791000

1	6.832192000	-1.204973000	-0.003100000
9	4.849854000	0.936202000	1.665678000
9	6.274284000	1.160125000	-0.151105000
1	2.322882000	-3.049916000	-0.198698000
1	4.339026000	-4.531739000	-0.191057000
53	-0.211663000	-0.984023000	-0.067051000
9	-2.185743000	-3.578850000	-0.000970000
9	-6.448637000	-1.619518000	0.161682000
9	-4.861387000	-3.831011000	0.097585000
9	-2.657351000	1.141787000	0.028744000
9	-5.316080000	0.867092000	0.125631000
6	-2.317694000	-1.215875000	0.010796000
6	-2.926370000	-2.469459000	0.029790000
6	-4.546701000	-0.225810000	0.095340000
6	-4.311040000	-2.620119000	0.080680000
6	-5.125620000	-1.491149000	0.113590000
6	-3.162530000	-0.110953000	0.044743000
**Total Energy**		-2043.40098309	

Table S61: Cartesian coordinates and total energy in a.u. of the excited state optimized geometry of **16PN**:C_6F_5I complex.

Atomic Number	X (Å)	Y (Å)	Z (Å)
6	1.048165000	2.314475000	1.306703000
6	1.623952000	1.877860000	0.093423000
6	-0.096577000	3.074975000	1.302396000
1	-0.550659000	3.367992000	2.244830000
6	1.083502000	2.315626000	-1.136368000

6	-0.707967000	3.439304000	0.059176000
1	1.565795000	2.005600000	-2.061340000
6	-0.058970000	3.077997000	-1.165732000
1	-0.483692000	3.373439000	-2.121017000
1	1.500172000	2.004530000	2.246635000
7	-1.894595000	4.083037000	0.041938000
6	-2.566457000	4.371287000	-1.219805000
1	-1.938214000	5.008786000	-1.852623000
1	-2.789979000	3.436553000	-1.749729000
1	-3.500510000	4.894148000	-1.010443000
6	-2.608886000	4.359864000	1.282666000
1	-3.540471000	4.875341000	1.045836000
1	-2.841044000	3.420726000	1.800891000
1	-2.007054000	4.999955000	1.938292000
6	2.775388000	0.921525000	0.101092000
8	3.931508000	1.516617000	0.172772000
7	2.487789000	-0.333647000	0.042407000
5	5.176848000	0.701288000	-0.170307000
6	3.539245000	-1.260899000	0.080333000
6	3.286765000	-2.608102000	0.122399000
7	4.827785000	-0.773121000	0.102388000
6	4.383643000	-3.500725000	0.176326000
6	5.665769000	-2.909687000	0.163253000
7	5.915108000	-1.621448000	0.119515000
9	6.199334000	1.157172000	0.631956000
9	5.422191000	0.920053000	-1.528773000
1	2.254385000	-2.949660000	0.118531000

1	4.260655000	-4.578766000	0.211356000
1	6.561300000	-3.533351000	0.184396000
53	-0.250100000	-0.991417000	0.002003000
9	-2.679919000	1.152096000	0.001955000
9	-6.490445000	-1.584993000	-0.055585000
9	-5.341518000	0.894198000	-0.022874000
9	-2.237265000	-3.571328000	-0.038170000
9	-4.915972000	-3.806405000	-0.062824000
6	-2.354986000	-1.208017000	-0.017721000
6	-3.193313000	-0.097485000	-0.014255000
6	-4.358058000	-2.599409000	-0.047194000
6	-4.578924000	-0.203691000	-0.026831000
6	-5.166134000	-1.465265000	-0.043473000
6	-2.971484000	-2.458044000	-0.034274000
**Total Energy**		-2043.35849112	
